# An atlas of cells in the human tonsil

**DOI:** 10.1016/j.immuni.2024.01.006

**Published:** 2024-02-13

**Authors:** Ramon Massoni-Badosa, Sergio Aguilar-Fernández, Juan C. Nieto, Paula Soler-Vila, Marc Elosua-Bayes, Domenica Marchese, Marta Kulis, Amaia Vilas-Zornoza, Marco Matteo Bühler, Sonal Rashmi, Clara Alsinet, Ginevra Caratù, Catia Moutinho, Sara Ruiz, Patricia Lorden, Giulia Lunazzi, Dolors Colomer, Gerard Frigola, Will Blevins, Lucia Romero-Rivero, Víctor Jiménez-Martínez, Anna Vidal, Judith Mateos-Jaimez, Alba Maiques-Diaz, Sara Ovejero, Jérôme Moreaux, Sara Palomino, David Gomez-Cabrero, Xabier Agirre, Marc A. Weniger, Hamish W. King, Lucy C. Garner, Federico Marini, Francisco Javier Cervera-Paz, Peter M. Baptista, Isabel Vilaseca, Cecilia Rosales, Silvia Ruiz-Gaspà, Benjamin Talks, Keval Sidhpura, Anna Pascual-Reguant, Anja E. Hauser, Muzlifah Haniffa, Felipe Prosper, Ralf Küppers, Ivo Glynne Gut, Elias Campo, José Ignacio Martin-Subero, Holger Heyn

**Affiliations:** 1 Centro Nacional de Análisis Genómico (CNAG), Barcelona, Spain; 2 Institut d’Investigacions Biomèdiques August Pi i Sunyer (IDIBAPS), Barcelona, Spain; 3 Hemato-Oncology Program, Center for Applied Medical Research (CIMA), University of Navarra, IDISNA, Universidad de Navarra, Pamplona, Spain; 4 Centro de Investigación Biomédica en Red Cáncer (CIBERONC), Madrid, Spain; 5 Department of Pathology and Molecular Pathology, University Hospital Zurich, Zurich, Switzerland; 6 Hematopathology Section, Pathology Department, Hospital Clinic, Barcelona, Spain; 7 Departament de Fonaments Clínics, Facultat de Medicina, Universitat de Barcelona, Barcelona, Spain; 8 Department of Biological Hematology, CHU Montpellier, Montpellier, France; 9 Institute of Human Genetics, UMR 9002 CNRS-UM, Montpellier, France; 10 Department of Clinical Hematology, CHU Montpellier, Montpellier, France; 11 Translational Bioinformatics Unit (TransBio), Navarrabiomed, Navarra Health Department (CHN), Public University of Navarra (UPNA), Navarra Institute for Health Research (IdiSNA), Pamplona, Spain; 12 Bioscience Program, Biological and Environmental Sciences and Engineering Division (BESE), King Abdullah University of Science and Technology KAUST, Thuwal, Saudi Arabia; 13 Institute of Cell Biology (Cancer Research), Medical Faculty, University of Duisburg-Essen, Essen, Germany; 14 Epigenetics and Development Division, Walter and Eliza Hall Institute, Parkville, Australia; 15 Translational Gastroenterology Unit, Nuffield Department of Medicine, University of Oxford, Oxford, UK; 16 Institute of Medical Biostatistics, Epidemiology and Informatics (IMBEI), University Medical Center of the Johannes Gutenberg University Mainz, Mainz, Germany; 17 Center for Thrombosis and Hemostasis (CTH), University Medical Center of the Johannes Gutenberg University Mainz, Mainz, Germany; 18 Department of Otorhinolaryngology, University of Navarra, Pamplona, Spain; 19 Otorhinolaryngology Head-Neck Surgery Department, Hospital Clínic, IDIBAPS Universitat de Barcelona, Barcelona, Spain; 20 Biosciences Institute, Newcastle University, Newcastle Upon Tyne, UK; 21 Department of Otolaryngology, Freeman Hospital, Newcastle Hospitals NHS Foundation Trust, Newcastle Upon Tyne, UK; 22 Department of Rheumatology and Clinical Immunology, Charité - Universitätsmedizin Berlin, Berlin, Germany; 23 Immune Dynamics, Deutsches Rheuma-Forschungszentrum (DRFZ), Berlin, Germany; 24 Wellcome Sanger Institute, Wellcome Genome Campus, Cambridge, UK; 25 Department of Dermatology and NIHR Newcastle Biomedical Research Centre, Newcastle Hospitals NHS Foundation Trust, Newcastle Upon Tyne, UK; 26 Departamento de Hematología, Clínica Universidad de Navarra, University of Navarra, Pamplona, Spain; 27 Universitat Pompeu Fabra (UPF), Barcelona, Spain; 28 Institució Catalana de Recerca i Estudis Avançats (ICREA), Barcelona, Spain

**Keywords:** human tonsil, secondary lymphoid organs, adaptive immunity, innate immunity, aging, mantle cell lymphoma, Human Cell Atlas, single-cell genomics, spatial transcriptomics

## Abstract

Palatine tonsils are secondary lymphoid organs (SLOs) representing the first line of immunological defense against inhaled or ingested pathogens. We generated an atlas of the human tonsil composed of >556,000 cells profiled across five different data modalities, including single-cell transcriptome, epigenome, proteome, and immune repertoire sequencing, as well as spatial transcriptomics. This census identified 121 cell types and states, defined developmental trajectories, and enabled an understanding of the functional units of the tonsil. Exemplarily, we stratified myeloid slan-like subtypes, established a *BCL6* enhancer as locally active in follicle-associated T and B cells, and identified SIX5 as putative transcriptional regulator of plasma cell maturation. Analyses of a validation cohort confirmed the presence, annotation, and markers of tonsillar cell types and provided evidence of age-related compositional shifts. We demonstrate the value of this resource by annotating cells from B cell-derived mantle cell lymphomas, linking transcriptional heterogeneity to normal B cell differentiation states of the human tonsil.

## Highlights

• Single-cell atlas of the human tonsils as a model for secondary lymphoid organs
• Comprehensive glossary of 121 cell types and states defined by multimodal profiling
• High-resolution immune cell activation landscape with lineage-defining regulators
• A FAIR resource accessible through HCATonsilData

## Graphical abstract

**Figure:**
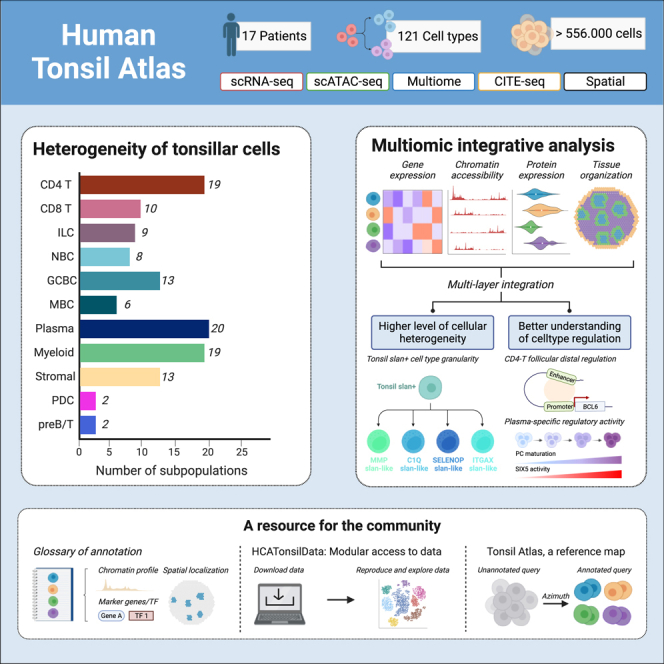

## Teaser

Massoni-Badosa et al. present a comprehensive human tonsil cell atlas, identifying 121 cell types and states through multimodal single-cell profiling. This atlas elucidates cell differentiation pathways and regulatory circuits, defines cell states, and provides a reference for annotating immune cell types and characterizing phenotypic plasticity in pathological settings such as lymphoid neoplasms.

## Introduction

Palatine tonsils are under constant exposure to antigens via the upper respiratory tract, which makes them a compelling model secondary lymphoid organ (SLO) to study the interplay between innate and adaptive immune cells.^1^ Within tonsil crypts, microfold cells (or M cells) sample antigens at their apical membrane. Subsequently, antigen-presenting cells (APCs), such as dendritic cells (DCs), process and present antigens to T cells in the interfollicular or T cell zone. Alternatively, antigens are kept intact by follicular DCs (FDCs) in lymphoid follicles, where they are recognized by B cells.^2^ Such recognition triggers the germinal center (GC) reaction, whereby naive B cells (NBCs) undergo clonal selection, proliferation, somatic hypermutation, class switch recombination (CSR), and differentiation into long-lived plasma cells (PCs) or memory B cells (MBCs).^3^ Thus, a granular taxonomy of cell types and states is needed to fully grasp the heterogeneity of tonsillar cells.

The discriminative power of single-cell RNA sequencing (scRNA-seq) has catalyzed the creation of cellular taxonomies of hematopoietic organs, such as the thymus^4^ and the bone marrow.^5^^,^^6^ In the context of the Human Cell Atlas (HCA),^7^ these taxonomies identify cell types and provide a reference to annotate cell types and states by training classifiers^8^^,^^9^ and through curated cell ontologies.^10^ While the transcriptome allows for precise cellular phenotyping, recent atlases also incorporate additional layers, such as the epigenome or spatial profiles. Together, such complementary modalities contribute multiple layers to define cell identities.^11^ Single-cell profiling efforts of the human tonsil provided insights into specific cell populations (e.g., B cells^12^^,^^13^ or innate lymphoid cells [ILCs]^14^), but they lacked sufficient cell numbers and multimodal information to fully capture the cellular complexity of the organ.

Here, we generated a human tonsil atlas composed of >556,000 cells profiled across 5 different data modalities, including transcriptome, epigenome, proteome, adaptive immune repertoire, and spatial location. We identified 121 cell types and states, connected through a continuum of gene regulatory events and spatial co-localization to form the functional units of the human tonsil. We validated tonsillar cell-type annotations and marker genes in an independent validation cohort, which also identified age-related compositional shifts. Finally, we showcase that the tonsil atlas provides a reference to characterize phenotypic plasticity in lymphoid neoplasms by interrogating the intratumoral heterogeneity of mantle cell lymphomas (MCLs)^15^ that frequently presents in the tonsil.^16^ Together, our atlas represents a comprehensive census of cell types and states as building blocks of the human tonsil and serves as a blueprint to chart organ complexity and to annotate normal and diseased cells of SLO.

## Results

### A single-cell multiomic atlas of human tonsillar cells

**Figure 1:**
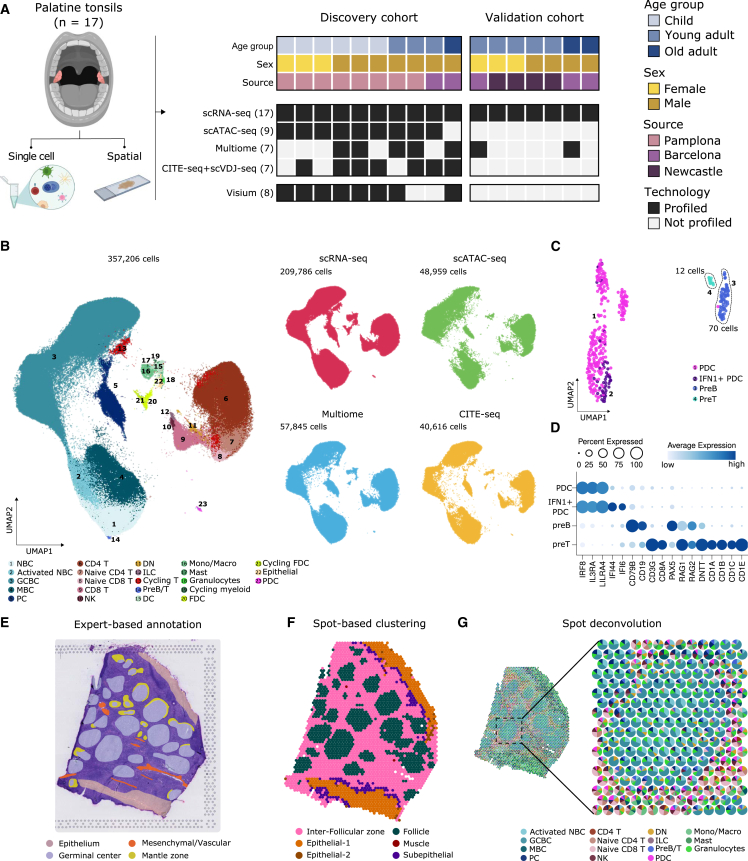
A single-cell multiomic atlas of human tonsillar cells (A) Schematic diagram of the multiomic approach in both the discovery and validation cohorts. (B) Uniform manifold approximation and projection (UMAP) of the 357,206 tonsillar cells analyzed. Left: colored and numbered by the main 23 populations. Right: split by data modality. (C) UMAP of tonsillar plasmacytoid dendritic cells (PDCs) and precursor B and T cells (preB/preT clusters). (D) Dotplot showing average gene expression of marker genes of PDC, preB, and preT clusters. (E) Representative histologically annotated slide of a human tonsil. (F) Gene expression-based clusters of spatial transcriptomics (ST) spots. (G) Spatial scatter pie plot showing each ST spot as a pie chart representing the predicted proportion of cell types.

To create a comprehensive census of tonsillar cells, we sequenced the transcriptome of over 377,000 unselected cells from 17 human tonsils by scRNA-seq. These tonsils covered three age groups—children (n = 6, 3–5 years), young adults (n = 8, 19–35 years), and old adults (n = 3, 56–65 years)—collected in a discovery and validation cohort (Figure 1A; Table S1; see STAR Methods). We used the discovery cohort to comprehensively annotate tonsillar cell types, which we subsequently validated. We complemented transcriptional profiles with single-cell-resolved open chromatin (scATAC-seq and scRNA-seq/ATAC-seq; i.e., Multiome), protein (CITE-seq^17^), adaptive repertoire (single-cell B receptor sequencing [scBCR-seq] and T cell receptor sequencing [TCR-seq]), and spatial transcriptomics (ST) profiles (Figure 1A; Tables S1 and S2). Initially, we created high-level visualization and annotation of all cells across technologies by integrating high-quality transcriptome profiles from scRNA-seq and Multiome (Figure 1B). Our integration strategy (see STAR Methods) removed major technical variability (Figures S1A and S1B) and preserved biological heterogeneity, highlighted by integrating an external, well-annotated dataset of ∼35,000 tonsillar cells (Figures S1A and S1C).^12^

Following Louvain clustering, we first assigned cells into 9 broad groups and 23 general subgroups (Figures 1B and S1D). Naive CD8 T cells shared similar transcriptomes with naive CD4 T cells^18^, and NBCs with MBCs.^19^ In addition, we observed subsets of proliferative cells in several clusters (Figures 1B and S1E). Inside the plasmacytoid DC (PDC) cluster, we identified two intriguing additional clusters of precursor T cells (preT; *CD3G*, *CD8A*) and precursor B cells (preB; *CD19*, *CD79B*, *PAX5*), likely because PDCs develop from common lymphoid progenitors (Figures 1C, 1D, and S1D; Table S3).^20^ Both preT and preB cells expressed members of the VDJ recombinase, including *RAG1*, *RAG2*, and *DNTT* (TdT), supporting T and B cell development within human tonsils (Figure 1D).^21^^,^^22^ PreT cells further expressed several components of the CD1 family of major histocompatibility complex (MHC) class I-like genes (Figure 1D; Table S3). PreB and preT clusters were composed of only 70 (0.033%) and 12 cells (0.0057%), respectively, highlighting the high discriminatory power of our atlas (Figure S1F).

Subsequently, we followed a recursive, top-down, clustering approach, resulting in a total of 121 clusters, which we thoroughly annotated across modalities (see STAR Methods and Figure 1B). Notably, we also minimized batch effects in the scATAC-seq and Multiome datasets, as validated by a decreased local inverse Simpson’s index (LISI) across confounders (Figures S1G and S1H). Our integration yielded high cell-type prediction probabilities (Figure S1I; see STAR Methods). We further integrated single-cell with ST profiles with spot-based clustering, expert annotation, and spot deconvolution, identifying the main histological areas of tonsils and the main tonsillar cell types (Figures 1E–1G). Together, the discovery cohort includes 357,206 cells (209,786 scRNA-seq, 48,959 scATAC-seq, 57,845 Multiome, 40,616 CITE-seq; Figure 1B) and 16,224 ST spots (Figures 1E–1G), which provided the basis for generating a comprehensive resource of annotated cell types and states in the human tonsil.

### Early CD4^+^ T cell fate decision in the human tonsil

**Figure 2:**
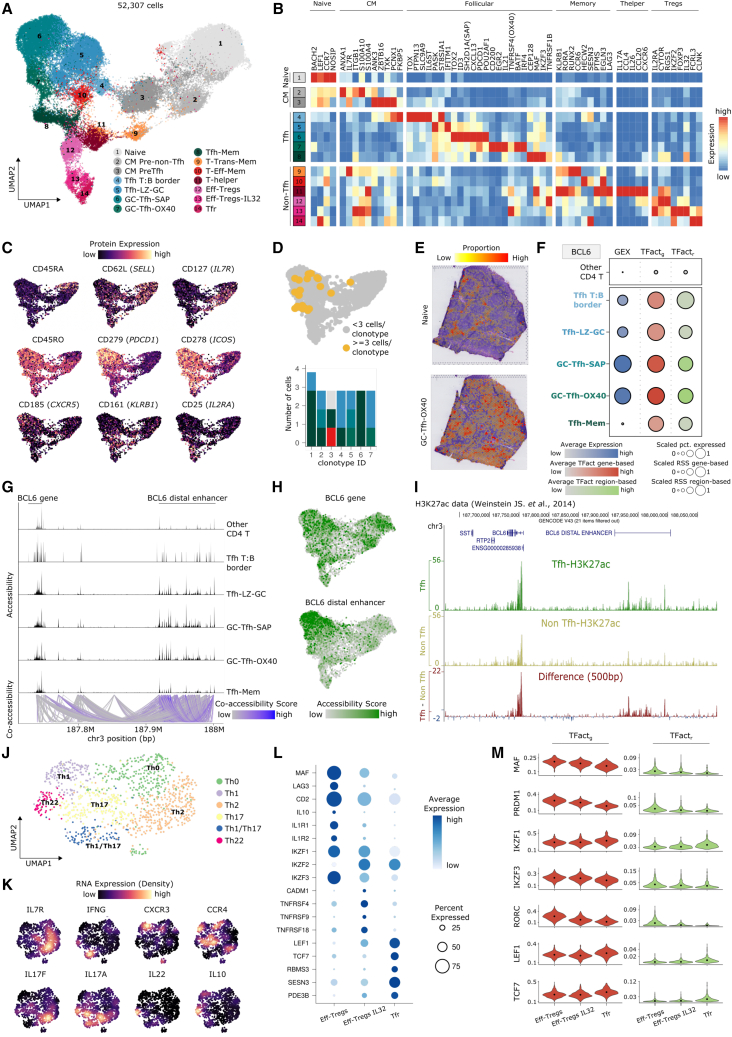
CD4 T follicular and non-follicular cell fate decision in the human tonsil (A) UMAP of tonsillar CD4 T cells colored and numbered by scRNA-seq clusters. (B) Heatmap showing scaled mean marker expression by subpopulation. (C) UMAPs colored by protein expression of canonical phenotype markers of CD4 T cells. (D) Clonal expansion and diversity analysis in CD4 T cells. Top: UMAP showing clonal expansion denoted by ≥3 cells having identical complementarity determining region (CDR)3 sequence (yellow). Bottom: barplot of CD4 T cell subpopulations distribution across the top seven most expanded clonotypes. Color code as in UMAP in (A). (E) Predicted proportions of CD4 T subpopulations. (F) Dotplot showing BCL6 gene expression (blue) and TF activity gene-based (red) and region-based (green). (G) Accessibility and co-accessible links at the *BCL6* locus across Tfh subsets and other CD4 T cells combined. (H) UMAP showing the accessibility score of *BCL6* gene (gene body + 2 kb upstream) (top) and the distal enhancer (bottom). (I) Visualization of H3K27ac signal in BCL6 and the BCL6 distal enhancer. Signal represents absolute values for Tfh (top) and non-Tfh cells (bottom). (J) UMAP of tonsillar Th cells colored by the six scRNA-seq clusters. (K) UMAPs highlighting the estimated expression for key interleukin and chemokine receptors. (L) Dotplot showing expression for the top 18 genes of Treg subpopulations. (M) Violin plots showing gene-based (red) and region-based (green) eRegulon activity for the top TF in Eff-Tregs, Eff-Tregs-IL-32, and Tfr.

T follicular helper (Tfh) cell specification begins with DC presenting antigens to naive CD4 T cells, which subsequently activate and differentiate into central memory (CM) CD4 T cells. We identified two subclusters of CM T cells (Figures 2A–2C; Table S3). Intriguingly, one CM CD4 T cell population expressed higher levels of follicular genes (e.g., *IL6ST*), suggesting early signals of Tfh differentiation (CM pre-Tfh cells; Figures 2B and S2A; Table S3).^23^ We classified the remaining CD4 T cell clusters into Tfh or non-Tfh cells, based on the activity of *BCL6* and *PRDM1* (Figures 2A and S2B; Table S4),^24^^,^^25^ their respective master regulators.^26^ In line, we observed clonal expansion exclusively in Tfh cells (Figure 2D).

We identified a CD4 T cluster that expressed low *CCR7* and high *IL6ST* and *TOX*, pointing to cells migrating to the border of the follicle, primed to interact with B cells via ICOS-ICOSL (Tfh T:B border; Figures 2A, 2B, and S2C). Following the Tfh cell migration trajectory, we identified a Tfh light zone GC (Tfh-LZ-GC) cluster with early signs of GC Tfh differentiation (Figures 2A and 2B). Tfh-LZ-GC cells expressed interleukin-21 (*IL-21*), an inducer of early GC Tfh differentiation (Figures 2B and S2C).^27^ We further identified two clusters of terminal state differentiation and polarization of CD4 Tfh cells (Figures 2A–2C and S2C). Here, the high expression of *SH2D1A* (SAP) identified one subpopulation as a potent GC B cell (GCBC) state inducer (GC Tfh-SAP; Figures 2A and 2B).^27^ In contrast, the second cluster expressed *TNFRSF4* (OX40; GC Tfh-OX40; Figures 2A and 2B) to interact with B cells via OX40-OX40L.^28^ Finally, we observed a cluster of Tfh memory cells, a controversial subtype of follicular T cells (Figures 2A–2C).^29^ Tfh memory cells retained stable expression of *PDCD1*, *MAF*, *CXCR5*, and upregulated *KLRB1*, preserving highly functional follicular characteristics (Figures 2B, 2C, and S2C). The memory phenotype was confirmed at protein level with the higher expression of CD45RO and CD161 (*KLRB1*) as well as by retaining the protein expression of PD-1 and ICOS (Figure 2C). This molecular setup provides further support for the capacity of Tfh memory cells to reenter the Tfh differentiation process. Distinct Tfh cell states broadly mapped to their respective spatial compartments (Figure 2E).

Although BCL6 showed transcriptional and regulatory activity in Tfh cells (Figures 2F and S2B), it was invariably accessible in both Tfh and non-Tfh cell fate trajectories (Figure 2G). This suggested that alternative mechanisms drive BCL6 activity. The inferred regulatory activity strongly connected the *BCL6* gene promoter to an adjacent region with a Tfh-specific accessibility profile (Figure 2G). In line, an accessibility signature derived from open chromatin peaks specific to the *BCL6 cis*-regulatory region was particularly enriched in terminally differentiated Tfh cells (Figures 2H and S2D), a result also found and reported in an independent dataset (Figure S2E).^13^ The distal enhancer further showed activating histone modifications (H3K27ac) enriched in Tfh cells (Figure 2I).^30^ Interestingly, the Tfh-specific *cis*-regulatory region has been previously described to control *BCL6* expression in GCBC,^31^ suggesting the distal enhancer to be a master regulator in GC function across T and B cell lineages.

Compared with naive T cells, CM pre-non-Tfh cells expressed higher levels of CD28 and CD29 (Figure S2F). Transitional memory (T-Trans-Mem) cluster markers (upregulation of *IL-7R* and downregulation of *CCR7* and CD45RA; Figures 2A–2C and S2C) supported an intermediate state between CM pre-non-Tfh cells and fully differentiated T-Eff-Mem cells. Further differentiated clusters split into T-Eff-Mem and different CD4 T helper cell types (Figures 2A–2C). We re-clustered T helper cells and visualized^32^ their interleukin and chemokine receptor expression to further guide cell-type assignment (Figures 2J, 2K, and S2G; Table S3).

We next identified three subtypes of CD4 T regulatory (Treg) cells in tonsils (Figure 2A). Effector Tregs (Eff-Tregs) expressed canonical Treg markers (Figures 2B, 2C, 2L, 2M, and S2C; Table S3) and had increased transcription factor (TF) activity of PRDM1, RORC, MAF family, and IKZF3 (Figure 2M; Table S4).^33^ A second Eff-Treg population expressed higher levels of *IL-32* (Eff-Tregs-IL-32, Figure 2B), a proinflammatory molecule previously linked to suppressing anti-tumor responses (Figure 2B).^34^ The third subpopulation, T follicular regulatory cells (Tfrs), downregulated *FOXP3*, *IL-2RA* (CD25), and *PRDM1* (Figures 2B, S2B, and S2C; Table S3). Tfr cells further presented increased naive markers, in concordance with an increased TF activity of LEF1 and TCF7 (Figures 2L and 2M).^35^ Intriguingly, the top marker *FCRL3* can bind secretory IgA to suppress the Tfr inhibitory function (Figure 2B).^36^ All subtypes of Tregs showed increased signatures in the respective Treg subpopulations (Figure S2H).^37^

### Tissue-resident CD8^+^ T cells in the subepithelial connective tissue septum lining tonsillar crypts

**Figure 3:**
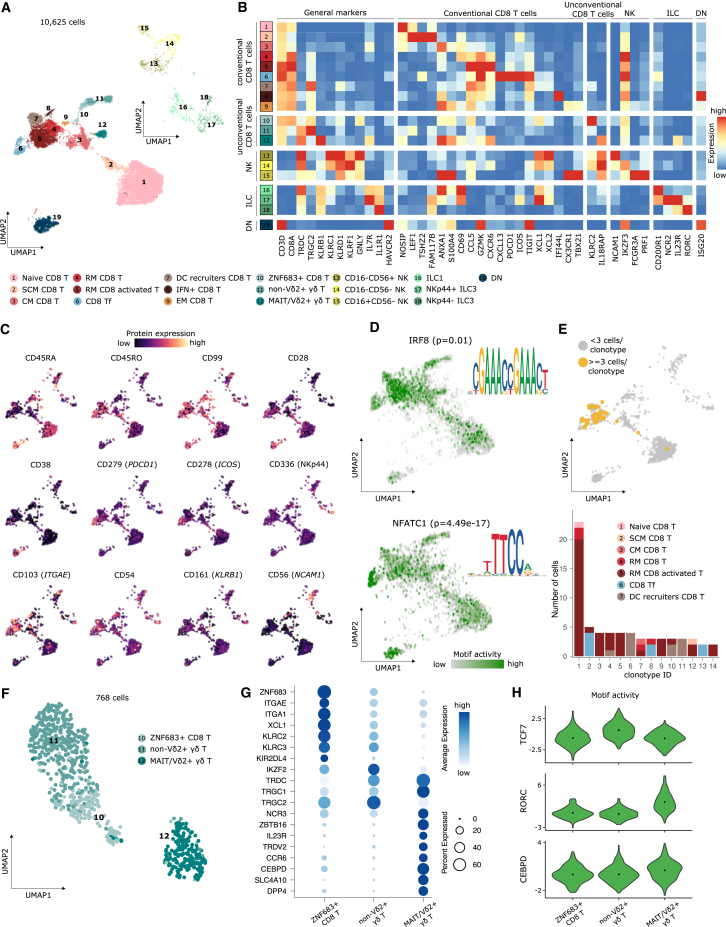
Landscape of CD8 and ILCs in the human tonsil (A) UMAP of tonsillar CD8 T cells (bottom) and ILC (top) colored and numbered by scRNA-seq clusters. (B) Heatmap showing scaled mean marker expression per subpopulation. (C) UMAPs highlighting the protein expression of canonical phenotype markers of CD8 T and ILC. (D) UMAP highlighting the motif activity of IRF8 and NFATC1. The p value represents the significance of the pairwise differential motif analysis performed for each TF. DNA sequence motifs’ logos for each TF. (E) Clonal expansion and diversity analysis in CD8 T cells. Top: UMAP showing clonal expansion denoted by ≥3 cells having identical CDR3 sequence (yellow). Bottom: barplot of CD8 T cell subpopulations distribution across the top 14 most expanded clonotypes. (F) UMAP of tonsillar unconventional CD8 T cells colored and numbered by scRNA-seq clusters. (G) Dotplot showing expression for the top 19 genes for unconventional CD8 T cells. (H) Violin plots of the motif activity score for the top three TF motifs in unconventional CD8 T subpopulations.

We identified a large CD8^+^ T naive subpopulation, which after antigen encounter initiates a program of effector differentiation and a subsequent formation of memory states (Figures 3A–3C; Table S3). Recently formed memory populations are organized in a differentiation hierarchy, from stem cell memory T cells (SCM CD8 T) that self-renew and generate long-lived CM T cells (CM CD8 T; Figures 3A, S3A, and S3B; Table S3).^38^ The chemokine receptor CX3CR1 marks the differentiation from CM CD8 T to effector memory T cells (EM CD8 T), a process tightly controlled by *TBX21* (TBET).^39^ Consistently, EM CD8 T cells expressed the highest levels of *CX3CR1* and *TBX21* across all CD8 T subsets, and TBET motif activity gradually increased from naive to EM CD8 T cell states (Figures 3A, 3B, and S3C).

Subsequently, we identified two clusters of resident memory (RM) CD8 T cells, marked by the expression of the tissue residency markers *ITGA1* and *ITGAE* (CD103; Figures 3A, 3C, and S3A). One of these RM CD8 T cells additionally expressed the activation markers *HLA-DRB1* and *HLA-DPA1* (Figure S3A; Table S3).^40^^,^^41^ Using multiplexed immunofluorescence histology, we identified tonsillar RM CD8 T within the epithelium and in the subepithelial connective tissue septum lining the tonsillar crypts, a preferential localization site also for other tissue-resident immune populations, such as long-lived PC and ILC (Figures S3D–S3G).^42^

Next, we identified a cluster of CD8 T cells that expressed follicular markers (CD8 Tf; Figures 3A–3C; Table S3).^43^ CD8 Tf cells had specific open chromatin peaks enriched with NFATC-family TF motifs, revealed by pairwise differential motif activity analysis against CD8 naive T cells and RM CD8 T cells (Figure 3D; Table S5). Conversely, IRF8, IRF9, and IRF7 motifs were specifically enriched in RM CD8 T cells, which were also the most clonally expanded CD8 T cell subset (Figures 3D and 3E; Table S5). CD8 T cells can instruct PDC recruitment, represented by a CD8 T cell cluster expressing *CCL4*, *XCL1*, and CD99 (Figures 3A–3C and S3A; Table S3).^44^^,^^45^

In the unconventional T cell compartment, one cluster expressed markers of both mucosal-associated invariant T cells (MAIT) and CD161^+^Vδ2^+^ γδ T cells, in line with their reported phenotypic similarity (Figures 3A, 3B, 3F, and 3G).^46^^,^^47^ Both cell types can be activated in a TCR-independent manner and are regulated by PLZF (*ZBTB16*),^47^ a highly specific marker for this cluster (Figure 3G). We also annotated a cluster of non-Vδ2^+^ γδ T (Figures 3F and 3G), with higher motif activity of TCF7 and decreased activity of *RORC* and *CEBPD* (Figure 3H). A third type of unconventional cells (ZNF683^+^ CD8 T) expressed Hobit (*ZNF683*), markers of tissue residency (*ITGAE* and *ITGA1*), natural killer (NK) cell receptors, and CD56 (*NCAM1*; Figures 3A, 3B, 3F, 3G, and S3B). We also identified TIM3^+^ (*HAVCR2*) double-negative (DN; CD8^−^CD4^−^) T cells with a profile of proinflammatory activation (Figures 3A–3C; Table S3).^48^ Considering that CD4 transcripts are frequently undetectable in scRNA-seq data due to technical limitations, we validated the presence of DN CD8^−^CD4^−^ T cells at the protein level, using our CITE-seq data and with additional flow cytometry experiments (Figures S3H–S3L).

NK cells and ILCs differed in their expression of *KLRF1* and *IL-7R* (CD127), respectively (Figures 3A, 3B, and S3B). NK cells followed a differentiation path guided by the reciprocal expression of CD16 (*FCGR3A*) and CD56 (*NCAM1*)—starting from CD16^−^CD56^+^ NK precursors (*SELL*), an intermediate state of CD16^−^CD56^dim^ (*IKZF3*), and ending in a CD16^+^CD56^−^ state with high cytotoxic potential (*PRF1*, *CX3CR1*, and *TBX21*; Figures 3A–3C and S3A).^49^^,^^50^ ILC1 cells could be distinguished from precursor NK cells by their higher expression of *CD200R1* (Figure 3B).^51^ Two remaining ILC clusters could be annotated as NKp44^+^ ILC3 and NKp44^−^ ILC3, in line with the most recent classification of the International Union of Immunological Societies (IUIS; Figures 3B, 3C, and S3A).^52^

### Transient epigenetic reprogramming in LZ-to-DZ B cell transition

**Figure 4:**
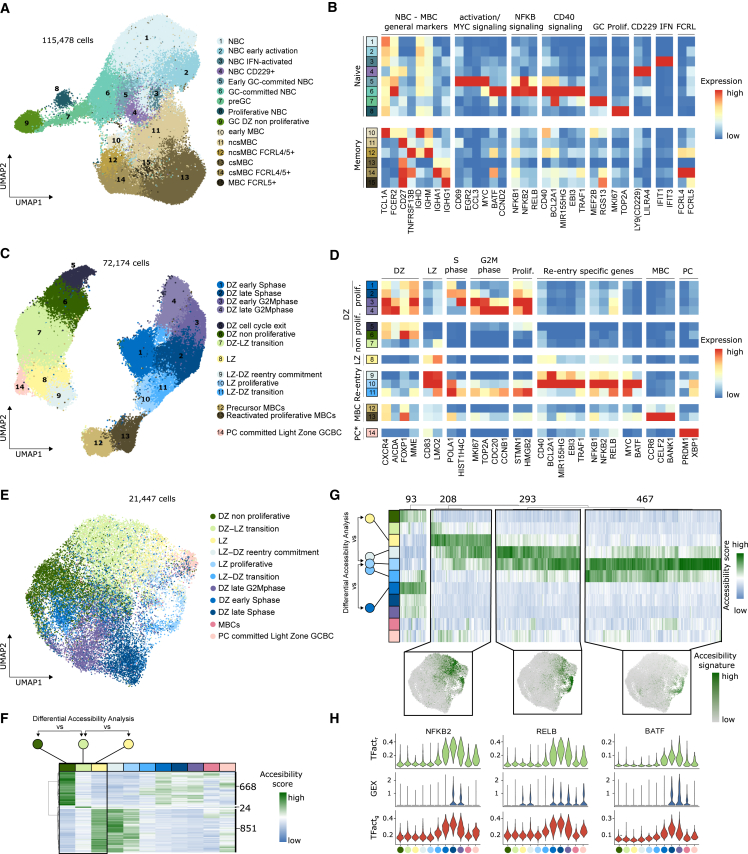
B cell activation and GC dynamics (A) UMAP of tonsillar NBC and MBC B cells colored and numbered by scRNA-seq clusters (including GC DZ non-proliferative, C). (B) Heatmap showing scaled mean marker expression per NBC and MBC subpopulations. (C) UMAP of tonsillar GCBCs colored and numbered by scRNA-seq clusters. (D) Heatmap showing scaled mean marker expression per GCBC subpopulations (including PC-committed light zone GCBC, Figure 5A). (E) UMAP of GCBC colored by scATAC-seq clusters. (F) Heatmap showing normalized accessibility scores of the DARs in the DZ-to-LZ transition (DZ no proliferative → DZ-LZ transition → LZ). Numbers of DARs indicated. (G) Top: heatmap showing normalized accessibility score of the DARs in the LZ-to-DZ reentry (LZ → LZ-DZ reentry commitment → LZ-proliferative → LZ-DZ transition → DZ early S phase). Numbers of DARs indicated. Bottom: UMAP highlighting the accessibility signature scores for each of the main clusters. (H) Violin plots showing gene expression (blue) and gene-based (red) and region-based (green) eRegulon activity for the top TF enriched in each of the clusters (G).

We used established markers to distinguish NBC from MBC, both of which could be subdivided into several states (Figures 4A and 4B). We annotated six MBC subpopulations based on their immunoglobulin (Ig) isotype (class switch IgA/G vs. non-class-switch IgM/D), and the expression of *FCRL4/5* (Figures 4A, 4B, and S4A), consistent with previous studies.^53^^,^^54^

In turn, we divided NBCs into eight subpopulations. To map the NBC-to-GCBC transition, we also integrated non-proliferative dark zone GCBC (DZ-GCBCs). In addition to resting NBCs, we identified a cluster of early-activated NBCs, which showed a moderate upregulation of *CD69* (Figures 4A, 4B, and S4A) and two NBC subclusters that expressed interferon-induced genes (*IFIT1* and *IFIT3*) and *LILR4A/LY9* (CD229), respectively (Figures 4A, 4B, and S4A). An early GC-committed subpopulation expressed high levels of *MYC*, *CD69*, *EGR2/3*, and *CCL3/4* (Figures 4A, 4B, and S4A).^55^ Following this differentiation trajectory, we identified GC-committed cells, characterized by *CCND2* (a downstream target of MYC), *TRAF1/4*, and *MIR155HG*, and pre-GC cells, which showed early seeds of GC-specific genes (*MEF2B* and *RGS13*; Figures 4A, 4B, and S4A).^12^ Interestingly, we detected a subpopulation of proliferative cells with a NBC transcriptome lacking any GC marker (Figures 4A, 4B, and S4A), which may correspond to the primary focal reaction upon very early antigen stimulation leading to the generation of MBCs in a GC-independent manner.^56^^,^^57^

In the GCBC compartment, transcriptomic variability was driven by gene expression differences between DZ and light zone (LZ) and by cell-cycle phases (Figures 4C, 4D, S4B, and S4C). The observed GCBC states were conserved after correcting for cell-cycle differences (Figures S4D and S4E). This suggests that proliferation has a strong effect on GCBC identity beyond *bona fide* cell-cycle genes, in line with previous analyses.^12^ We observed subclusters of early MBCs^58^ and early PCs derived from LZ-GCBCs, and we identified a population that may reflect reactivated MBCs that differentiate into PCs (Figures 4C, 4D, and S4B).^59^

We next studied the cyclic dynamics between the DZ and the LZ.^3^ We identified DZ cells that gradually decrease the expression of cell-cycle genes and transit through an intermediate DZ-LZ phenotype, before giving rise to LZ-GCBCs (Figures 4C, 4D, and S4B). For LZ-GCBCs, we observed several subclusters related to the reentry into the DZ: first, a population with transitory expression of *MYC*, *BATF*, *MIR155HG*, and *TRAF1/4*; second, proliferative cells maintaining the LZ phenotype but upregulating S phase genes; and third, proliferative cells with an intermediate LZ-DZ phenotype, showing loss of CD83 and upregulation of cell-cycle progression genes (Figures 4C, 4D, and S4B).

To epigenetically characterize DZ-to-LZ and LZ-to-DZ transitions, we label-transferred the GCBC transcriptional subclusters onto the chromatin accessibility profiles (see STAR Methods and Figure 4E). The DZ-to-LZ transition was seamless, with most of the DZ- and LZ-specific differentially accessible regions showing an intermediate level (Figure 4F). In sharp contrast, we observed widespread epigenetic reprogramming in the LZ-to-DZ transition. We identified three main modules that transiently increased chromatin accessibility as LZ-GCBCs dedifferentiate through different subclusters to return to the DZ (Figure 4G). The first module was enriched in binding sites and activity of nuclear factor (NF)-κB family TFs, which were gradually replaced by activator protein 1 (AP-1) family footprints (Figures 4H; Tables S4 and S5). The third module was enriched in binding sites and activity of basic leucine zipper transcription factor, ATF-like (BATF), which controls AID (*AICDA*) expression in DZ-GCBCs and is involved in the CSR process.^60^ These results suggest a transient epigenetic programming to be necessary for LZ-GCBCs to return to the DZ. Of note, NF-κB activation followed by BATF upregulation was also observed in activated NBCs differentiating into DZ-GCBCs (Figures S4F and S4G). These results confirm and extend previous observations focused on *MYC*,^61^ suggesting that similar molecular mechanisms are necessary for a B cell, either NBC or LZ-GCBC, to become a DZ-GCBC.

### PC-specific activity of the SIX5 TF

**Figure 5:**
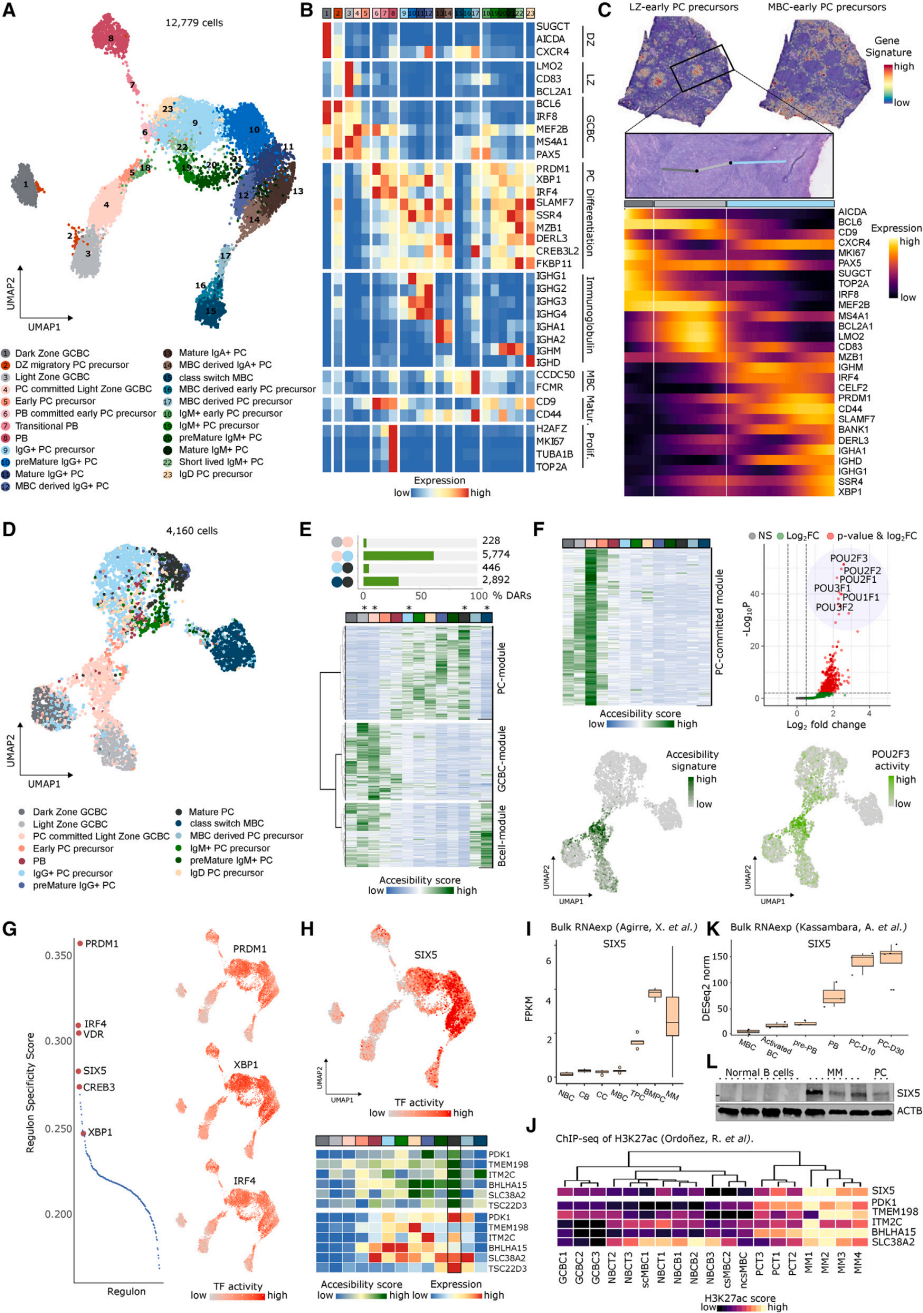
Plasma cell differentiation and cell identity regulation in human tonsils (A) UMAP of tonsillar plasma cells (PCs) colored and numbered by scRNA-seq clusters. (B) Heatmap showing scaled mean marker expression per PC subpopulation. (C) Top: transcriptomics-based tissue localization of LZ-derived early PC precursor (left) and MBC-derived early PC precursor (right), using the top 25 marker genes for each population. Middle: DZ (dark gray) to LZ (light gray) to subepithelial-PC-rich zone (light blue) trajectory on an H&E image from the highlighted area. Bottom: heatmap showing smoothed expression changes through the pre-defined trajectory. (D) UMAP of PC colored by scATAC-seq clusters. (E) Top: proportion of pairwise differentially accessible regions (DARs) between LZ, PC-committed, IgG PC precursor, mature PC, and csMBC. Bottom: clustered heatmap representation of the normalized accessibility score from the 9,340 DARs of the 3 main modules. (F) PC-committed module analysis. Left: heatmap showing normalized accessibility score of the 654 DARs and UMAP of their combined accessibility signature. Right: motif enrichment analysis of the 654 DARs (p cutoff: 0.001, FC cutoff: 0.5) and UMAP of top motif (POU2F3) activity (fold-enrichment: 2.57, p < 0.001). (G) Left: regulon specificity score for the PC subpopulation. Right: UMAP highlighting the activity (AUCell score) of PRDM1, XBP1, and IRF4 TFs. (H) Top: UMAP highlighting the activity (AUCell score) of SIX5. Bottom: heatmap showing scaled mean accessibility and gene expression for SIX5 targets. (I) Boxplot of fragments per kilobase per million fragments mapped (FPKM) values for SIX5 (NBC, naive B cell; CB, centroblast; CC, centrocyte; MBC, memory B cell; TPC, tonsillar plasma cell; BMPC, bone marrow plasma cell; MM, multiple myeloma). (J) Heatmap showing normalized mean H3K27ac signal for SIX5 and its targets (NBCT, tonsillar NBC; NBCB, NBC from peripheral blood; csMBC, class-switch MBC; ncsMBC, non-class switch MBC). (K) Boxplot of SIX5 expression during B cell maturation (BC, B cell; PB, plasmablast; PC-D10/30, *in vitro* generated PC at day 10/30). (L) Western blot showing SIX5 protein levels in normal B cells (CD19^+^ cells from three PBMC donors), multiple myeloma cell lines (XG6, XG21, and KMS11), and *in vitro* differentiated PC.

Overall, we could identify 20 different PC subpopulations, excluding DZ, LZ, and MBC cells, which were used to map B-to-PC transitions (Figure 5A). Most PCs originated from LZ-GCBCs, initially overexpressing key PC TFs (*PRDM1*, *IRF4*, and *XBP1*) followed by PC phenotypic markers (e.g., *SLAMF7* and *MZB1*; Figure 5B). We also identified a small PC precursor subpopulation, clustering with DZ- and LZ-GCBCs, which may represent precursor PCs migrating from the LZ to the DZ and leaving the GC at the DZ-T interface.^62^^,^^63^ We next identified the presence of precursor and transitional states leading to a clearly defined cluster of proliferative plasmablasts (PBs). These PBs showed signs of clonal expansion (Figure S5A) and a concomitant increased expression of proliferation and PC-related genes (Figure S5B). We also found that G2M phase cells expressed higher levels of these genes than S phase cells, supporting the concept that cell division is coupled to PC differentiation^64^^,^^65^ (Figure S5B). Following the proliferative stage, tonsillar PCs (TPCs) clustered according to Ig isotypes, maturation states, and the endoplasmic reticulum signature (Figures 5A, 5B, S5C, and S5D). In addition to PCs originated from GCBCs, we also characterized the putative transition from MBCs to PCs generated upon antigen reexposure (Figures 5A, 5B, and S10C).^66^

We then analyzed gene expression changes throughout a spatially defined trajectory from an intrafollicular zone to a subepithelial zone in different follicles from different tissue sections (Figures 5C, S10E, and S10F). Using subpopulation-specific markers, we observed the transition from the DZ to the LZ within the follicle, including initial expression of PC genes in the LZ and a strong PC signature increasing toward the subepithelial zone, where mature PCs locate (Figures 5C and S10F).^67^ Interestingly, a spatial expression correlation analysis revealed that the PC region contained distinct IgM/D and IgG/A areas (Figure S10G).

We next studied chromatin accessibility and transcriptional regulation during PC maturation and grouped the scRNA-seq subpopulations into 13 scATAC-derived clusters (Figure 5D). A pairwise differential accessibility analysis revealed highest differences between committed PCs and PC precursors, and from MBCs to mature PCs (Figure 5E), implying that cell fate transitions involve extensive chromatin programming. Clustering all differential accessible regions (DARs) identified three main modules of chromatin dynamics related to PC, GCBC, and B cell subpopulations (Figure 5E). Individually analyzing TF binding motifs in these modules revealed overrepresentation of TF motifs related to (1) the IRF family (*IRF8* and *IRF4*) and *MESP1* in the PC module; (2) *EBF1*, *PAX5*, *NFKB1*, *RELA/RELB*, and *MEF2C* in the GCBC module; and (3) *EBF1*, *PAX5*, *SPIB*, *SPI1*, and *ETV3/ETV6* in the B cell module (Table S5). Remarkably, we identified 654 DARs within the GCBC module showing increased chromatin accessibility in PC-committed LZ-GCBC. These DARs were strongly enriched in POU TF binding sites (i.e., *POU2F1*, *POU3F1*, and *POU2F2*), a TF family described to be crucial for PC differentiation toward an antibody secreting phenotype (Figure 5F).^68^^,^^69^^,^^70^

These epigenomic insights into PC differentiation were complemented with a gene regulatory network analysis (Table S4). Beyond observing IRF4, PRDM1, XPB1, VDR,^71^ and CREB3^72^ regulons, we identified SIX5,^73^^,^^74^ a TF not yet described in PCs that may be related to later stages of PC maturation (Figures 5G, 5H, and S5H; Table S4). The PC-specific expression of *SIX5* was confirmed using bulk RNA-seq^75^ and H3K27ac chromatin immunoprecipitation sequencing (ChIP-seq) data,^76^ as well as with scRNA-seq from peripheral blood^18^ and bone marrow^77^ (Figures 5I, 5J, S5I, and S5J). In line, the predicted target genes of SIX5 (*PDK1*, *TMEM198*, *ITM2C*, *BHLHA15/*MIST1, *SLC38A2*, and *TSC22D3*/GILZ) showed increased accessibility and selective expression in mature PCs (Figure 5H). We further validated these findings at the gene and protein expression using an *in vitro* PC differentiation model (Figures 5K and 5L).^78^ Finally, our analysis unveiled an upregulation of *SIX5* expression in multiple myeloma (MM), a PC-derived neoplasm,^79^ at both the gene and protein levels (Figures 5I and 5L). Additionally, we identified that regulatory regions of *SIX5* and its target genes were active in MM, as shown by increased H3K27ac (Figure 5J). Together, these results indicate SIX5 to be a new marker for mature PCs with a potential role in the regulation of MM tumorigenesis.

### Non-lymphoid tissue-resident and myeloid cell heterogeneity in human tonsils

In the epithelial compartment, we identified three clusters overlapping with the keratinocyte populations of the oral mucosa (Figures S6A–S6C).^80^ One of these clusters expressed *FDCSP* (FDCSP epithelium). FDCSP was first described in FDC and in “leukocyte-infiltrated tonsillar crypts,” although the specific population within the crypts remained unknown.^81^ Here, we provide evidence that FDSCP-expressing cells represent a specific subpopulation of the tonsillar epithelium.

We next classified cells of mesenchymal origin into FDCs, fibroblastic reticular cells (FRCs), and marginal reticular cells (MRCs; Figures S6D–S6F).^82^ MRCs expressed high levels of *COL1A1*, *COL1A2*, and *COL3A1* (among other collagens), which localized mostly at the interfollicular zone (Figure S6F). Intriguingly, MRCs expressed *PDGFRB*, which has been shown to be specific to perivascular precursor FDCs in mice (Figure S6E).^83^ Notably, we found three subsets of FDCs, including COL27A1^+^ FDCs and CD14^+^CD55^+^ FDCs. CD14^+^ FDCs are associated with poor prognosis in follicular lymphoma.^84^

**Figure 6:**
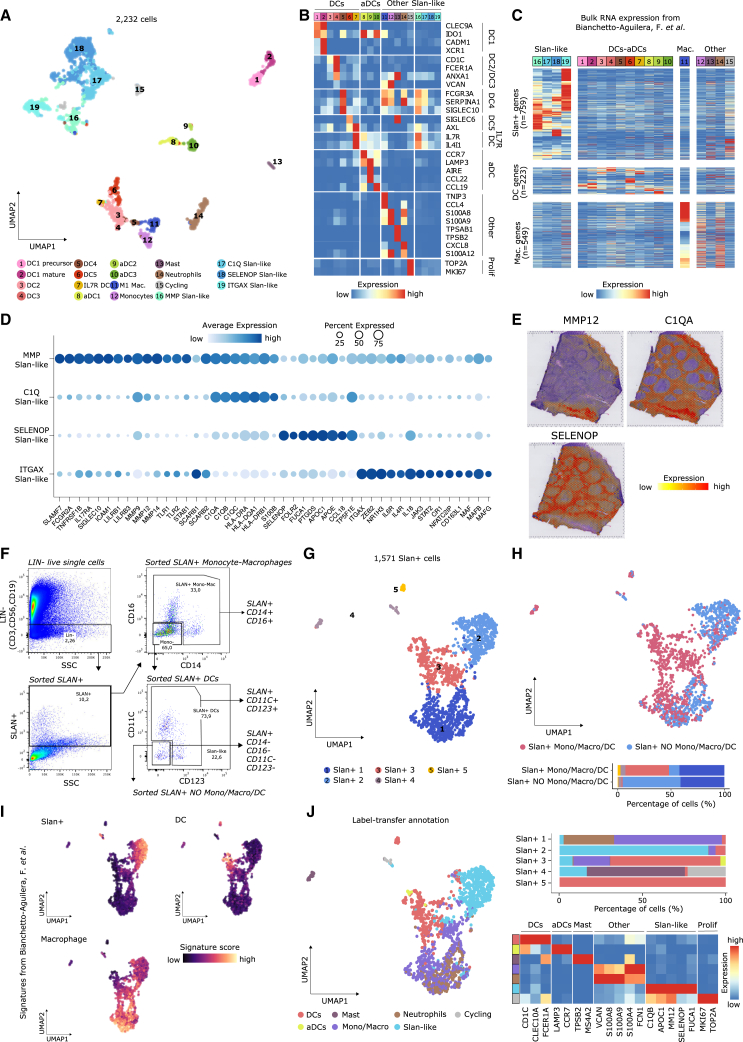
Myeloid cell heterogeneity in human tonsils (A) UMAP of tonsillar myeloid cells colored and numbered by scRNA-seq clusters. (B) Heatmap showing scaled mean marker expression per myeloid subpopulation. (C) Heatmap showing scaled mean expression of slan^+^, DC, and macrophage differentially expressed genes. (D) Dotplot showing expression of the top marker genes per slan-like subpopulation. (E) Denoised expression of genes identifying slan-like populations on an ST slide. (F) FACS isolation strategy of slan^+^ myeloid cells. (G) UMAP of sorted slan^+^ cells colored and numbered by scRNA-seq clusters. (H) Top: UMAP of sorted slan^+^ cells colored by sorting gate. Bottom: barplot showing cluster frequencies across sorting gates. (I) UMAPs colored by slan^+^, DC, and macrophage signatures (C). (J) Left: UMAP of sorted slan^+^ cells after label transfer from myeloid subpopulation (A). Right, top: barplot showing label-transferred subpopulation frequencies across the five slan^+^ clusters (G). Bottom, right: heatmap showing scaled mean marker expression per label-transferred subpopulation.

The transcriptional heterogeneity within the DC compartment was remarkably consistent with the one observed in blood^85^: (1) DC1, conventional DC1 (cDC1), divided into precursor and mature states on the basis of XCR1 expression and a proliferation signature^86^; (2) DC2 and DC3, corresponding to cDC2 and differing in their antigen-presenting capacity and inflammatory signatures, respectively; (3) DC4, putatively derived from non-classical monocytes (*FCGR3A/CD16*); and (4) DC5 expressing *AXL* and *SIGLEC6* (AS), the hallmark markers of AS DCs (Figures 6A, 6B, and S6G–S6I). Intriguingly, we identified a previously uncharacterized cluster that expressed *AXL* and *IL-7R* but not *SIGLEC* and additional marker genes, such as *IL1RN*, *IL1B*, and *CD83* (Figures 6B and S6G). We also found three migratory CCR7^+^ DC populations (activated DC [aDC]) previously characterized in the thymus (Figures 6A, 6B, S6G, and S6I),^4^^,^^87^ although we could not link them to their DC counterpart. Noteworthy, aDC2 expressed shallow levels of autoimmune regulator (*AIRE*), which has a role in peripheral tolerance (Figures 6B and S6I).^88^^,^^89^^,^^90^ Finally, we annotated four clusters as monocytes, M1 macrophages, mast cells, and neutrophils (Figures 6A and 6B).

Tonsil 6-sulfo LacNAc^+^ (slan^+^) cells derive from non-classical monocytes and are distinct from cDC2 and macrophages.^91^ Quantifying the slan^+^ signature,^91^ we identified four slan-like cell subpopulations, representing the most prevalent myeloid cell type in tonsils (Figures 6A–6C and S6J). These slan-like cells included the following: (1) MMP cells expressing metalloproteinases and Toll-like receptors, (2) C1Q cells expressing complement members and class II MHC genes, (3) SELENOP cells expressing apolipoproteins and fucosidases, and (4) ITGAX cells expressing scavenger receptors (Figure 6D). Because SELENOP expression was vastly specific to SELENOP slan-like cells across the 121 cell types and states of the tonsil atlas (Figure S6K), we used it as a proxy of their spatial location. Noteworthy, *SELENOP* was mostly expressed at the interfollicular/T cell zone, while it was absent in the epithelium and follicles (Figure 6E). *MMP12* was expressed subepithelial, while *C1QA* expression localized both subepithelial and at the interfollicular zones (Figure 6E). IL-7R DCs also expressed *MMP12* and *C1QA* (Figure S6K), however their low prevalence (Figures 6A and S6J) suggests the main source of *MMP12* and *C1QA* to be the slan-like populations.

To validate the annotation of slan^+^ cells and to further distinguish their identity from cDC and macrophage populations, we combined slan^+^ fluorescence-activated cell sorting (FACS)-enrichment with subsequent scRNA-seq (Figure 6F). In detail, we isolated slan^+^ myeloid cells, further classified into monocytes/macrophages (SLAN^+^CD14^+^ or SLAN^+^CD16^+^), DC (SLAN^+^CD11C^+^ or SLAN^+^CD123^+^), and slan-like cells (SLAN^+^CD14^−^CD16^−^CD11C^−^CD123^−^). We observed three main clusters of slan^+^ myeloid cells, uniquely enriched for transcriptomic signatures of DCs, macrophages, and slan-like cells and confirming the substantial heterogeneity within the slan^+^ compartment (Figures 6G–6I). Next, we mapped the sorted cells onto the tonsil atlas reference, resulting in a clear separation of the three main myeloid populations, an annotation further supported by expression of respective marker genes in label-transferred cells (Figure 6J). Of note, the sorted slan-like cells (SLAN^+^CD14^−^CD16^−^CD11C^−^CD123^−^) mapped to the slan-like clusters, confirming that they correspond to the same cell type (Figure 6J). Taken together, we have discovered and validated four previously uncharacterized subtypes of slan-like cells that have different spatial locations and could control different aspects of immune responses.

### The tonsil as an HCA resource

**Figure 7:**
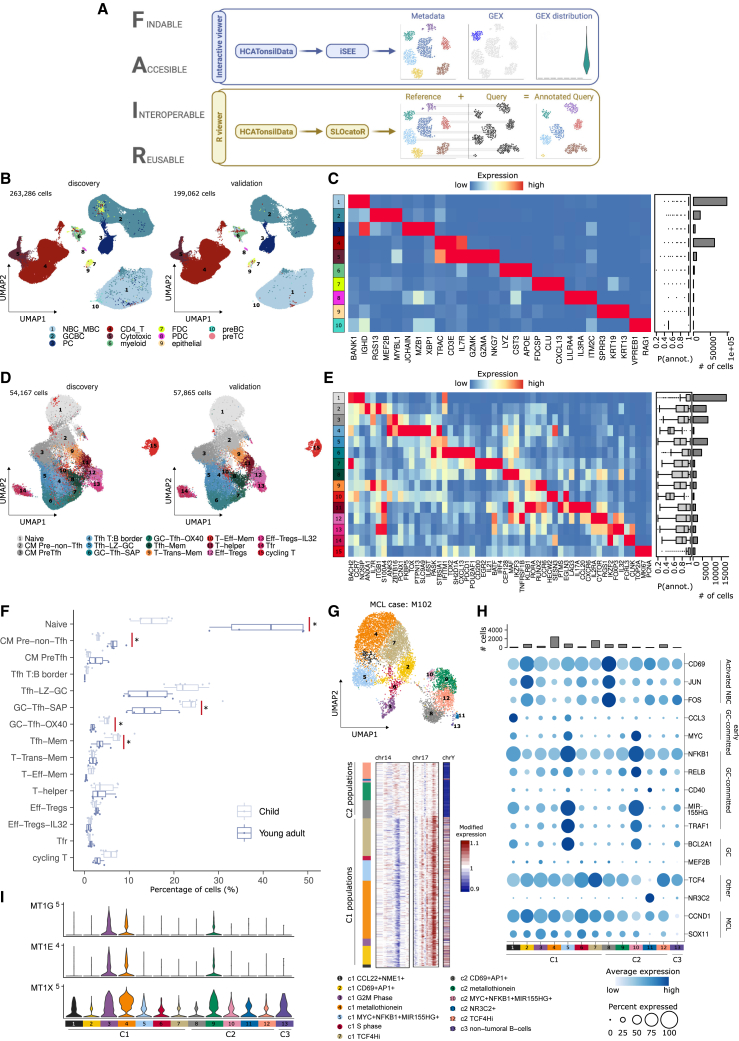
Dissemination and application of the tonsil atlas (A) Schematic representation showing the computational framework to access and reuse the tonsil atlas dataset. (B and D) UMAP of all cells (B) and CD4 T cells (D) of the reference and query (validation cohort) annotated with SLOcatoR. (C and E) Heatmap showing scaled mean marker expression of level 1 clusters (C) and CD4 T subclusters (E) for the validation cohort. Boxplots represent the annotation confidence for each cluster, and barplots represent the number of cells for that cluster in the validation cohort. (F) Boxplot showing the percentage of CD4 T subclusters across child and young adult subgroups (inclusion criteria: scRNA-seq, fresh samples, tonsillitis). Asterisks indicate significant changes (scCODA, false discovery rate [FDR] = 0.1). (G) Top: UMAP of MCL cells from case M102 colored and numbered by scRNA-seq clusters. Bottom: inferCNV result using c3 non-tumoral B cells as reference (showing chromosomes with large copy-number changes). (H) Top: barplot showing total number of cells per M102 scRNA-seq clusters. Bottom: dotplot showing the average expression of normal and neoplastic B cell markers across M102 scRNA-seq clusters. (I) Violinplot showing the expression of metallothionein genes across annotated clusters of case M102.

To make our data findable, accessible, interoperable, and reusable (FAIR),^92^ we developed HCATonsilData, an R/BioConductor data package that provides modular and programmatic access to the tonsil atlas dataset. The users can access SingleCellExperiment^93^ objects, easily convertible to AnnData^94^ objects via zellkonverter,^95^ ensuring interoperability. We also provide a detailed glossary (Data S1) listing evidence for the annotation of all 121 cell types and states of this tonsil atlas, with interactive exploration through iSEE instances (Figure 7A).^96^

To promote reproducible research practices and facilitate the reuse of our code, we developed SLOcatoR: an R package that allows users to annotate datasets from SLO using the tonsil atlas reference (Figure 7A; see STAR Methods). We applied SLOcatoR to annotate cells from the validation cohort to (1) confirm the presence, annotation, and markers of cell types, (2) extend the atlas through an integrated validation dataset, and (3) chart compositional changes in the tonsil during aging. We additionally included our reference in Azimuth, which now allows for interactive exploration and annotation of cell types from SLO.

The integrated tonsil atlas represented >462,000 single-cell transcriptomes, allowing for label transfer of the 121 reference atlas clusters (Figures 7B–7E and S7). The label transfer was validated 3-fold: (1) preservation of cell neighborhoods, (2) conservation of *bona fide* marker genes, and (3) annotation confidence (see STAR Methods). Overall, we validated clusters with a high annotation confidence (mean 0.825) and conserving main marker genes, exemplified by the validation of all four slan-like subsets (Figures 7D, 7E, and S7). As observed previously,^97^ infrequent cell types (e.g., preB and aDC2) and transient cell types (e.g., T:B border cells) had lower annotation confidences. Similarly, clusters between major populations were challenging to annotate and require further validation (e.g., memory-derived PC, Data S1).

In the T cell compartment, we found a significant increase^98^ in the relative abundance of naive and CM pre-non-Tfh cells in young adults, while GC-Tfh-SAP, GC-Tfh-OX40, and Tfh-Mem populations were significantly decreased (Figure 7F), supporting the age-related decrease in follicles and GC-specific cells.

Finally, we profiled two tonsillar conventional MCL samples. In both patients, we observed a major cluster with chromosome Y loss, a common feature of MCL and other cancers,^99^^,^^100^ allowing for the classification of neoplastic cells into chrY^+^ and chrY^−^ (c1/c2; Figures 7G and S7K–S7N). We identified additional subclonal genetic alterations accompanying the chromosome Y loss (Figures 7G, S7O, and S7P).^101^ Analyzing MCL data in the context of our tonsil atlas, MCL cell states appeared to be reminiscent of normal B cell states, with markers detected in a maturation interval from activated NBCs to GC-committed cells (Figures 7H and S7N). We also observed two clusters with increased metallothionein gene expression, a cell state recently recognized as a recurrent neoplastic program in cancer (Figure 7I).^102^ Together, these analyses suggest that MCL cells are not frozen in a particular maturation stage, but they retain a certain differentiation potential of normal B cells. Thus, our tonsil atlas could be informative about the normal counterparts of tumor cell states and may pinpoint additional disease-driving mechanisms.

## Discussion

We provide a detailed taxonomy of cells in the human tonsil. In addition to the annotation of cell types using single-cell transcriptomics, the multimodal nature of our atlas allowed for the fine-grained interrogation of subtle cell states and their driving mechanisms through gene regulatory or spatial determinants. The high number of profiled cells, as compared with previous single-cell tonsil studies,^12^ allowed for the identification of 121 cell types and states, including rare ones, such as preB and preT cells. We identified cell types and states, including four subtypes of slan-like myeloid cells, precursor populations of Tfh and non-Tfh CM CD4^+^ T cells, two terminally differentiated subtypes of Tfh cells, and Treg heterogeneity. We also described the stepwise maturation stages associated with NBC activation toward the GC, the GC dynamics between the DZ and LZ, as well as multiple states of PC differentiation with unprecedented resolution. Despite the depth of our atlas, we recognize that a cell annotation consensus is a community effort, especially for newly reported subtypes, which we facilitate through the accessibility of data, analysis code, and a thoroughly assembled glossary (Data S1). To broaden the utility, we designed HCATonsilData to ease data integration and community-driven annotation.

The multimodal study design further enabled the interrogation of regulatory circuits driving cell-type specialization. We illustrate that the *BCL6* distal enhancer described in GCBCs^31^ is also active in Tfh. We further disentangle the TF hierarchy associated with the DZ entry, which seems to be shared in LZ cells reentering the DZ as well as activated NBCs entering the DZ for the first time. Charting the regulatory landscape in PCs, we discovered SIX5 as a new potential TF associated with PC maturation.

Beyond providing an atlas as a resource and reference map of the human tonsil, we provide a proof of concept for its utility to determine alterations observed during aging and in diseases such as MCL. Despite its clonal origin, MCL cells generate an intraclonal transcriptional ecosystem with different subclusters related to B cell maturation, a phenomenon that has also been observed in other B cell tumors, such as follicular lymphoma.^103^ Thus, cells from different B cell tumors do not seem to be frozen in a single maturation state but rather display phenotypic plasticity constrained to particular windows of normal B cell maturation.

### Limitations of the study

Additional functional studies are needed to decipher the role of multiple cell types described in this atlas. For instance, we foresee that further functional characterization of the slan-like compartment will unravel the specialized functions of the four myeloid subsets we described here. Because the markers of our slan-like clusters partially overlap with macrophage states reported in other efforts, future studies will unequivocally clarify whether slan-like represents a distinct myeloid cell type. Also, further experimental evidence is needed to disentangle the role of the SIX5 TF in PC lineage commitment. While current atlases of healthy human organs and tissues focus on the analysis of mostly transcriptional data, the presented atlas integrated five modalities, including spatially resolved transcriptional profiling. However, currently available ST technologies for transcriptome-wide profiling do not provide *bona fide* single-cell resolution and require capture site deconvolution to predict cell-type location by integrating single-cell and ST datasets, or analysis must be limited to signature and marker gene visualization. New technologies^104^^,^^105^ will soon overcome such limitation, once becoming broadly accessible, and one can foresee future cell atlases to perform single-cell-resolved phenotyping directly from tissue section, avoiding tissue dissociation and related technical artifacts that could bias cell composition or gene expression profiles.

## STAR★Methods

### Key resources table

**Table:** 

REAGENT or RESOURCE	SOURCE	IDENTIFIER
**Chemicals, peptides, and recombinant proteins**		

1X Phosphate-Buffered Saline	Thermo Fisher	Cat#20012-019
MACS BSA Stock Solution	Miltenyi Biotec	Cat#130-091-376
Nuclease free water	Ambion	Cat#AM9939
Hibernate-A medium	Gibco	Cat#A1247501
RNAse Inhibitor	Roche	Cat#3335402001
Trypan blue	Fischer Scientific	Cat#15250-061
Digitonin 5%	Fischer Scientific	Cat#10636033
NaCl 5 M	Ambion	Cat#AM9759
MgCl2 1M	Ambion	Cat#AM9530G
Nonidet P40	Sigm-Aldrich	Cat#74385
Tween-20	Thermo Fisher	Cat#85114
Tris-HCl 1M pH 7.5	Thermo Fisher	Cat#15567027

**Critical commercial assays**		

Chromium Next GEM Single Cell 3’ GEM, Library & Gel Bead Kit v3.1	10x Genomics	Cat#1000121
Chromium Next GEM Single Cell ATAC Library & Gel Bead Kit v1.1	10x Genomics	Cat#1000175
Chromium Next GEM Single Cell 5’ Library & Gel Bead Kit v1.1	10x Genomics	Cat#1000165
Chromium Single Cell V(D)J Enrichment Kit, Human T Cell	10x Genomics	Cat#1000005
Chromium Single Cell V(D)J Enrichment Kit, Human B Cell	10x Genomics	Cat#1000016
Visium Spatial Tissue Optimization Slide & Reagent Kit	10x Genomics	Cat#1000193
Visium Spatial Gene Expression Slide & Reagent Kit	10x Genomics	Cat#1000184
Agilent High Sensitivity DNA Kit	Agilent	Cat#5067-4626
Agilent RNA 6000 Pico Kit	Agilent	Cat#5067-1513
RNeasy Plus Micro kit	Qiagen	Cat#74034
AMPure XP Bead-Based Reagent	Beckman Coulter	Cat#A63881

**Software and algorithms**		

Cellranger-atac v1.2	CellRanger ATAC (10X Genomics)	https://support.10xgenomics.com/single-cell-atac/software/overview/welcome
Cellranger v4.0.0	CellRanger (10X Genomics)	https://support.10xgenomics.com/single-cell-gene-expression/software/overview/welcome
Cellranger-arc v1.0	CellRanger ARC (10X Genomics)	https://support.10xgenomics.com/single-cell-multiome-atac-gex/software/overview/welcome
Cellranger-multi v6.0.1	CellRanger multi (10X Genomics)	https://support.10xgenomics.com/single-cell-vdj/software/pipelines/latest/using/multi
Spaceranger v1.1.0	10X Genomics	https://support.10xgenomics.com/spatial-gene-expression/software/overview/welcome
Seurat v3.2.0 and v4.1.0	Hao et al.^18^	https://satijalab.org/seurat/
Signac v1.1.0	Stuart et al.^106^	https://satijalab.org/signac/
Harmony v1.0	Korsunsky et al.^107^	https://github.com/immunogenomics/harmony
clusterProfiler v4.3.4	Wu et al.^108^	https://yulab-smu.top/biomedical-knowledge-mining-book/
UCell v1.2.0	Andreatta and Carmona^109^	https://bioconductor.org/packages/release/bioc/html/UCell.html
MACS2 v2.2.7.1	Zhang et al.^110^	https://github.com/macs3-project/MACS
ChromVar v1.1.0	Schep et al.^111^	http://www.bioconductor.org/packages/release/bioc/html/chromVAR.html
Cicero v1.3.4	Pliner et al.^112^	http://cole-trapnell-lab.github.io/cicero-release
pySCENIC v0.10.3	Van de Sande et al.^24^	https://pyscenic.readthedocs.io/en/latest/
SCENIC+	Bravo González-Blas et al.^25^	https://scenicplus.readthedocs.io/
pycisTopic	Bravo González-Blas et al.^25^	https://github.com/aertslab/pycisTopic
pycistarget	Bravo González-Blas et al.^25^	https://github.com/aertslab/pycistarget
Vireo v0.5.0	Huang et al.^113^	https://github.com/single-cell-genetics/vireo
cellsnp-lite v1.2.0	Huang and Huang^114^	https://github.com/single-cell-genetics/cellsnp-lite
Scirpy v0.7.0	Sturm et al.^115^	https://github.com/scverse/scirpy
SPOTlight v0.1.7	Elosua-Bayes et al.^116^	https://www.bioconductor.org/packages/release/bioc/html/SPOTlight.html
Rmagic v2.0.3	van Dijk et al.^117^	https://github.com/KrishnaswamyLab/MAGIC
SPATA2 v0.1.0	Kueckelhaus et al.^118^	https://github.com/theMILOlab/SPATA2
LISI v1.0	Korsunsky et al.^107^	https://github.com/immunogenomics/LISI
Scrublet v0.2.1	Wolock et al.^119^	https://github.com/swolock/scrublet
ChipSeeker v1.34.1	Wang et al.^120^	https://bioconductor.org/packages/release/bioc/html/ChIPseeker.html
scCODA v0.1.9	Büttner et al.^98^	https://pypi.org/project/scCODA/
Infercnv	Tirosh et al.^101^	https://github.com/broadinstitute/inferCNV/wiki
ComplexHeatmap v2.14.0	Gu^121^	https://jokergoo.github.io/ComplexHeatmap-reference/book/
R	R core	https://www.r-project.org
Python	Python Software Foundation	https://www.python.org
Custom Shiny App to annotate clusters	This paper	https://singlecellgenomics-cnag-crg.shinyapps.io/Annotation/
Custom Shiny App to annotate histology slides	This paper	https://github.com/Single-Cell-Genomics-Group-CNAG-CRG/shiny-pathology
HCATonsilData	This paper	https://bioconductor.org/packages/release/data/experiment/html/HCATonsilData.html
SLOcatoR	This paper	https://github.com/massonix/SLOcatoR
iSee instance	This paper	http://shiny.imbei.uni-mainz.de:3838/iSEE_TonsilDataAtlas/
Azimuth app and reference	This paper	https://azimuth.hubmapconsortium.org/references/#Human%20-%20Tonsil%20v2
Code and vignettes	This paper	https://github.com/Single-Cell-Genomics-Group-CNAG-CRG/TonsilAtlas
Glossary	This paper	Data S1 and https://doi.org/10.6084/m9.figshare.24885063

**Other**		

Scenic TF Database	Van de Sande et al.^24^	https://github.com/aertslab/SCENICprotocol/blob/master/example/allTFs_hg38.txt
CisTarget databases Hg38__refseq-r80__500bp_up_and_100bp_down_tss.mc9nr.feather motifs-v9-nr.hgnc-m0.001-o0.0.tbl	Herrmann et al.^122^ and Imrichová et al.^123^	https://resources.aertslab.org/cistarget/
JASPAR2020 v0.99.10	Fornes et al.^124^ and Tan and Lenhard^125^	https://bioconductor.org/packages/release/data/annotation/html/JASPAR2020.html
chromVARmotifs v0.2.0	Schep et al.^111^	https://github.com/GreenleafLab/chromVARmotifs

**Deposited data**		

Fastq files	This paper	ArrayExpress: E-MTAB-13687
Outputs CellRanger (expression and accessibility matrices)	This paper	https://doi.org/10.5281/zenodo.10373041
Seurat objects	This paper	https://doi.org/10.5281/zenodo.8373756

### Resource availability

#### Lead contact

Requests for further information or access to data should be directed to Holger Heyn (holger.heyn@cnag.eu).

#### Materials availability

This study did not generate new unique reagents.

#### Data and code availability

The data has been deposited in five levels of organization, from raw to processed data:

Level 1: raw data. All fastq files for all data modalities have been deposited at ArrayExpress under accession id E-MTAB-13687.
Level 2: matrices. All data modalities correspond to different technologies from 10X Genomics. As such, they were mapped with different flavors of CellRanger (CR). The most important files in the “outs” folder of every CR run (including all matrices) have been deposited in Zenodo.
Level 3: Seurat Objects. All data was analyzed within the Seurat ecosystem. We have archived in Zenodo all Seurat Objects that contain the raw and processed counts, dimensionality reductions (PCA, Harmony, UMAP), and metadata needed to reproduce all figures from this manuscript.
Level 4: to allow for programmatic and modular access to the whole tonsil atlas dataset, we developed HCATonsilData, available on BioConductor. HCATonsilData provides a vignette which documents how to navigate and understand the data. It also provides access to the glossary to traceback all annotations in the atlas. In addition, we will periodically update the annotations as we refine it with suggestions from the community.
Level 5: interactive mode. Our tonsil atlas has been included as a reference in Azimuth, which allows interactive exploration of cell type markers on the web.

All code related with this publication is available on GitHub:

- Scripts and notebooks to reproduce all analysis: https://github.com/Single-Cell-Genomics-Group-CNAG-CRG/TonsilAtlas. Most analysis notebooks have a companion html report that has all the plots that motivate the thresholds and parameters used in these analyses.
- SLOcatoR package: https://github.com/massonix/SLOcatoR.
- Shiny app used to annotate cells: https://singlecellgenomics-cnag-crg.shinyapps.io/Annotation/.
- Shiny app used to annotate histology slides: https://github.com/Single-Cell-Genomics-Group-CNAG-CRG/shiny-pathology.
- Code to generate iSEE instances: https://github.com/iSEE/iSEE_instances/tree/master/iSEE_HCATonsilData.
- HCATonsilData package: https://github.com/massonix/HCATonsilData/.

### Experimental model and subject details

#### Sample collection and processing (Hospital Clinic and CIMA)

We divided the sample collection into two cohorts: the discovery cohort (which we used to cluster cells, annotate cell types and identify bona-fide markers), and the validation cohort (which we used to validate cell types and markers). For the discovery cohort, we obtained ten tonsil samples from donors from three different age groups, *i.e.*, six children (age 3-5; 3 males and 3 females; recurrent tonsillitis), three young adults (age 26-35; all male; sleep apnea) and one old adult (age 65; male, tonsil removed during surgery for benign pharyngeal squamous papillomatosis; Figure 1A and Table S1). Out of the ten tonsils, eight were obtained in Clinica Universidad de Navarra (Pamplona, Spain; all kids and 2 young adults) and two at Hospital Clinic Barcelona (Barcelona, Spain; one young and one old adult). All donors or legal guardians gave informed consent for their participation in this study, which was approved by the clinical research ethics committee of Clínica Universidad de Navarra and by the clinical research ethics committee of the Hospital Clinic of Barcelona (HCB/2018/0992). For the validation cohort, we obtained four tonsils from young adults undergoing elective tonsillectomies for recurrent tonsillitis at Newcastle Upon Tyne Hospitals NHS Foundation Trust (United Kingdom). All donors provided written informed consent for study participation, which was approved by the West of Scotland Research Ethics Service (22/WS/0126). Furthermore, the validation cohort included one young adult (age 25; female; recurrent tonsillitis) and two old adults (age 56, 63; both males; sleep apnea and tonsil removed during surgery for superficial squamous carcinoma of the laryngeal vocal cord) sampled at Hospital Clinic after giving their informed consents.

All tonsil samples from the discovery and validation cohorts were reviewed at the Hematopathology Unit of Hospital Clinic of Barcelona and showed reactive follicular hyperplasia with several degrees of GC expansions. No atypical cells in the epithelium, stroma or lymphoid compartments were seen in the cases including the two tonsils (BCLL-2-T and BCLL-24-T; Table S1) that were extracted during a surgery intervention for benign pharyngeal squamous papillomatosis and superficial squamous carcinoma of vocal cord, respectively.

Tonsil tissues were split into three parts that were processed as follows: (1) first part was paraffin embedded to create FFPE blocks, according to standard pathology protocols; (2) second part was snap frozen to obtain OCT blocks, according to standard protocols, and (3) the remaining third part was processed to obtain a single-cell suspension, following the steps described below. Tonsils were first disaggregated by extensive manual mincing and filtered by 70 μm (samples from CIMA and Hospital Clinic) or 100 μm (samples from Newcastle Upone Tyne Hospitals) nylon strainer. In case non-disaggregated parts of tissue were still present, the samples were further dissociated by gentle MACS Dissociator (program tumor 04.01). The number and viability of cells was evaluated. All steps were performed at 4°C or on ice. Cells were either processed directly for single-cell sequencing (scRNA-seq, scATAC-seq, CITE-seq, or Multiome) or cryopreserved for later use.

For the final part of the study, cryopreserved cells from tonsil samples of two MCL patients were used (age 64 and 80, both male). Informed consents were obtained according to the Institutional Review Board of the Hospital Clínic of Barcelona following the International Cancer Genome Consortium guidelines. MCL samples were obtained from cryopreserved dissociated cells from tonsils, from the ICGC case collection of Hospital Clinic, Barcelona. After thawing in culture medium supplemented with 20% FBS, the CD19 positive B cell fraction and CD19 negative non-B cell fraction were isolated by magnetic cell separation using CD19-MicroBeads MACS separation system protocol (Miltenyi Biotec, Auburn, CA). Separation steps were performed at 4°C. Both fractions were directly processed for Multiome library preparation and sequenced separately.

### Method details

#### 3’ scRNA-seq and Cell hashing (Hospital Clinic and CIMA)

Freshly isolated cells from tonsils were subjected to a Cell Hashing^126^ protocol before proceeding to scRNA-seq. Cell hashing was performed following manufacturer’s instructions (Cell hashing and Single Cell Proteogenomics Protocol Using TotalSeq™ Antibodies; BioLegend). Cells were counted with a TC20™ Automated Cell Counter (Bio-Rad Laboratories, S.A), and 50,000 unlabeled cells were saved in a separate tube before proceeding with the cell hashing protocol. Each sample was split into seven aliquots with equal numbers of cells. Briefly, each aliquot was resuspended in Cell Staining Buffer (BioLegend), incubated for 10 min at 4°C with Human TruStain FcX™ Fc Blocking reagent (BioLegend). To each aliquot, a specific TotalSeq-A antibody-oligo conjugate (Table S6) was added and incubated on ice for 30 min. Cells were then washed three times with cold PBS+0.05% BSA (ThermoFisher) and centrifuged for 5 min at 500 rcf at 4°C. Finally, cells were resuspended in an appropriate volume of PBS+0.05% BSA to obtain a final cell concentration >1000 cells/μl, suitable for scRNA-seq. Assuming a 50% loss of cells in all tubes, an equal volume of hashed cell suspension from each of the seven aliquots was mixed and filtered with a 40 μm strainer. Cell concentration was verified with a TC20™ Automated Cell Counter (Bio-Rad Laboratories, S.A) upon cell staining with Trypan Blue.

Cells were partitioned into Gel Beads-in-emulsion (GEMs) by using the Chromium Controller system (10X Genomics). Each sample was loaded into two channels with a target recovery of 20,000 cells per channel, for a total final recovery of 40,000 cells per sample. To assess the potential effects of cell hashing on gene expression and cell type composition, we additionally added non-hashed cells as a control (TR=5,000 cells). cDNA sequencing libraries were prepared using the Next GEM Single Cell 3’ Reagent Kits v3.1 (10X Genomics, PN-1000121), with some adaptations for Cell hashing, as indicated in TotalSeq™-A antibodies and Cell Hashing with 10X Single Cell 3' Reagent Kit v3 3.1 protocol by BioLegend. Briefly, 1 μl of 0.2 μM hashtag oligonucleotides (HTO) primer (Integrated DNA Technologies, IDT) was added to the cDNA amplification reaction to amplify the HTO together with the full-length cDNAs. A SPRI selection clean up was done to separate mRNA-derived cDNA (>300 bp) from antibody-oligo-derived cDNA (<180 bp), as described in the above-mentioned protocol form BioLegend. Gene Expression (GEX) libraries were prepared following 10X Genomics single-cell 3’ mRNA kit protocol, while HTO DNAs were indexed by PCR as follows. 5 μl of purified HTO DNA were mixed with 2.5 μl of 10 μM Illumina TruSeq D70X_s primer (IDT) carrying a different i7 index for each sample (Table S6), 2.5 μl of SI primer from 10X single-cell 3’ mRNA kit, 50 μl of 2 X KAPA HiFi PCR Master Mix (KAPA Biosystem) and 40 μl of nuclease-free water. The reaction was carried out using the following thermal cycling conditions: 98°C for 2 min (initial denaturation), 12 cycles of 98°C for 20 seconds, 64°C for 30 seconds, 72°C for 20 seconds, and a final extension at 72°C for 5 min. The HTO libraries were purified by adding 1.2 X SPRI select reagent to the PCR reaction, incubating 5 min at room temperature (RT) and removing the supernatant after capturing the beads with a magnet. Samples were washed two times with 80% ethanol and elution was performed by adding 40.5 μl of nuclease-free water to the beads.

Size distribution and concentration of full-length cDNA and HTO libraries were verified on an Agilent Bioanalyzer High Sensitivity chip (Agilent Technologies). Finally, sequencing of HTO and GEX libraries was carried out on a NovaSeq 6000 sequencer (Illumina) using the following sequencing conditions: 28 bp (Read 1) + 8 bp (i7 index) + 0 bp (i5 index) + 89 bp (Read 2), to obtain approximately 2,000 and >20,000 paired-end reads per HTO and cell, respectively.

#### 3’ scRNA-seq (Newcastle Upon Tyne Hospitals)

Fresh cells obtained by FACS sorting were counted, and resuspended in the appropriate volume of PBS to obtain a final cell concentration of 1000 cells/μl for scRNA-seq. Each fresh sample was loaded into two channels of a chromium controller system (10X Genomics) for partitioning into gel beads-in-emulsion (GEMs), with a target recovery of 10,000 cells per channel and 20,000 cells per sample. cDNA libraries were prepared using the Next GEM Single Cell 3’ Reagent Kits v3.1 (10X Genomics, PN-1000121). Gene expression (GEX) libraries were prepared according to the 10X Genomics single-cell 3’ mRNA kit protocol. Size distribution and concentration of full-length cDNA libraries were quality controlled using a 4200 TapeStation System (Aligent Technologies). GEX libraries were sequenced using a NovaSeq 6000 (Illumina) using the following sequencing conditions: 28 bp (Read 1) + 10 bp (i7 index) + 10 (i5 index) + 90 bp (Read 2) to obtain approximately 25,000 paired-end reads per cell.

#### scATAC-seq

Cryopreserved samples were rapidly thawed in a 37°C water bath and transferred to a 15 ml Falcon using a 1000 μl wide bore tip. Next, 1 ml of 37°C pre-warmed Hibernate-A media supplemented with 10% FBS (Thermo Fisher Scientific) was added dropwise while gently swirling the sample. After 1 min RT incubation, 2 ml of pre-warmed media were added as mentioned before. Samples were again incubated at RT for 1 min and then additional media was added to bring the volume to 15 ml. Samples were centrifuged at 500 x g for 5 min at RT. Supernatant was removed and pellets resuspended in 10 ml of 1X PBS (Thermo Fisher Scientific) supplemented with 1% BSA. Samples were filtered with a 40 μm cell strainer to remove clumps. Cell number and viability were verified with a TC20™ Automated Cell Counter (Bio-Rad). Dead cells were removed by FACS sorting DAPI negative cells using a FACSAria™ Fusion Flow Cytometer (BD Biosciences). To determine potential biases introduced by cell sorting, unsorted cells from 4 out of 10 samples were used for scATAC-seq analysis and compared with corresponding sorted samples.

Nuclei isolation was performed following the “Nuclei Isolation for Single Cell ATAC Sequencing” demonstrated protocol (10X Genomics; CG000169) starting from 1 million cells per sample and incubating on ice for 3 min for cell lysis. Based on the starting number of cells and assuming a 50% loss during the procedure, nuclei were resuspended into the appropriate volume of chilled Diluted Nuclei Buffer (10x Genomics) to achieve a nuclei concentration of 925-2300 nuclei/μl, suitable for a Target Nuclei Recovery of 5,000 nuclei per sample. The resulting nuclei concentration was determined by manual counting using a Neubauer chamber upon staining with Trypan Blue.

scATAC-seq libraries were prepared according to the Chromium Single Cell ATAC Reagent Kits v1.1 User Guide (10x Genomics; CG000209 Rev D). Transposed nuclei were partitioned into GEMs by using the Chromium Controller with Chip H for a target recovery of 5000 nuclei per sample. For samples used to assess potential artifacts due to FACS sorting, nuclei obtained from sorted and unsorted cells were loaded on separate channels of the same chip and parallelly processed for library preparation. After linear amplification, the resulting DNA was purified by sequential Dynabeads and SPRIselect reagent beads clean-ups. Libraries were indexed by PCR using the Single Index Kit N Set A (10X Genomics, PN-1000212) applying 10 cycles of amplification. Sequencing libraries were subjected to a final bead clean-up SPRIselect reagent and quantified on an Agilent Bioanalyzer High Sensitivity chip (Agilent Technologies). Finally, libraries were loaded on an Illumina NovaSeq 6000 with the following sequencing conditions: 50 bp (Read 1N) + 8 bp (i7 Index) + 16 bp (i5 Index) + 49 bp (Read 2N), aiming at a sequencing depth of >25,000 reads/nucleus.

#### Single cell RNA and chromatin accessibility profiling

Cryopreserved cells were thawed and cleaned from dead cells as described before (see scATAC-seq). Nuclei isolation was performed following the “Nuclei Isolation for Single Cell Multiome ATAC + Gene Expression Sequencing'' demonstrated protocol (10x Genomics; CG000365) starting from 0.5-1 M cells per sample and incubating on ice during 3 min for cell lysis. Nuclei were resuspended into the appropriate volume of chilled Diluted Nuclei Buffer (10X Genomics) to achieve a nuclei concentration of 925-2,300 nuclei/μl, suitable for a Target Nuclei Recovery of 7,000 per sample. The resulting nuclei concentration was determined by manual counting using a Neubauer chamber upon staining with Trypan Blue.

GEX and ATAC-seq libraries were prepared following the Chromium Next GEM Single Cell Multiome ATAC + Gene Expression User Guide (10X Genomics; CG000338). Transposed nuclei were partitioned into GEMs by using the Chromium Controller with Chip J aiming at a target recovery of 7,000 nuclei per sample. After GEMs incubation for mRNA reverse transcription and transposed DNA barcoding, the resulting cDNA and barcoded gDNA were purified and pre-amplified during 7 cycles, following the 10X Genomics protocol. After a clean-up, 35 μl of the pre-amplified cDNA were amplified with 7 additional PCR cycles. The resulting cDNA was quantified on an Agilent Bioanalyzer High Sensitivity chip (Agilent Technologies) and 100 ng were used for library preparation. GEX libraries were indexed with 13 cycles of amplification using the Dual Index Plate TT Set A (10X Genomics; PN-3000431). In parallel, 40 μl of the pre-amplified DNA were indexed with 7 cycles of amplification using the Sample Index N Set A (10X Genomics; PN 3000427). Size distribution and concentration of full-length GEX and ATAC-seq libraries were verified on an Agilent Bioanalyzer High Sensitivity chip (Agilent Technologies). Finally, sequencing of GEX libraries was carried out on a NovaSeq 6000 sequencer (Illumina) using the following sequencing conditions: 28 bp (Read 1) + 8 bp (i7 index) + 0 bp (i5 index) + 89 bp (Read 2), to obtain approximately >20,000 paired-end reads per cell. ATAC-seq libraries were also sequenced with a NovaSeq 6000 sequencer (Illumina) using the following conditions: 50 bp (Read 1N) + 8 bp (i7 Index) + 16 bp (i5 Index) + 49 bp (Read 2N), aiming at a sequencing depth of >25,000 reads/nucleus.

#### CITE-Seq

Cryopreserved cells were thawed and FACS-sorted as previously described (see scATAC-seq and 3’ Single Cell RNA sequencing). For CITE-Seq experiments, samples were processed separately or processed in pools (subsequent demultiplexing by genotypes) before cell labeling with a custom panel of 192 oligo-barcoded antibodies (TotalSeq-C Custom Human Panel, Biolegend), following the same staining protocol of for Cell Hashing (see 3’ Single Cell RNA sequencing). Antibody details are included in Table S6. Cells were loaded on the 10X Chromium Controller using the Next GEM Single Cell V(D)J Reagent Kits v1.1 with Feature Barcoding technology (10X Genomics, CG00208) according to manufacturer’s instructions. Each sample was loaded in duplicate for a total target recovery of 5,000 cells (20,000 for sample pools).

After GEM dissolution and Dynabeads purification, 15 PCR cycles were done using the SC5ʹ Feature cDNA Primers (PN-1000080) to amplify the DNA from cell surface protein Feature Barcode oligos together with the full-length cDNA. The two products were separated by size selection and used for generating the different types of libraries. To construct the GEX library, the amplified full-length cDNA was fragmented, end repaired, A-tailed, and sample indexed using the Chromium Single Cell 5’ Library Construction Kit (10X Genomics, PN-1000020). For the V(D)J library, human T and B cell V(D)J sequences were enriched from the amplified cDNA with the Chromium Single Cell V(D)J Enrichment Kits (PN-1000005 and PN-1000016 for T and B cells respectively) followed by fragmentation, end repairing, A-tailing and sample indexing. Finally, the cell surface protein (CSP) library was generated from the amplified DNA from cell surface protein Feature Barcode by one-step PCR amplification using the Chromium Single Cell 5' Feature Barcode Library Kit (PN-1000080). Quantification and fragment size distribution of cDNAs and final libraries were determined using the Agilent 2100 BioAnalyzer High Sensitivity DNA kit (Agilent Technologies). All constructs were sequenced together on a Novaseq 6000 (Illumina), targeting a median sequencing depth of 20,000 (GEX), 2,000 (VDJ) and 8,000 (CSP) reads per cell.

#### Spatial Transcriptomics (Visium OCT)

Spatial visualization of gene expression within tonsil tissue was conducted using the Visium Spatial Gene Expression kit (10X Genomics) as per manufacturer's protocol. The OCT blocks were cut twice using a cryostat (Leica CM1950): a first time to assess RNA quality and assure a minimum RNA Integrity Number (RIN) number of 7 (RNA pico Chip) and a second time to mount a 10 μm section on the Visium slides. Slides were H&E stained before the sections were imaged using the NanoZoomer S60 (Hamamatsu) to assess tissue morphology and quality. The sections were then permeabilized for 6 min, according to the results of a corresponding Tissue Optimization experiment (10X Genomics, CG000238), and processed according to the Visium Spatial Gene Expression user guide (10X Genomics, CG000239). In short, tissue was lysed and reverse transcription was performed followed by second strand synthesis and cDNA denaturation. Spatially barcoded, full length cDNAs were amplified by PCR for 16 or 18 cycles, depending on the initial concentration previously determined by qPCR. Indexed sequencing libraries were generated via end repair, A-tailing, adaptor ligation and sample index PCR and analyzed using the Agilent 2100 BioAnalyzer. Libraries were sequenced on an Illumina NovaSeq 6000 with sequencing depth of ∼100,000 reads per spot.

#### FACS isolation of slan+ myeloid cells

Cryopreserved tonsils were thawed, washed in a cell staining buffer (Biologend), and counted to analyze cell viability. Thereafter, cells were resuspended in a cell staining buffer and stained with antibodies for the analysis of double negative T cells and the FACS isolation of SLAN+ cells. For the analyses of double negative T cells, tonsil cells were stained with anti-CD3 (PE/Cyanine7 anti-human, Biolegend), anti-CD4 (Alexa-Fluor 488 anti-human, Biolegend) and anti-CD8 (APCy7 anti-huma, Biolegend) and CD3+ and double negative CD4 and CD8 were analyzed. For the sorting isolation of SLAN+ cells, we stained cells with anti-CD3 (PE/Cyanine7 anti-human CD3 Antibody, Biolegend), anti-CD19 (PE/Cyanine7 anti-human CD19 Antibody, Biolegend), and anti-CD56 (PE/Cyanine7 anti-human CD19 Antibody, Biolegend) antibodies to exclude the lymphoid fraction. The anti-slan (M-DC8 Antibody, anti-human, Biotin, conjugated with Biotin antibody FITC, Miltenyi Biotec) antibody was used to select myeloid cells that were positive for slan in the tonsils. Then, we used anti-CD14 (PerCP/Cyanine5.5 anti-human CD14 Antibody, Biolegend) and anti-CD16 (APC/Cyanine7 anti-human CD16 Antibody, Biolegend) antibodies to sort monocytes and macrophages. Lastly, we used anti-CD11c (APC anti-human, Biotin, Miltenyi Biotec) and anti-CD123 (VioGreen anti-human, Miltenyi Biotec) antibodies to sort slan+ dendritic cells from cells that were negative for CD14 and CD16. We achieved a very high purity (>97%) using the Melody FACS flow cytometer (Becton Dickinson, Franklin Lakes, NJ). Accordingly, we sorted tonsil slan+ CD14+ CD16+ cells, slan+ CD11c+ CD123+ cells, and slan+ CD14- CD16- CD11c- CD123- cells from the myeloid fraction of tonsils. After sorting, cells were concentrated by centrifugation at 400 x g for 7 minutes at 4C and counted with a TC20™ Automated Cell Counter (Bio-Rad Laboratories, S.A). Slan+ Monocytes/Macrophages and slan+ dendritic cell fractions were pooled before proceeding to 10X Genomics 3’ single cell RNA-sequencing. Briefly, cells were partitioned into Gel BeadInEmulsions (GEMs) by using the Chromium Controller system (10X Genomics) with a target recovery between 2000 and 5000 cells. cDNA sequencing libraries were prepared using the Next GEM Single Cell 3’ Reagent Kits v3.1 (10X Genomics, PN-1000121), according to manufacturer's instructions. Size distribution and concentration of GEX libraries were verified on an Agilent Bioanalyzer High Sensitivity chip (Agilent Technologies). Finally, library sequencing was carried out on a NovaSeq 6000 sequencer (Illumina) using the following sequencing conditions: 28 bp (Read 1) + 10 bp (i7 index) + 10 bp (i5 index) + 90 bp (Read 2), to obtain approximately >20,000 paired-end reads per cell.

#### Cell lines and cell culture

KMS11 and MM1.R cell lines were kindly provided by Xabi Agirre (CIMA, Pamplona, Spain), and were expanded with RPMI-1640 (Invitrogen, Carlsbad, CA) with GlutaMAX containing 10% FBS (Gibco), 100 IU/ml penicillin and 100μg/ml streptomycin and kept at 37°C in a humidified incubator (5% CO2 and 95% atmosphere). XG6 and XG21 cells were kindly provided by Jerome Moreaux (IGH, Montpelier, France) and were expanded with RPMI-1640 with GlutaMAX containing 20% FBS, 2ng/ml IL-6, 100 IU/ml penicillin and 100μg/ml streptomycin and kept at 37°C in a humidified incubator (5% CO2 and 95% atmosphere). Cells were maintained at 0.15 mCell/ml (XG6) and 0.2 mcell/ml (XG21). Normal B cells were purified from buffy coats obtained from the Banc de Teixits i Sang (Barcelona). Peripheral blood mononuclear cells were isolated using Ficoll-Plaque plus density gradient and mature B cells were purified by AutoMACS selection of CD19 positive cells.

#### Protein extraction and western blot

Cells were lysed with RIPA buffer (50mM HEPES pH 7.6, 1mM EDTA, 0.7% Na deoxycholate, 1% NP-40, 0.5M LiCl) complemented with 6.25 mM NaF, 20mM β-glycerophosphate, 1 mM DTT, and 1X Protease Inhibitor Cocktail (Complete EDTA-free tablets, Roche, Basel, Switzerland). A total of 20 μg protein was separated by SDS-PAGE gel electrophoresis using 4-15 % Mini-Protean TGX precast gels (Bio-Rad, Hercules, CA, USA), blotted to nitrocellulose membranes (Thermofisher Scientific), and probed with the following primary antibodies: anti-Six5 (1:500, Proteintech 22938-1-AP, Rosemont, IL, USA), anti-Actin B (1:5000, Clone C4, MAB1501, Millipore, Burlingotn, MA, USA), at 4°C overnight. Antibodies were diluted in 5 % BSA in TBS-T. For visualization, the membranes were incubated with IRDye800CW goat-anti-rabbit or goat-anti-mouse IgG antibodies (1:5000, LI-COR, #925-32211 and 925-32210) for 1 h at RT and then scanned with an Odyssey DLX Imaging System (LiCor). PageRuler Plus Prestained Protein Ladder (Thermofisher Scientific, #26620) was used as molecular weight marker.

#### Multiplexed immunofluorescence of tonsil-resident CD8 T cells

##### Human mucosal tissues

Tonsils from patients undergoing tonsillectomy were received fresh after written informed consent, according to the Declaration of Helsinki. Approval was obtained by the ethics commission of the Charité-Universitätsmedizin Berlin (EA2/078/16), also in accordance with the local ethical guidelines.

##### Tissue preparation for multiplexed histology

Fresh frozen tonsils were cut 5 μm thick with a NX80 cryotome (ThermoFisher, Waltham, Massachusetts, USA) on 3-aminopropyltriethoxysilane (APES)-coated cover slides (24 × 60 mm; Menzel-Gläser, Braunschweig, Germany). Samples were fixed for 10 min at room temperature using a freshly opened EM grade PFA ampulla (methanol- and RNAse-free; Electron Microscopy Sciences, Hatfield, Philadelphia, USA) diluted to 2%. After thorough washing with PBS, samples were permeabilized with 0.2% Triton X-100 in PBS for 10 min at room temperature. Subsequently, samples were blocked for at least 20 minutes with 10% goat serum and 1% BSA in PBS. Afterwards, a fluid chamber holding 100 μl of PBS was created using “press-to-seal” silicone sheets (Life technologies, Carlsbad, California, USA; 1.0 mm thickness) with a circular cut-out (10 mm diameter), which was attached to the coverslip and surrounding the sample.

##### Image acquisition for multiplex histology

We used a modified Toponome Image Cycler® MM3 (TIC) originally produced by MelTec GmbH & Co.KG Magdeburg, Germany 16 for the acquisition of multiplexed histology data, as previously described.^42^ The robotic microscopic system consists of: (i) an inverted widefield (epi)fluorescence microscope Leica DM IRE2 (20 × /0.8 NA objective air lens, filter setup: Omega Optical XF116−2, AHF F46-010, AHF F46-009, and AHF F46-000) equipped with a CMOS camera (Orca®-flash4.0 LT, Hamamatsu Photonics GmbH, 2048 × 2048 pixels, pixel size 6.5 μm, no binning) and a motor-controlled XY-stage, (ii) CAVRO XL3000 Pipette/Diluter (Tecan GmbH, Crailsheim, Germany), and (iii) a software MelTec TIC-Control for controlling microscope and pipetting system and for synchronized image acquisition. The MELC run is a sequence of cycles, each containing the following four steps: (1) pipetting of the fluorescence-coupled antibody onto the sample, incubation, and subsequent washing; (2) cross-correlation based auto-focusing, which compares the current phase-contrast images with a phase-contrast reference image acquired at the beginning of each MELC run, defines the xyz position of the field-of-view (FOV) of interest within the whole sample, and thus corrects displacements in xyz for the aligned acquisition of 3D fluorescence image stacks for each marker (±5 z-steps; z-step = 1 μm); (3) photo-bleaching of the fluorophore using the optimal time span to minimize the fluorescence signal of each staining followed by washing of the specimen; and (4) a second autofocusing step followed by the acquisition of a 3D stack post-bleaching fluorescence image. In each four-step cycle, up to three fluorescence-labeled antibodies were used, combining PE, FITC, and DAPI. The antibodies for multiplexed histology of the tonsil are listed in Table S6, together with all related reagents, instruments and software used for the analysis.

##### Image pre-processing

After completion of each experiment, images were registered by cross-correlation based on the reference phase-contrast image taken at the beginning of the measurement, in order to account for the mechanical tolerance of the motorized microscope table. Subsequently, images were processed by background subtraction and illumination correction. Additionally, an “extended depth of field” algorithm was applied on the 3D fluorescence stack in each cycle. Images were then standardized in Fiji,^127^ where a rolling ball algorithm was used for background estimation, edges were removed (accounting for the maximum allowed shift during the autofocus procedure) and fluorescence intensities were stretched to the full intensity range (16 bit = >216).

### Quantification and statistical analysis

#### scRNA-seq: Data alignment

We used cellranger count (v4.0.0, 10X Genomics) to align reads to the GRCh38-2020-A human genome, with the “chemistry” parameter set to “SC3Pv3”. As cell-hashed and non-cell-hashed libraries had different target recoveries, we set the “expect-cells” parameter to 20,000 and 5,000, respectively. For cell-hashed samples, the “libraries” and “feature-ref” parameters were specified as described in the “Feature Barcode Analysis” pipeline of Cell Ranger. HTO sequences for each library can be found at Table S6.

For the additional samples from Newcastle, cellranger (V4.0, 10X Genomics) was used to align reads to the GRCh38-2020-A human genome using the default “chemistry” parameter and “expect-cells” parameter set to 7,000 cells.

#### scRNA-seq: Demultiplexing of HTO

We performed all downstream pre-processing with Seurat (v3.2.0 and v4.1.0). To normalize HTO counts, we applied a centered-log-ratio transformation across HTO, as implemented in the function “NormalizeData” (normalization.method = “CLR”, margin = 1). To assign an HTO to each cell, we used the “HTODemux” function (positive.quantile = 0.99). Briefly, this function performs k-medoid clustering (k = # HTO + 1) and uses the cluster with the lowest average to find the “negative” distribution for each HTO. Then, it fits a negative binomial distribution and uses the 0.99 quantile as threshold, which classifies cells as positive or negative for each HTO. We excluded cell barcodes not assigned to any HTO (“Negative”), as they had a lower library size and low number of detected genes. On the other hand, we kept cell barcodes assigned to two or more HTO to increase the statistical power and robustness of our doublet detection strategy (see below). To compare hashing efficiency across libraries, we computed a signal-to-noise ratio (SNR) for each cell as follows:

$$\text{SNR}=\frac{CLR-normalized\;counts\;\left(HTO1\right)+0.1}{CLR-normalized\;counts\;\left(HTO2\right)+0.1}$$

Where HTO1 and HTO2 are the HTO with the first and second largest counts for that cell, respectively.

#### scRNA-seq: Filtering and data normalization

We noticed that the library size distribution (total unique molecular identifiers; UMI) was higher in cell-hashed libraries than in non-cell-hashed samples. Following the current best practices,^128^ we determined the quality control (QC) thresholds for cell-hashed and non-cell-hashed libraries separately. Not to bias cell type composition, we decided to be as permissive as possible and applied more stringent thresholds at the cluster level. For non-cell-hashed libraries, we excluded cell barcodes with <1,000 UMI, <250 detected genes or a mitochondrial expression >20% (potential lysed cells or empty droplets). For cell-hashed libraries, we filtered out cell barcodes with <1,000 UMI, <400 detected genes or a mitochondrial expression >20%. In addition, we excluded genes detected in <=5 cells. A full discussion on why we chose these thresholds can be found at the associated reports available on GitHub. To adjust for differences in total UMI across cells, we used the function NormalizeData (normalization.method = "LogNormalize", scale.factor = 1e4). This function divides the raw gene counts for each cell by the total counts of that cell and multiplies it by the scale factor (10,000), which is then log-normalized as log(1+x).

#### scRNA-seq: Feature selection, dimensionality reduction and batch effect correction

Before clustering cells to define tonsillar cell types and states, we performed three important steps:

(1) calculate the proportion of doublet nearest neighbor for each cell (pDNN, described below);
(2) merge and integrate our dataset with the Seurat object from King et al.,^12^ and
(3) merge and integrate the resulting Seurat object with the RNA slot of our Multiome experiments.

The latter two allowed us to reach a robust consensus annotation across studies, to include an external control to ensure we preserved biological variability, and to connect chromatin accessibility with gene expression. Because we included new sets of cells in these steps, we reasoned that the set of highly variable genes (HVG) and axis of variability would change. Thus, for each step we performed the following steps:

1. We used the function FindVariableFeatures of Seurat (with default parameters) to extract the top 3,000 HVG for steps 1 (doublet detection) and 2 (integration with King et al.^12^ For step 3, we noticed that the three expression matrices (scRNA-seq, King et al., Multiome) consisted of a different set of genes, consistent with the poor mixability between single-cell and single-nuclei RNA-seq techniques.^129^ To homogenize it, we found the top 5,000 dataset-specific HVG and took the intersection (1,740 genes), which we used as input for the next two functions.
2. We performed a z-score transformation (Seurat: ScaleData, default parameters) on the normalized-values, followed by principal component analysis (Seurat: RunPCA, default parameters).
3. To correct for batch effects, we used Harmony v1.0,^107^ as a recent benchmarking effort reported that it scales well to hundreds of thousands of cells and it is amongst the best integration tools.^130^ We used the function RunHarmony, with the top 30 principal components (PC) as input. We considered cells coming from different GEM wells (see above) as different batches (specified in the group.by.vars parameter).
4. We then assessed the success of the data integration qualitatively with UMAP (Seurat: RunUMAP, first 30 PC, reduction = “harmony”) and quantitatively with the Local Inverse Simpson’s Index (lisi v1.0: compute_lisi, default parameters). This LISI score ranges from 1 to N (number of batches), and quantifies the diversity of batch labels on the neighborhood of each cell. Thus, the larger the LISI, the better the batch integration. We applied both approaches to measure the effect of six potential confounders before and after integration: library, sex, age group, hashing status, sampling center and assay (3’, 5’ or Multiome). To ensure we preserved biological heterogeneity, we plotted the cell type labels provided by King et al. on the aforementioned UMAP.

#### scRNA-seq: Doublet detection and removal

Although we considered cell hashing as our gold-standard method for doublet detection, it presents some limitations. First, cell hashing cannot detect intra-index doublets (doublets with the same hashtag/index). Second, hashing efficiency can vary across libraries (measured by signal-to-noise ratio). Finally, non-hashed libraries will have a doublet rate at approximately 4% per sample. To mitigate these issues, we ran Scrublet v0.2.1,^119^ which simulates and predicts doublets computationally. Following the best practices, we ran scrublet for each library separately. Since we expected a doublet rate of 4% for a target recovery (TR) of 5,000 cells, we set the “expected_doublet_rate” of the “Scrublet” function to 0.04 and 0.16 for non-hashed (TR=5,000) and hashed libraries (TR=20,000), respectively. For the scrub_doublets function, we set the parameters min_counts to 2, min_cells to 3, min_gene_variability_pctl to 75 and n_prin_comps to 50. The resulting doublet scores (which range from 0 to 1) and predictions (True or False) were added to the metadata of the Seurat object.

Notably, both cell hashing and scrublet were run for each library independently. Thus, we aimed to combine both approaches in a single metric that can consider all cells in the dataset, hence increasing the statistical power to detect doublets. To this end, we computed the pDNN, a metric inspired by the proportion of artificial nearest neighbors (pANN) introduced by DoubletFinder.^131^ Briefly, we used the top 30 harmony-corrected PCs to find the 75-nearest neighbors for each cell (Seurat: FindNeighbors). Then, we calculated a pDNN for each cell by dividing the number of nearest neighbors labeled as doublets by the neighborhood size (75). We followed this approach for three doublet annotations: cell hashing, scrublet and their union. Finally, we observed that the regions of the UMAP with the highest pDNN values corresponded to cell neighborhoods that expressed two or more markers of different lineages (CD3D and CD79B, for example); which validated its use. Overall, we excluded 80,577 doublets labeled by cell hashing; and flagged the ones detected by scrublet. In addition, we kept the pDNN scores in the metadata to filter out clusters of doublets downstream.

#### scRNA-seq: Clustering and annotation

To cluster cells into cell types and states, we followed a top-down, recursive approach, organized from general to specific. This approach is inspired by the mouse brain atlas.^132^ At each level, we performed Louvain clustering by first calculating a K-nearest neighbors graph (Seurat: FindNeighbors, reduction = “harmony”, top 30 PC), and then determining the exact clusters (Seurat: FindClusters). The “resolution” parameter of FindClusters is what drives the number of clusters, and it was adjusted differently at each level. At each level, we subsetted one or more clusters, thus increasing our ability to detect finer-grained heterogeneity. Therefore, at each level we have rerun steps 1-3 described above to find HVG, perform PCA and correct for batch effects. Every level was an opportunity to fetch and discard clusters of poor-quality cells and doublets using the different sources of evidence we gathered in the analysis explained above. Below a more detailed explanation of each cluster level:

- Level 1: we reasoned that, if clusters represent stable categories, we should be able to classify unseen transcriptomes to those categories with high accuracy. Thus, we fitted a random forest classifier (randomForest package) using the top 30 harmony-corrected PC as features to predict clusters derived from varying clustering resolutions. We plotted the resulting out-of-bag accuracies as a function of the clustering resolution, and determined an “elbow” in the plot to find the optimal resolution (0.25), which resulted in 12 clusters. We performed a “one-vs-all” differential expression analysis to find markers specific to each cluster (Seurat: FindMarkers, test.use = “wilcox”). After interpreting the top markers per cluster, we split and merged them into a biological sound manner. For example, the epithelial cells clustered together with the myeloid; and the precursor T and B cells clustered with the PDC. Moreover, we removed one cluster that showed a high pDNN score and no distinctive markers. Overall, we identified nine major cell compartments, which correspond to the categories in Figure S1D.
- Level 2: we split the main Seurat object into nine (one per major compartment), and rerun the data integration pipeline. Moving forward, we considered “assay” (scRNA-seq or Multiome) as the batch variable to correct for, as it was the main driver of variance. In this step, we had high statistical power to remove clusters of poor-quality cells [low number of detected genes or UMI, high mitochondrial/ribosomal expression, and/or high expression of stress-related markers (*JUN*, *JUND*, *FOS*)] and doublets [high pDNN, expression of markers from two mutually exclusive lineages (e.g. CD3D and CD79A), and/or positive scrublet annotation].
- Level 3: for cell compartments with fewer cells (myeloid, FDC, PDC and epithelial), we excluded both cells from King et al.^12^ dataset and from Multiome; because we reasoned that our assay-specific feature selection and integration strategy would overcorrect biological heterogeneity in these underrepresented compartments. In addition, since epithelial cells were composed of fewer than 1,000 cells, we used only the top 20 PCs and reduced the neighborhood size (k) from 20 to 10.
- Levels 4–5: we leverage the annotation from King et al.^12^ as a starting annotation, and hereafter focused solely on our own data. Because biology-driven clustering led to more meaningful cluster labels, we discarded the random forest strategy. We found markers for each cluster with FindMarkers, as explained above. We developed a Shiny app that allowed annotation experts to explore the expression of marker genes and to determine a biologically sound clustering. Further, we used the FindSubCluster (graph.name = "RNA_snn") function from Seurat to stratify heterogeneous clusters, varying the resolution parameter to identify the optional number of subclusters. Of note, we moved naive CD8 T cells from the CD4 T cell compartment to Cytotoxic cells.

#### scRNA-seq: Gene signature scoring

To collapse the expression of a set of genes into a per cell gene signature, we used the AddModuleScore function from Seurat with default parameters. Later, we used the AddModuleScore_UCell function from the UCell package with default parameters because it was shown to outperform Seurat’s AddModuleScore.^109^ Finally, we used the CellCycleScoring function and the predefined list of cell cycle markers from Seurat (cc.genes variable) to calculate a per cell S.Score and G2M Score, as well as to classify cells into cell cycle phases. To obtain the endoplasmic reticulum (ER) signature in plasma cells, we first performed a differential expression analysis (DEA) between the LZ-GCBC and short-lived IgM+ plasma cells and we then used the Database for Annotation, Visualization and Integrated Discovery (DAVID)^133^^,^^134^ to perform a KEGG pathway enrichment analysis using the upregulated genes.

#### scRNA-seq: Gene Set Enrichment Analysis

To find specific functions associated with each slan-like subset, we conducted a one-vs-all differential expression analysis for each slan-like subset (Seurat: FindMarkers, only.pos = FALSE, logfc.threshold = 0). We arranged the resulting gene list by decreasing log_2_ fold-change. We performed a gene set enrichment analysis (GSEA) for each gene list using the function gseGO (ont = "BP", OrgDb = org.Hs.eg.db, keyType = "SYMBOL", minGSSize = 10, maxGSSize = 250) from clusterProfiler v4.3.4.^108^ We filtered out gene ontology (GO) terms with an adjusted p-value > 0.05, and arranged them by decreasing normalized enrichment score (NES). Finally, we plotted the running enrichment scores with the gseaplot function for selected GO terms.

#### scRNA-seq: Validation with external datasets

To validate the upregulation of SIX5 in plasma cells using external datasets, we queried the human cell atlas Bone Marrow Viewer, available here^77^ and the “Blood (PBMC) Hao” single cell RNA-seq track^18^ at the UCSC genome browser. In addition, we downloaded ChIP-seq data of the histone mark H3K27ac, which was generated as described here.^76^^,^^135^ The normalized signal from this histone mark was captured for SIX5 and its target genes, except for TSC22D3 gene (located at chromosome X), in tonsillar Naive B cells (NBC) (n=3); NBC from peripheral blood (n=3); Germinal Center B cells (n=3), class-switch Memory B cells (n=2); non-class switch Memory B cells (n=1); Plasma cells (n=3); MM (n=4). To identify slan-like subpopulation, we downloaded the differentially expressed genes between tonsillar slan+ cells, CD11b+CD14+-macrophages, and CD1c+DCs/cDC2 from the Table S4 (file name: “fsb220611-sup-0002-tables4.xlsx”) of a recent study.^91^

#### scRNA-seq: Cell cycle regression

To ensure that the proliferation signature did not obscure relevant cell-cell heterogeneity in the germinal center B cell (GCBC) dataset, we employed two independent approaches for its correction. First, we excluded genes associated with the cell cycle from downstream analysis (PCA, Harmony, UMAP). To identify these cell cycle-associated genes, we followed the steps outlined in Chapter 9.5 of the book "Orchestrating Single-Cell Analysis with Bioconductor".^93^ In summary, we calculated the percentage of variance explained by the cell cycle phase for each gene (scater v1.26.1: getVarianceExplained with default parameters).^136^ The cell cycle phase had been previously determined using the CellCycleScoring function from Seurat. We then filtered out 515 genes from the initial set of 2,500 HVG that had a percentage of variance explained greater than 5%. Subsequently, we performed PCA, Harmony, and UMAP using the remaining 1,985 genes not associated with cell cycle phase. Second, we regressed out the cell cycle scores (“*S.Score*”, “*G2M.Score*”) following the “Cell-Cycle Scoring and Regression” vignette from Seurat. Briefly, we ran the ScaleData function with the vars.to.regress parameter set to “*S.Score*” and “*G2M.Score*”. This function models gene expression as a function of S and G2M cell cycles scores, and keeps the scaled residuals for downstream analysis, including PCA, Harmony and UMAP.

#### Gene regulatory network inference

To infer transcription factor (TF) activity, we used pySCENIC v0.10.3^24^ on the scRNA-seq raw matrices as well as SCENIC+^25^ on the multiome matrices from T cells and B cells separately. Briefly, pySCENIC infers co-expression modules (known as regulons), composed of a given TF and its putative target genes, and measures their activity in each individual cell. GRNBoost2 algorithm from the Arboreto package^137^ was used to infer the co-expressed modules from a predefined curated list of 1,797 human TFs, provided in the pySCENIC repository. Next, cisTarget^122^ was applied to refine regulons by pruning indirect targets based on cis-regulatory motifs footprints using human motifs v9 and hg38 (500bp upstream of TSS, and 100 bp downstream) from cisTarget databases. This process gave a total of 189 and 214 regulons for T cells and B cells respectively. For SCENIC+, scRNA-seq data was preprocessed using Seurat as previously described and scATAC-seq data was processed using pycisTopic as described here. We filtered cells based on scRNA-seq analysis, keeping all cells that passed quality metrics in scRNA-seq. A model of 50 and 45 topics were selected for B and T cells respectively and pycistarget^25^ was used to binarize the topics and identify differentially accessible regions between cell types. Next, pycistarget was applied to identify enriched motifs in the identified candidate enhancer regions using the precomputed motif database and the motif-to-tf annotation database available here. Finally, enhancer-driven gene regulatory networks were inferred using SCENIC+ as described here. Regulon specificity score (RSS) were computed using the Jensen-Shannon Divergence.^138^

#### scATAC-seq: Data alignment

We used Cell Ranger ATAC v1.2.0 to map the fastq files using GRCh38-1.2.0 as the human reference genome. Specifically, we run cellranger-atac count command on each individual library to perform read filtering and alignment, barcode correction and counting, and peak calling. Then, to pool the samples together, we used cellranger-atac aggr command with a new round of peak calling. To maximize the sensitivity of the input libraries, we set the normalization model to “None”.

#### scATAC-seq: Data quality control

We performed all downstream analysis with Seurat v3.9.9 and its extension package Signac v1.1.0.^106^ The total number of non-filtered aggregated cells were 64,162 with 5,724 median fragments per cell, 71,3% fraction of fragments overlapping any targeted region and a 52.7% fraction of transposition events in peaks in cell barcodes. We determined the QC thresholds for each library individually by applying non-restrictive thresholds. At this point, we noticed that the fraction of fragments falling within the peaks was significantly higher in FACS-processed libraries than in non-FACS libraries. To remove low-quality cells from the aggregated libraries, we excluded cells that presented: (1) a total number of fragments in peaks lower than 700 or greater than 30,000; (2) had a fraction of fragment in peaks lower than 15; (3) a transcriptional start site enrichment score lower than 2; (4) a ratio of reads assigned to blacklist regions greater than 0.03; (5) and cells with peak counts lower than 500 or greater than 100,000. In addition, we excluded peaks detected in 5 or fewer cells. After this filtering step, we end up with 58,049 high-quality cells. A full discussion on the reasoning behind these thresholds can be found at the associated reports available on GitHub in the following link.

#### scATAC-seq: Data normalization and Integration

We merged and integrated the scATAC-seq dataset with the ATAC slot extracted from the Multiome experiments (described below). This approach allowed us to (1) increase the number of scATAC-seq cells, (2) reach a consensus chromatin profile not biased by the techniques, (3) create a direct link between ATAC peaks and gene expression and (4) transfer both the cluster labels and UMAP coordinates defined with gene expression to the scATAC-seq dataset (see below). When merging multiple single-cell chromatin datasets, it is essential to note that the peak calling was done for each dataset independently. Thus, we first created a consensus set of peaks across all datasets using the *UnifyPeaks* function (mode = "reduce"). We then filtered out peaks with a width lower than 20 bp or greater than 10,000 bp. We quantified the accessibility in the consensus peaks in each dataset using the *FeatureMatrix* function, and then used these unified matrices to create new Seurat objects that were finally merged using the *merge* function. This resulted in 101,279 cells and 166,156 peaks. For each dataset individually and then for the merged dataset (scATAC-seq and Multiome), we ran the following pipeline:

1. We applied the term frequency-inverse document frequency (TF-IDF) normalization (Signac: *RunTFIDF*, method =1, with the default parameters). TF-IDF corrects for differences in library size across cells, and penalizes peaks that are homogeneously open or closed across all cells.
2. We set the *FindTopFeatures* function to q0 to consider all the features for the dimensional reduction step. To obtain a reduced dimension representation of the dataset, we ran the singular value decomposition (Signac: RunSVD, default parameters) on the TF-IDF-normalized data. Because the variance captured by the first LSI component was explained by library size, we decided to exclude it for downstream analysis.
3. We used Harmony v1.0,^107^ to correct for batch effects because it is amongst the best-performing integration tools for scATAC-seq data.^139^ Specifically, we executed the *RunHarmony* function with default parameters selecting from the second to the n first LSI components (n is variable depending on the dataset analyzed) and group.by.var equal to "gem_id" (GEM well) or "assay".
4. To assess Harmony’s performance, we used the LiSi score^3^ to verify the quality of the data integration across 5 main categorical confounders: sex, age group, sampling center, library and assay or technique applied in the dataset.

#### scATAC-seq: Doublet detection

To predict the potential doublets from single-cell ATAC-seq data, we accumulated different sources of information:

1. We used a modified version of Scrublet v0.2.1^119^ to compute the cell doublet score per library following these parameters: log_transform=True, min_counts=2, min_cells=3, min_gene_variability_pctl=70, n_prin_comps=50. Note that for the BCLL-14-T and BCLL-15-T samples, the expected doublet rate was set to 0.056 (TR=7,000 nuclei) compared to the rest that was set at 0.04 (TR=5,000 nuclei). We flagged as True the predicted doublets defined by the automatic threshold provided by Scrublet and this information was added to the Seurat object metadata.
2. To discard doublets, nuclei clumps and other artifacts, we removed cells with extremely high numbers of fragments in peaks.

#### scATAC-seq: Gene Activity Matrix

We represented scATAC-seq profiles as peaks (features) and integrated them with Harmony, because a recent benchmarking study showed that these were amongst the best options for scATAC-seq analysis.^139^ Although the same study concluded that gene activities are poorly suited to represent scATAC-seq profiles, we aimed to verify that in our own dataset. To this end, we followed a similar split as in our original approach, focusing on the CD4 T cells. Thus, we used Multiome cells as reference (RNA slot), and scATAC-seq as query (represented as gene activities). To compute these gene activities, we used the GeneActivity function from Signac (with default parameters), which adds up all the counts in gene body and promoter region (starting 2000 bp upstream the transcriptional start site). Gene activities were subsequently added to the Seurat object and normalized (Seurat: NormalizeData, normalization.method = "LogNormalize", scale.factor = 10000). After finding the top 3,000 HVG for the reference, we integrated reference and query using Seurat’s canonical correlation analysis (CCA, Seurat: FindTransferAnchors, reduction = “cca”), and transferred cell type annotations from reference to query (Seurat: TransferData, dims = 2:30, weight.reduction = “lsi”). To compare both annotations (Harmony + KNN and gene activity), we calculated accuracy and kappa statistics with the confusionMatrix function from the caret v6.0.93 package with default parameters. To annotate peaks to their respective genomic annotations (e.g. promoter, intron, etc.), we used the annotatePeak (tssRegion = c(-2000, 0), annoDb = "org.Hs.eg.db", TxDb = TxDb.Hsapiens.UCSC.hg38.knownGene) function from ChipSeeker v1.34.1 package.^120^

#### Multiome: Data alignment

We used Cell Ranger v1.0 to map the fastq files to the GRCh38-2020-A as a human reference genome. Specifically, we run cellranger-arc count on each individual library to perform read filtering and alignment, barcode correction and counting, peak calling and counting of both ATAC and GEX molecules. A detailed description of all the quality control parameters evaluated can be found at the associated reports available on GitHub.

#### Multiome: Data quality control

The downstream analysis was done in R applying Seurat v3.9.9 and its extension package Signac v1.1.0. The total number of non-filtered cells was 77,006. To remove low-quality cells from each library, we applied the following filters: (i) for GEX, we excluded cell barcodes with fewer than 550 UMI, fewer than 250 detected genes or a mitochondrial expression higher than 20%, (ii) for ATAC, we excluded cells that presented a total number of transposition events lower than 500 or greater than 100,000, that had a transcriptional start site enrichment score lower than 2 and a nucleosome signal lower than 2, which resulted in 69,118 filtered cells. Note that the BCLL-2 sample was excluded from the scATAC-seq analysis because it presented a low overall quality that could lead to a misinterpretation of the data. After calling peaks individually for each library, we merged the libraries using the *UnifyPeaks* function (with default parameters), which aligns the ranges of peaks and merges the overlapping ones to produce a simplified set of intersecting peaks. We filtered out peaks with a width size less than 20 bp or greater than 10,000 bp. We quantified the accessibility counts in the new set of peaks using the *FeatureMatrix* function. Since the peaks were common across libraries, we merged all cells from all libraries into a single accessibility matrix.

#### Multiome: Doublet detection

To predict the potential doublets from single-cell ATAC-seq data, we accumulated different sources of information:

1. We used a modified version of Scrublet v0.2.1^119^ to compute the cell doublet score per library following these parameters: log_transform=True, min_counts=2, min_cells=3, min_gene_variability_pctl=70, n_prin_comps=50. Note that for the BCLL-14-T and BCLL-15-T samples, the expected doublet rate was set to 0.056 (TR=7,000 nuclei). We flagged as True the predicted doublets defined by the automatic threshold provided by Scrublet and this information was added to the Seurat object metadata.
2. To discard doublets, nuclei clumps and other artifacts, we removed cells with extremely high numbers of fragments in peaks.

#### Multiome: Data normalization and Integration

The data was treated as individual scRNA-seq and scATAC-seq objects and we repeated the standard downstream analysis explained above including data normalization, variable gene detection, data scaling, dimensionality reduction analysis, batch correction with Harmony and UMAP representation. To identify the nearest neighbors for each cell based on the weighted combination of the scRNA-seq and scATAC-seq modalities, we applied *FindMultiModalNeighbors* function to construct a weighted nearest neighbor (WNN) graph. The harmony integration of each modality was used as a dimensionality representation of each object using the first 30 PCs for scRNA-seq and the first 40 LSI components for scATAC-seq.

#### Alignment of scATAC-seq with Multiome datasets

To help the interpretation of the scATAC-seq integrated dataset, we classify the Multiome ATAC-seq cells using the annotation previously defined by the scRNA-seq from the same experiment, since the cells share the same cellular barcode. To extend the annotation to the rest of the scATAC-seq cells, we applied a k-nearest neighbour (KNN) classifier to annotate those cells to a given cell type category with the help of our Multiome training set. Note that KNN works on a basic assumption that data points of similar categories are closer to each other. To cross-validate the number of nearest neighbours to consider (the K parameter), we split our training set in two parts: a train.loan, that corresponds to the random selection of the 70% of the training set and the test.loan, that is the remaining 30% of the data set. The first one was used to train the system while the second was used to evaluate the learned system. We built the machine learning model using the optimal k. Note that the probability of the prediction was lower in the transitioning cells and in not-defined clusters.

#### Peak calling based on annotation levels

To identify more precise and specific peaks on the annotated cell types, we decided to do multiple rounds of peak calling using MACS2 v2.2.7.1^110^ as we increase the level of the clustering resolution. Specifically, we used the *CallPeaks* function provided by Signac setting the group.by parameter by the corresponding annotation level, removing peaks on non-standard chromosomes and on genomic blacklist regions. We quantified the new peak counts in the specific dataset by generating a consensus peak set and repeated the standard downstream analysis explained above; including data normalization, dimensionality reduction analysis, batch correction with Harmony and UMAP representation.

#### scATAC-seq specific chromatin features

To find differentially accessible features between the clusters defined at level 1, we performed a differentially accessibility (DA) test between all of them. We use a logistic regression^140^ adding the nCounts_peaks as a latent variable to mitigate the effect of sequencing depth (Signac: FindAllMarkers). Next, we filtered out the DA peaks with a Bonferroni-adjusted p-value less than 0.05 and selected the top 2,000 DA peaks to create the list of features needed to calculate the chromatin signature. To do that, we compute the ChromatinVar deviation for each cell type applying the *ChromatinModule* function. A more restrictive analysis was done to select specific DARs in the context of GCBC cells. In this case, a score was computed to identify regions with high accessibility of one cell type in relation to the others. Specifically, for each region, we computed the ratio between the maximum accessibility value across cell types to the sum of all accessibility values for that region. Then, the top scoring DARs that belonged to clusters of interest were selected.

#### Motif analysis

To find the consensus binding motifs in the DNA sequences, we used the chromVAR v1.1.0 R package,^111^ which calculates for each motif annotation and each cell, a bias-corrected “deviation” in accessibility from an expected value based on the average of all the cells. This allowed us to visualize motif activities per cell in each of the clusters. Motif annotation was performed using two databases to compare the robustness of the results. Specifically, we downloaded 746 transcription factor motifs from the JASPAR vertebrates core (using JASPAR2020 v0.99.10 R package),^124^^,^^125^ and 1,764 from CisBP database using human_pwms_v1, the curated collection of human motifs (as included in the R package chromVARmotifs v0.2.0).^111^ Based on the underlying question, we performed two different types of analysis: (1) identification of overrepresented motifs in a set on genomic regions using *FindMotifs* function provided by Signac or (2) differential testing on the chromVAR z-score using the *FindMarkers* function between the clusters to compare.

#### Estimating co-accessible sites

To enhance the interpretation of the scATAC-seq data, we decided to use the Cicero v1.3.4 R package^112^ that allows: (1) estimating the co-accessible sites in the genome, and (2) predicting potential cis-interacions between proximal/distal regulatory elements and their putative target genes. Specifically, we first converted the CD4 T Seurat object to CellDataSet format and to the Cicero object, using the Signac-provided functions: *as.cell_data_set and make_cicero_cds* respectively. For the *make_cicero_cds* function, we specified the coordinates in low-dimensional Harmony space. Then, we executed the wrapper function called *run_cicero* (with the default parameters) to get the pairwise co-accessibility scores for all the peaks identified in CD4 T cells.

#### Validation of the Tfh-specific BCL6 distal enhancer with external datasets

We obtained single-end H3K27ac ChIP sequencing FASTQ data for T follicular helper cells and non-follicular T effector cells populations from published data.^30^ This dataset is accessible through the Sequence Read Archive (SRA) repository (reference series GSE58597), and we downloaded it using the SRA toolkit v3.0.2. FASTQ files were aligned to genome build hg38 (using bwa v0.7.17,^141^ picard v2.24.0 and samtools v1.9^142^) following the Blueprint pipeline, available here. Following peak calling, we visualized the signals in the *BCL6* distal enhancer region in the UCSC browser.

Pseudobulk scATAC-seq genome tracks from human tonsillar T cell populations, including peak2gene predictions from ArchR,^143^ were obtained from King et al.^13^ and visualized with pyGenomeTracks.^144^ Peak2gene predictions with correlation > 0.4 and FDR < 0.01 were deemed significant and plotted in blue.

#### CITE-seq: Data alignment

We used Cell Ranger v6.0.1 multi to align simultaneously 5’ scRNA-seq, antibody profiles and TCR/BCR-seq, enabling consistent cell calling between the library types. Specifically, Cell Ranger uses the fastq files from all four modalities and performs alignment to the GRCh38-2020-A reference, filtration, feature barcode and UMI counting for both genes and antibody tags; along with the VDJ sequence assembly and clonotype counting (GRCh38-alts-ensembl-5.0.0 as the human genome reference).

#### CITE-seq: Genotype demultiplexing

In the batch BCLLATLAS_38, we pooled cells from four donors (BCLL-2, BCLL-6, BCLL-10 and BCLL-12) into a single experiment to identify doublets, reduce batch effects and sequencing costs. Genotypes were subsequently demultiplexed using Vireo v0.5.0^113^ based on individual genotypes inferred from scRNA-seq read information. First, we used cellsnp-lite v1.2.0^114^ to pileup the mapped reads at each single nucleotide variant (SNP), filtering variants with fewer than 20 UMIs and a minor allele frequency lower than 10% in the compiled list of 7.4 million common variants (AF>5%) present in 1000 Genome Project and gnomAD. Then, the pileup allelic profile of each cell barcode was used for donor deconvolution and doublet detection using Vireo. Out of 6,679 multiplexed cells, 380 were detected as doublets and 301 were unassigned cells.

#### CITE-seq: Quality control

We performed the downstream analysis using Seurat v4.0. Specifically, the quality control was performed in two main stages. First, cells assigned as doublets by Scrublet^119^ and genotype doublets and unassigned donor cells by Vireo were eliminated. Following the current best practices,^128^ we performed QC on each CITE-seq experiment separately. Cells outside of the threshold range of mitochondrial content, UMI counts and feature count set per subproject were filtered. After this first filtering step, we obtained a total of 42,929 cells, of which 12,867 had BCR (B cell repertoire) and 7,795 TCR (T cell repertoire) information. The QC plots and exact filtering thresholds can be found at the GitHub repository (see code availability section). Next, a top-down approach was used for a second quality control. We zoomed into T and B cell clusters to refine the quality based on marker expression. We removed B cells that exhibited a high expression of T cell marker genes (such as CD3, CD4 and CD8) and T cells with high expression of B cell markers (such as CD19, CD5, CD27) as well as cells with dual repertoire (both TCR and BCR) as potential doublets. 40,396 high-quality cells entered the subsequent analysis.

#### CITE-seq: Data normalization and Integration

We performed data normalization and integrations as follows:

• Normalize and correct for batch effects: the gene expression matrix was normalized as described before for scRNA-seq. For antibody-derived tags (ADT) data, we applied a centered log ratio (CLR) across cells (Seurat: NormalizeData, margin=2) and corrected for batch effects using Harmony.^107^
• Weighted-nearest neighbor (WNN) graph-based integration: The normalized and homogenized matrices were used for the construction of WNN graphs based on cell-specific data modality weights. This enables dimensionality reduction based on the weight of both modalities (Seurat: FindMultiModalNeighbors). We used the first 30 and 20 harmony-corrected principal components for RNA and ADT, respectively.
• Label Transfer: SLOcator was used to transfer labels and coordinates defined with scRNA-seq to CITE-seq (see below).

#### CITE-seq: Repertoire analysis

We used Scirpy v0.7.0^115^ to analyze TCR and BCR repertoires. Each repertoire sequence is formed by V, D and J gene recombination. Structurally, each of the repertoire representations consists of the framework region (FWR) and complementarity determining regions (CDR), which primarily interacts with the epitope. Clonotypes can be defined by using different algorithms (such as identity, blosum matrix based similarity, Hamming distance and Levenshtein distance) and sequence levels (nucleotide and amino acid). Here, we defined clonotypes based on the CDR3 nucleotide sequence identity and V gene usage (Scirpy: define_clonotypes, default parameters) within samples. For TCR and BCR analysis, we defined clonotypes expanded if three or more cells showed the same sequence (Scirpy: clonal_expansion, default parameters).

#### ST: Data processing

We used spaceranger count v1.1.0 to align the fastq files to the GRCh38-2020-A as a human reference genome. For each tissue slice we ran spaceranger count with its specific Visium slide ID, its capture area and the specific image in.jpeg format, these can be found in the project's github (see code availability section). Visium slide-specific spot layouts were used for each slide to determine the spatial coordinates of each spot.

#### ST: Quality control

Downstream analysis was done in R 4.0.1 and Seurat v4.1.0. We first assessed the distribution of library size, detected genes, and mitochondrial and ribosomal percentage across the slide to assess for overpermeabilization and subsequent lateral diffusion of reads. We noticed that the distribution of the library size highly correlated with histological features. Therefore, we decided to keep all spots overlaying the tissue. In the slide from sample BCLL-8-T there was a region that had been folded onto itself. Spots overlapping this folded region were removed since they showed lower library size and number of detected genes as well as a transcriptomic profile that reflected a mixture of regions. We determined mitochondrial and ribosomal percentage by dividing the number of UMIs assigned to genes starting with MT- or RPL|RPS respectively over the total library size for each spot. A detailed description of all the quality control parameters evaluated and environment used can be found at the associated reports available on GitHub (see code availability section).

#### ST: Data normalization

To adjust for differences in total UMI across spots, we used the function *NormalizeData* (normalization.method = "LogNormalize", scale.factor = 1e4). This function divides the raw gene counts for each cell by the total counts of that cell and multiplies it by the scale factor (10,000), which is then log-normalized as log(1+x).

#### ST: Feature selection, dimensionality reduction and batch effect correction

We first used the function *FindVariableFeatures* of Seurat (with default parameters) to extract the top 3,000 HVG. We compared this gene set with the ones obtained from algorithms aiming to detect spatially variable genes such as *FindSpatiallyVariableFeatures*, *spatialDE*^145^ and *SPARK*^146^ and found considerable overlap. We then proceeded to the downstream analysis with the HVG obtained with *FindVariableFeatures* due to the lower computational time required. Next, we performed a z-score transformation (as implemented in the function *ScaleData*) on the normalized-values. We then carried out PCA dimensionality reduction with the function *RunPCA* (default parameters). At this point batch effects were observed in the PCA space, therefore, we corrected for batch effects using Harmony v1.0,^107^ as a recent benchmarking effort reported it to be amongst the best three performing integration tools.^130^ We used the function *RunHarmony* with the top 20 principal components (PC) as input, the top 20 was decided after looking at the PCA elbow plot. We considered each tissue slice as different batches as each one was processed in a different capture area (specified in the group.by.vars parameter). Full analysis of the integration and batch correction can be found in the GitHub repository.

#### ST: Tissue region clustering and annotation

To annotate our tissue slices we followed two approaches. In the first approach, we carried out an unbiased data-driven approach in which we aimed to cluster the spots and annotate them using differentially expressed genes. In the second approach, expert pathologists manually annotated each tissue slide. Spot clustering was performed by using the functions *FindNeighbors*, which computes a shared nearest neighbor graph on the harmony integrated embedding; first 20 Harmony components were used, for all the spots. We then identified clusters of spots by using shared nearest neighbor (SNN) modularity optimization based Louvain clustering algorithm using *FindClusters* function. We computed the clustering with varying degrees of resolution to assess which one fit best our datasets. Annotation of tissue regions was carried out using resolution 0.3. We used the function *FindAllMarkers* to identify differentially expressed genes between clusters using the Wilcoxon rank sum test on the log-normalized gene expression. Prior knowledge marker genes were used to determine the identity of each cluster. Manual annotation by pathologists was carried out using a custom built Shiny App that allowed to select spots on the tissue and download the spot barcode for each selection.

#### ST: Cell type deconvolution

Integration of spatial transcriptomics with the reference scRNA-seq to obtain cell type deconvolution was performed using SPOTlight v0.1.7.^116^ Different SPOTlight runs were carried out for the different populations of interest as described below.

To deconvolute major cell types we used the annotation column *annotation_figure_1* (which corresponds with the annotation in Figure 1B). From this annotation we consolidated all the cycling cell types into one, *Cycling*, so the cycling signature did not drive the deconvolution of individual cell types. We then computed differential expressed genes between all populations Seurat’s function *FindAllMarkers* with the default Wilcoxon rank sum test and up to 500 cells per cell type. Next, we randomly sampled up to 50 cells per cell type from as few batches as possible to reduce the batch effect. Lastly, we ran deconvolution using the previously selected cells and the gene set resulting from union between the 3,000 most highly variable genes and the differentially expressed genes. Cell types predicted to contribute <3% of a spot were considered to be 0.

For CD4 T cell specific deconvolution, we used a combination of the annotation column *annotation_20220215* and *annotation_level_1*. This combination allowed us to have finer grained annotation for the CD4 T subpopulations, while maintaining the level-1 annotation for the other major cell types. This ensured that we captured the signal provided by the main cell types, while considering CD4 T heterogeneity. From the finer-grained annotation, we consolidated CM Pre-non-Tfh, CM PreTfh into one cell type and labeled them as CM PreTfh/Pre-non-Tfh, since they presented very similar phenotypes. We excluded preBC, preTC as they represent very few cells overall and introduced undesired noise to the model. We also excluded those cells annotated as CD4 T cells in level-1 but not annotated as a CD4 subpopulation at the more granular level. We then carried out two rounds of differential expression. The first was carried out among all the cell types using the annotation specified in *annotation_level_1*. This allowed us to capture differentially expressed genes between major populations. As before, we used Seurat’s function *FindAllMarkers* with the default Wilcoxon rank sum test and up to 200 cells per cell type. The second round of differential expression was computed only between the CD4 T subtypes to capture genes more subtly differentially expressed between them. Both lists of differentially expressed genes were filtered by logFC, pct.1 and p value to keep only relevant genes. We then randomly sampled up to 100 cells per cell type from as few batches as possible to reduce the batch effect. Lastly we ran deconvolution using the previously selected cells and the gene set resulting from union between the 3,000 most highly variable genes and the differentially expressed genes. Cell types predicted to contribute <3% of a spot were considered to be 0 (see *spatial_transcriptomics/CD4-Analysis/CD4-deconvolution.Rmd*). Lastly, for epithelial cells we followed the same approach to the one described above for CD4 T cells (see *spatial_transcriptomics/epithelium_integration/epithelium-deconvolution.Rmd*).

#### ST: Gene expression denoising

Due to the sparse nature of the data, we denoised the expression of genes of interest using MAGIC, Rmagic v2.0.3 package^117^ to gain a better understanding of the spatial distribution of their expression. We performed MAGIC denoising for gene sets of interest related to CD4 T cells, plasma cells, myeloid cells and Follicular Dendritic cells. MAGIC was run for each slice independently to avoid contaminating expression signal between them. The *knn* parameter was set to a conservative 2 to avoid over-diffusion, furthermore *t* was set to “auto” to determine the extent of diffusion according to the Procrustes disparity. The remaining parameters were kept with their default setting.

#### ST: Spatial trajectory analysis

For Plasma cells, we carried out spatial trajectory analysis using SPATA2 v0.1.0^118^ to visualize the differentiation trajectory from the germinal center light zone to dark zone to Plasma cell zone. We used the *createTrajectories* function to manually define spatial trajectories across germinal centers to plasma cell rich zones to recapitulate their migration pattern. This enabled us to visualize smoothed gene expression throughout the trajectory using *plotTrajectoryHeatmap* function with smooth_span equal to 0.5.

#### ST: Gene signatures

Gene signatures for Plasma Cells were computed using the package UCell v1.2.0.^109^ To extract relevant marker genes from each cell type, we ran Seurat’s FindAllMarkers on subsetted data containing only Plasma Cells to capture differentially expressed genes between them. For each cell type, we removed ribosomal and mitochondrial genes and filtered out those genes expressed in >25% of *other* cells (keeping those with pct.2 < 0.25). We then ranked them in decreasing order by their avg_log2FC and selected the top 25 for each cell type. Lastly, we ran *AddModuleScore_UCell* to compute each module’s score for each spot on the Visium slides.

#### SLOcatoR: Label and coordinate transfer across modalities

To transfer cell type labels and UMAP coordinates across data modalities, we used the following approach:

1. Define the cell type annotation and UMAP coordinates using the transcriptomic data obtained from scRNA-seq and Multiome, as explained above.
2. As references for label transfer, we used labeled data from Multiome for scATAC-seq and labeled data from scRNA-seq for CITE-seq. Before integration, we find assay-specific features and their integration as defined above to mitigate technology-specific biases between scRNA-seq and CITE-seq.
3. For both scRNA-seq/CITE-seq (gene expression) and Multiome/scATAC-seq (chromatin accessibility), we integrate them with Harmony, as explained above.
4. We define the success by assessing the degree of integration in the UMAP obtained from the harmony-corrected principal components.
5. We transfer the label from Multiome to scATAC-seq and from scRNA-seq to CITE-seq using a K-nearest neighbors (KNN) classifier, as implemented in the knn function of the class v7.3-19 package (default k = 5, optimized in the case of Multiome, see above).
6. We transfer the UMAP coordinates from Multiome to scATAC-seq and from scRNA-seq to CITE-seq using KNN regression, as implemented in the knnreg function of the caret v6.0-90 package (k = 5).

This workflow has been implemented in the SLOcatoR package to connect data modalities and annotate unseen transcriptomes and chromatin accessibility profiles from SLO.

#### Differential abundance analysis of CD4 T cell subsets between young adults and children

To test for age-dependent compositional shifts in CD4 T cells, we focused on samples from the four young adults (BCLL-20-T, BCLL-21-T, BCLL-22-T, BCLL-28-T) and six children (BCLL-8-T, BCLL-9-T, BCLL-10-T, BCLL-11-T, BCLL-12-T, BCLL-13-T) that shared the same indication for tonsillectomy (i.e. tonsillitis), were profiled with the same technology (scRNA-seq), and were processed uniformly (i.e. fresh samples); thus reducing the effect of confounders. We next counted the number of cells for each donor and CD4 T cell subset. We performed the differential abundance analysis with scCODA v0.1.9,^98^ because it considers the compositionality of the data and successfully controls false discovery rate (FDR). ScCODA sets a reference cell type that the model assumes to be constant in both conditions. In this setting, we set the parameter “reference_cell_type” of the function CompositionalAnalysis (formula = “age_group”) to “T-helper”, because we observed that the proportions of this cell type do not change between both age groups. This function sets up the compositional model of scCODA. We ran inference on this model with the sample_hmc method (default parameters) and set the FDR to 0.1 (set_fdr function). Finally, we considered as significant the changes in cell types that were labeled as “True” by the credible_effects function.

#### SLOcatoR: integration of discovery and validation cohorts

The validation cohort consisted of four tonsils from Newcastle Upon Tyne Hospitals NHS Foundation Trust (Newcastle, United Kingdom) and three from Hospital Clinic (Barcelona, Spain; see above). All seven were profiled with 3’ scRNA-seq, and two from Barcelona additionally with Multiome. We ran cellranger, cellranger-arc, and hashtag demultiplexing as described above to be consistent with the analysis of the discovery cohort. Similarly, we filtered out cells and genes following the same principles. We ran the function computeDoubletDensity (dims = 30, subset.row = VariableFeatures(seurat)) from scDblFinder v1.12.0 to obtain a doublet score for each cell, because scDblFinder is the recommended doublet detection method by the most recent best practices.^147^^,^^148^ We filtered out cells based on an outlier doublet score, or clusters that had a very high doublet score as compared to other clusters. We excluded libraries coming from frozen samples from Newcastle because we observed a biased proportion of cell types as compared with the fresh libraries from the same tonsils. After merging all three datasets (scRNA-seq Barcelona, Multiome Barcelona, scRNA-seq Newcastle), we merged them an integrated them with the discovery cohort using SLOcatoR functions as described above, finding variable genes for all combinations of cohort (discovery, validation) and technology (scRNA-seq, Multiome). We then transfer the annotation_level_1 label (i.e. NBC_MBC, GCBC, PC, etc.) from the discovery cohort (reference) to the validation cohort (query). We then repeated the same process with every cell compartment (e.g myeloid cells), discarding lingering clusters of doublets in the process.

#### Data visualization

Different visualization strategies were applied throughout the study to ensure the correct capture and interpretation of the data. In Figure 1B, we plotted all cells with an annotation probability > 0.6. Because interleukin genes are expressed at low levels, we used the Nebulosa v1.5.0 R package^32^ to recover signals. Similarly, we observed that certain ADT in the CITE-seq data were expressed at low levels. To visualize subtle signals in the UMAP plots, we set the order parameter to “TRUE” in the *FeaturePlot* function of Seurat, which plots cells in order of expression. This approach was applied for proteins CD103, CD54, CD161 and CD56 in Figure 3. Likewise, we applied the same parameter to show TF activity in UMAPs throughout the figures. On the other hand, we observed several ADT that had high levels of background noise. For these, we excluded the first and the last percentile when projecting their expression in the UMAPs (Seurat: FeaturePlot, min.cutoff = “q1”/"q5", max.cutoff = “q99”/"q95"). This was applied for all CITE-seq UMAPs in Figure 2C, and for scATAC-seq UMAPs. To represent heatmaps shown throughout the figures, we generated pseudo-bulk expression profiles for each cluster with the *AverageExpression* function (slot = data, default parameters) from Seurat. Subsequently, each row is scaled from 0 to 1 and, finally, the resulting matrix is visualized with *pheatmap*2 function from the pheatmap2 R package. To visualize heatmaps with additional boxplot and/or barplot annotations, we used the Heatmap function from the ComplexHeatmap v2.14.0 package.^121^ Barplot and boxplot annotation were added with the anno_barplot and anno_boxplot functions, respectively.

#### HCATonsilData

HCATonsilData is a BioConductor data package developed following the vignette available here. Briefly, the deposited Seurat objects available in Zenodo were downloaded using zenodo_get. Subsequently, we saved the independent data slots as separate H5File:HDF5 or RDS files, which we later uploaded and stored in a provided Bioconductor Microsoft Azure Data Lakes. HCATonsilData uses ExperimentHub to query and download those data slots, which are then assembled and returned to the user as a SingleCellExperiment object. Finally, HCATonsilData implements a “updateAnnotation” function that allows users to propose new cell type/state annotations using GitHub issues.

#### MCL analysis

To show how the tonsil atlas can provide insights into the cell-of-origin of B cell lymphomas, we excluded the accessibility data and focused solely on the expression data derived from Multiome dataset collected from two MCL patients (M102 and M413). We focused exclusively on the CD19+ fraction. Since both donors shared a similar distribution of library size and number of detected genes but differed on the percentage of mitochondrial expression, we used the same cutoff for the former two metrics and a patient-specific for the latter. Thus, we discarded cells with fewer than 900 UMI, fewer than 700 detected genes, or a percentage of mitochondrial expression greater than 27.5% (M102) or 20% (M413). We normalized, found variable genes, scaled data, ran PCA and UMAP as discussed above for each patient independently (30 PCs for UMAP). We calculated a doublet score using the function computeDoubletDensity (dims = 30, k = 20) of the scDblFinder v1.12.0 package.^148^ After embedding cells in a KNN graph (Seurat: FindNeighbors, 30 PCs) and applying Louvain clustering on that graph (Seurat: FindClusters), we discarded clusters of doublets that had: high doublet score, lacked specific markers, and had a disproportionately high library size. In addition, we discarded one cluster per donor that expressed markers of T cells (e.g. *CD2*, *TRBC1*). Since we removed an axis of variation (doublets, T cells), we rerun the aforementioned pipeline (from HVG to KNN graph). From our infercnv analysis (see below), we noticed that the main driver of variance of MCL intratumoral heterogeneity for both donors was a specific set of subclonal copy number alterations (CNA), including loss of chromosome Y (chr Y). We verified the loss of chr Y by plotting the expression of genes encoded in this chromosome (*UTY*, *KDM5D*, *DDX3Y*, *USP9Y*, *ZFY*, *EIF1AY*). We converted the expression of these genes into a per-cell score (AddModuleScore, default parameters) that allowed us to classify cells into chrY+ and chrY- based on the bimodal distribution of this score. We then clustered cells at low resolution to capture the main genetically different C1 and C2 clusters. We found subclusters within each major C1 and C2 clusters with FindSubCluster function (graph.name = "RNA_snn", adjusting the resolution to find the optimal one). Finally, we found markers (Seurat: FindAllMarkers, only.pos = TRUE, logfc.threshold = 0.75) and annotated clusters to a particular cell state using a combination of marker genes and CNA information.

#### MCL analysis: infercnv

To infer CNA from scRNA-seq, we ran inferCNV v.1.16.0 for each patient separately. We used the non-tumoral B cells from each sample as references. We initialized an ‘infercnv’ object (CreateInfercnvObject) using the raw expression counts and the gene-ordering file.

## Supplemental information

Document S1. Figures S1–S7

Data S1. Glossary of 121 cell types and states of the tonsil atlas, related to STAR Methods

Table S1. Patient metadata, related to Figure 1

Table S2. Quality control metrics per technology, related to Figure 1

Table S3. Differentially expressed genes per cell type, related to Figure 2

Table S4. Gene regulatory network outputs for relevant cell types, related to Figure 2

Table S5. scATAC motif analysis for relevant cell types, related to Figure 3

Table S6. Experimental reagents, related to STAR Methods

Document S2. Article plus supplemental information
