# Supplementary material for: An atlas of cells in the human tonsil
Source: Immunity. 2024 Feb 13;57(2):379–399.e18. doi: 10.1016/j.immuni.2024.01.006 (PMC10869140; doi:10.1016/j.immuni.2024.01.006)
Supplement: Document S1. Figures S1–S7 [file mmc1.pdf]

Immunity, Volume *57*

Supplemental information

An atlas of cells in the human tonsil

Ramon Massoni-Badosa, Sergio Aguilar-Fernández, Juan C. Nieto, Paula Soler-Vila, Marc Elosua-Bayes, Domenica Marchese, Marta Kulis, Amaia Vilas-Zornoza, Marco Matteo Bühler, Sonal Rashmi, Clara Alsinet, Ginevra Caratù, Catia Moutinho, Sara Ruiz, Patricia Lorden, Giulia Lunazzi, Dolors Colomer, Gerard Frigola, Will Blevins, Lucia Romero-Rivero, Víctor Jiménez-Martínez, Anna Vidal, Judith Mateos-Jaimez, Alba Maiques-Diaz, Sara Ovejero, Jérôme Moreaux, Sara Palomino, David Gomez-Cabrero, Xabier Agirre, Marc A. Weniger, Hamish W. King, Lucy C. Garner, Federico Marini, Francisco Javier Cervera-Paz, Peter M. Baptista, Isabel Vilaseca, Cecilia Rosales, Silvia Ruiz-Gaspà, Benjamin Talks, Keval Sidhpura, Anna Pascual-Reguant, Anja E. Hauser, Muzlifah Haniffa, Felipe Prosper, Ralf Küppers, Ivo Glynne Gut, Elias Campo, José Ignacio Martin-Subero, and Holger Heyn

Supplementary Figures

Supplementary Figure Legends (S1-S7)

Supplementary Figures (S1-S7)

Figure S1. A single-cell multiomic atlas of human tonsillar cells. ***Related to Figure 1.*** (**A,G**) UMAP projection of unintegrated (*left*) and harmony-based integrated single-cell datasets (*right*) splitted and colored by data modality [scRNA-seq and multiome (A), scATAC-seq and multiome (G) and external dataset (King *et al.* (A)] (**B, H**) Boxplot comparing local inverse Simpson's Index (LISI) across confounders of integrated and unintegrated scRNA-seq (B) and scATAC-seq (H) datasets. (**C**) UMAP projection of the King *et al.* dataset colored by the original publication. **(D)** UMAP projection of the tonsillar cells of the discovery cohort colored by the main 9 cellular compartments identified: Naive and Memory B cells (NBC_MBC), Germinal center B cells (GCBC), plasma cells (PC), CD4 T cells, cytotoxic (CD8 T cells, NK, ILC, double negative T cells (DN), myeloid cells (DC, macrophages, monocytes, granulocytes, mast cells), follicular dendritic cells (FDC), epithelial cells and plasmacytoid dendritic cells (PDC). **(E)** UMAP projection of tonsillar cells colored by cell cycle phase; *left*: S Score and *right*: G2M Score. **(F)** Pie chart representing the distribution of preB and preT cells across donors. For each donor, the number and percentage of cells (out of the total preB and of the total preT) is indicated. (M: male; F: female). **(I)** UMAP projection of the prediction score inferred for CITE-seq (*left*) and scATAC-seq (*right*) after the label and coordinate transfer.

Figure S2. CD4 T follicular and non-follicular cell fate decision in the human tonsil. ***Related to Figure 2.*** (**A**) Dotplot showing the average expression of markers for Naive, CM pre-non-Tfh and CM Pre Tfh. Dot size reflects the percentage of cells in a cluster expressing each gene and the color the average expression level. (**B**) UMAP projection colored by PRDM1 and BCL6 gene expression and TF-activity gen- (red) and region (green) based obtained with SCENIC+ (STAR methods). (**C**) Dotplot showing the average expression (color) and percentage of cells expressing (size) each of the top 20 markers that described the main CD4 T subpopulations identified. **(D)** Boxplots of BCL6 accessibility values considering the gene body and 2,000 bp upstream (*top*) and the predicted distal cis-regulatoy region using Cicero as a potential enhancer (*bottom*). **(E)** Genomic snapshot of accessibility at BCL6 and distal enhancer locus across T cell clusters from KIng *et al.* **(F)** Violinplot of CD28 (*left*) and CD29 (*right*) protein expression across CD4 T cell subsets. **(G)** UMAPs projection colored by the estimated Nebulosa density expression (STAR Methods) for key interleukin and chemokine receptors. **(H)** Violinplot showing the effector Treg (eTreg, *top*) and circulating Tfr (cTfr, *bottom*) gene expression signatures from Wing JB, *et al.* computed using UCell R package for Treg subtypes.

Figure S3. Landscape of CD8 and innate lymphoid cells in the human tonsil. ***Related to Figure 3.*** (**A**) Heatmap showing the average expression of the top markers for CD8 T cells and innate lymphoid cells (ILC). **(B)** Violinplot showing the protein expression of seven representative markers for different subpopulations in CD8 T and ILC. **(C)** Violinplot showing the motif activity of TBX21 across the different subpopulations in CD8 T and ILC. **(D-G)** Immunofluorescence overlays of markers acquired by multiplex histology in one tissue section from a representative tonsil. Each image depicts the same field of view (FOV) of 665 x 665 µm, sequentially stained with the depicted fluorescence-labelled antibodies. Images contain 2048 x 2048 pixels and are generated using an inverted wide-field fluorescence microscope with a 20x objective, a lateral resolution of 325 nm and an axial resolution above 5 µm. The thin dotted line represents the B cell follicle and the thick dotted line represents the epithelial layer. **(D)** CD20 (cyan) depicts the B cell follicle, CD3 (yellow) marks the T cell zone and pancytokeratin (PCK; magenta) stains the epithelium**. (E)** The endothelial marker CD34 (green) and the myofibroblast marker smooth muscle actin (SMA; red) depict the vessels, and vimentin (blue) marks the fibroblast-dense areas. **(F)** Granzyme A (GzmA; magenta), Eomes (yellow) and CD8 (magenta) represent cytotoxic T cells. **(G)** CD49a (cyan), CD69 (magenta) and CD103 (yellow) mark tissue resident cells. **(C-D)** White outlined squares represent areas of interest, shown as enlargements and single channel images. RM CD8 T cells (CD103+CD69+CD49a+/-) are shown as white arrows and are preferentially localized within the epithelium and in the connective tissue septum lining the tonsillar crypts, but not within the B cell follicles and rarely deep within the T cell zone. (**H**) UMAP projection of CD4 T, CD8 T, NK, ILC and DN in the CITE-seq dataset. (**I**) Protein expression of CD3, CD4 and CD8 across major lymphocyte populations (CITE-seq dataset). (**J**) In silico gating of major lymphocyte populations based on CD4 and CD8 protein expression. (**K**) Distribution of gated groups across each major population. (**L**) FACS validation of DN T cells in tonsils.

Figure S4. B cell activation and GC dynamics. *Related to Figure 4.* (**A**) Dotplot showing marker expression for NBC and MBC subpopulations. (**B**) Dotplot showing marker expression per GCBC subpopulations. Dot size reflects the percentage of cells in a cluster expressing each gene and the color the average expression level. (**C, D**) UMAP projection of GCBC cells colored by cell cycle phase (C) or GCBC annotation (D) and splitted by cell cycle correction strategy. (**E**) Local inverse Simpson's Index (LISI) for both annotation [top, related with (D)] and cell cycle phase [bottom, related with (E)]. (**F**) UMAP projection of NFKB signature computed using *UCell* R package with seven genes (*NFKBIA*, *NFKBID*, *NFKB1*, *NFKB2*, *REL*, *RELA*, *RELB*) for NBC and MBC. (**G**) UMAP projection of *BATF* expression in NBC and MBC.

Figure S5. Plasma cell differentiation and cell identity regulation in human tonsils. *Related to Figure 5.* (**A**) PC BCR analysis. Top: CITE-seq/BCR cells projected in scRNA-seq UMAP coordinates. Cluster labels and coordinates are transferred via KNN classification and regression respectively. Bottom: Barplot representation of PC clonality. Blue, clonal expansion when >= 3 cells had identical CDR3 sequence; Green, two cells share identical sequence; Orange, cells with distinct CDR3 sequence. (**B**) Proliferative cells (PB committed, Transitional PB, PB) differentiation analysis. Top: UMAP projection of proliferative cells colored by cell cycle phase. Bottom: Barplot representation of PC-phenotypic markers expression across S and G2M cells from PB cluster (most representative proliferative cluster). (**C**) UMAPs projection of the expression of Ig and MBC genes (BANK1, CELF2, TXNIP). (**D**) Violin Plot of the Endoplasmic Reticulum (ER) signature across PC scRNA-seq clusters. Signature was obtained using the *UCell* R package with 70 genes obtained from the DAVID KEGG pathway analysis: Protein processing in endoplasmic reticulum. (**E**) Cell type proportions deconvoluted using SPOTlight (see Methods). (**F**) Spatially defined trajectories on H&E stained images from BCLL-10-T (left) and BCLL-12-T (right) patients and heatmap showing smoothed expression changes for specific genes across defined trajectories. BCLL-10-T trajectory starts in a LZ zone and includes an interfollicular zone. (**G**) Correlation plot showing co-localization of cell types on the Visium slides, plot for slide BCLL-10-T (top) and for all slides combined (bottom). (**H**) UMAP projection of the activity (AUCell score) of VDR and CREB3 TFs. (**I**) Barplot of SIX5 expression in different subpopulations from peripheral blood scRNA-seq data from Hao Yuhan *et al.*. (**J**) Boxplot of PC phenotypic markers, SIX5 and SIX5 predicted target in different populations from bone marrow scRNA-seq data from Hay Stuart B *et al.*. Link http://www.altanalyze.org/ICGS/HCA/Viewer.php

Figure S6. Epithelial cells in the human tonsils. *Related to Figure 6.* (**A**) UMAP projection of epithelial tonsillar cells colored and numbered by scRNA-seq clusters. (**B**) Dotplot showing the average expression of the top markers that described the main clusters identified in the epithelial compartment. Dot size reflects the percentage of cells in a cluster expressing each gene and the color the average expression level. **(C)** Cell type proportions of populations of interest within each spot on slide BCLL-2-T along with an H&E image of the tissue slice. Cell type deconvolution was carried out with SPOTlight. We deconvoluted the spots using epithelial cell subpopulation annotations along with the general annotation of other cell types ensuring we captured the biological signal of all our cell types. (**D**) UMAP projection of follicular dendritic cells (FDC) and other cells from mesenchymal origin colored and numbered by scRNA-seq clusters. (**E**) Dotplot showing the average expression of the top markers that described the main clusters identified. **(F)** MAGIC-normalized gene expression of genes of interest on slide BCLL-10-T. (**G**) Dotplot showing the average expression of the top markers that described the main clusters identified in the DC (*left*) and aDC (*right*) compartment. Dot size reflects the percentage of cells in a cluster expressing each gene and the color the average expression level. **(H)** Boxplot showing the proliferation signatures (S.Score and G2M.Score) for DC1 precursor and DC1 mature cells. **(I)** Violinplot showing seven signatures computed using UCell R package for aDC subsets. **(J)** Stacked barplot showing the proportion of cells *(y-axis)* detected in each donor (*x-axis*) for each subpopulation. **(K)** Dotplot showing the average expression of three markers (C1QA, MMP12, SELENOP) across all defined cell types and states in the tonsil atlas.

Figure S7. Confirmation of presence, annotation and markers of tonsillar cell types using a validation cohort and Analysis of two MCL samples in the light of the tonsil atlas. ***Related to Figure 7.*** (**A**, **C**, **E**, **G; I**) UMAP projection of reference (left, discovery cohort) and query (right, validation cohort) for naive and memory B cells [NBC/MBC, (A)], germinal center B cells [GCBC, (C)], plasma cells [PC, (E)], CD8 T (G) and myeloid cells (I). (**B**, **D**, **F**, **H, J**) Heatmap showing scaled mean marker expression for NBC/MBC (B), GCBC (D), PC (F), CD8 T (H) and myeloid cells (J). Boxplots represent the annotation confidence for each cluster. (**K**) UMAP projection the expression of six genes encoded in chromosome Y for M102 (top) and M413 (bottom). (**L**) Distribution and UMAP projection of a chrY expression signature derived from the six genes shown in (A) for M102 (left) and M413 (right). Vertical dashed line shows the cutoff used to classify cells in chrY+/-. (**M**) UMAP projection of the annotated clusters for M413. (**N**) Dotplot showing the average expression (color) and percentage of expressing cells (size) of the top markers that described the main clusters identified in for M413. (**O**, **P**) Heatmaps showing the copy number alterations (CNA) inferred from transcriptomics data with infercnv for M102 (O) and M413 (P).

Figure S1

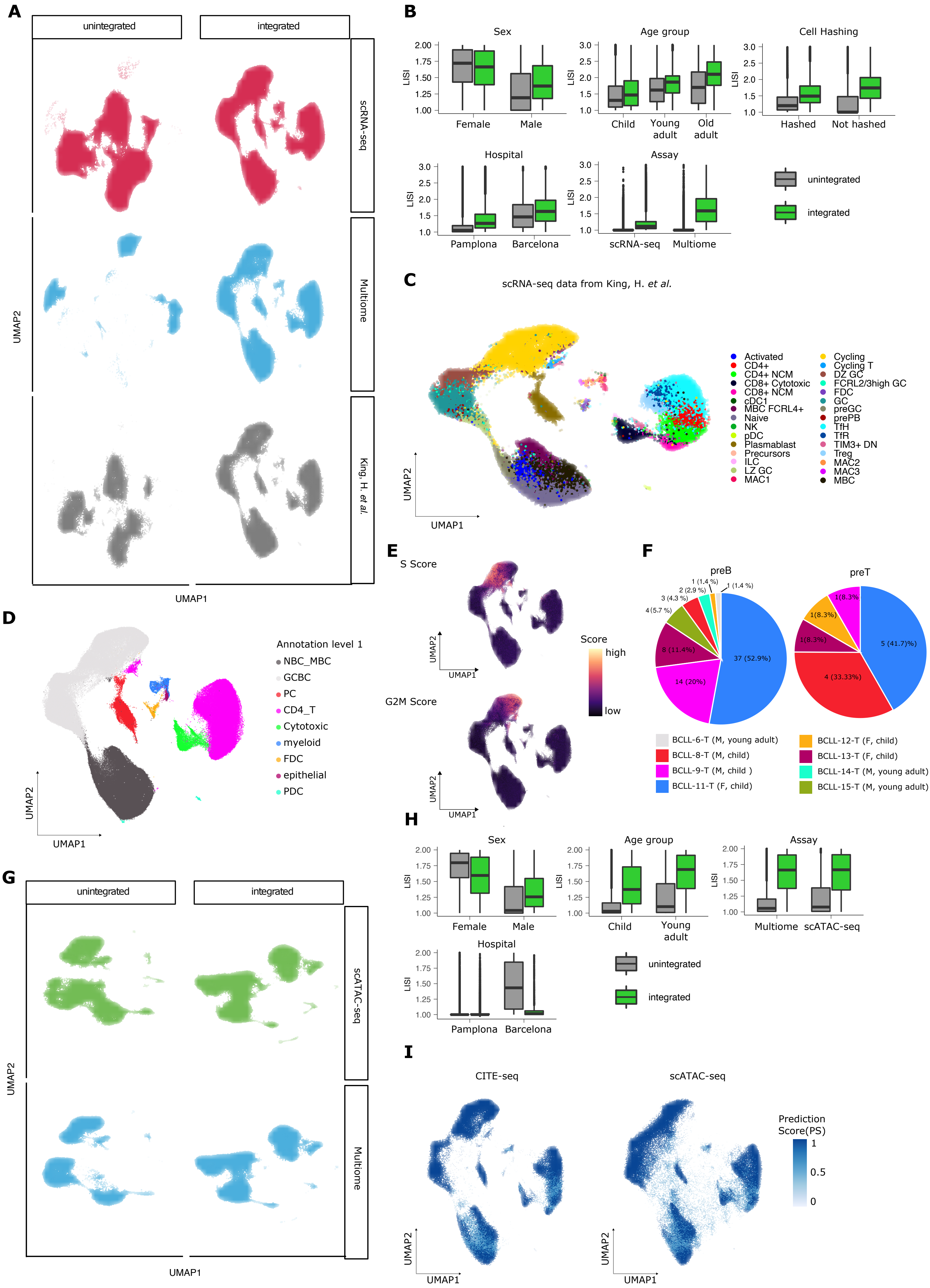

A
unintegrated
integrated
scRNA-seq
Multiome
King, H. et al.
UMAP2
UMAP1
B
Sex
Female
Male
Age group
Child
Young adult
Old adult
Cell Hashing
Hashed
Not hashed
Hospital
Pamplona
Barcelona
Assay
scRNA-seq
Multiome
LISI
unintegrated
integrated
C
scRNA-seq data from King, H. et al.
Activated
CD4+
CD4+ NCM
CD8+ Cytotoxic
CD8+ NCM
cDC1
MBC FCRL4+
Naive
NK
pDC
Plasmablast
Precursors
ILC
LZ GC
MAC1
Cycling
Cycling T
DZ GC
FCRL2/3high GC
FDC
GC
preGC
prePB
TfH
TfR
TIM3+ DN
Treg
MAC2
MAC3
MBC
UMAP2
UMAP1
D
Annotation level 1
NBC_MBC
GCBC
PC
CD4_T
Cytotoxic
myeloid
FDC
epithelial
PDC
UMAP2
UMAP1
E
S Score
G2M Score
Score
high
low
UMAP2
UMAP1
F
preB
37 (52.9%)
14 (20%)
8 (11.4%)
4 (5.7 %)
3 (4.3 %)
2 (2.9 %)
1 (1.4 %)
1 (1.4 %)
preT
5 (41.7%)
4 (33.33%)
1(8.3%)
1(8.3%)
1(8.3%)
BCLL-6-T (M, young adult)
BCLL-8-T (M, child)
BCLL-9-T (M, child)
BCLL-11-T (F, child)
BCLL-12-T (F, child)
BCLL-13-T (F, child)
BCLL-14-T (M, young adult)
BCLL-15-T (M, young adult)
G
unintegrated
integrated
scATAC-seq
Multiome
UMAP2
UMAP1
H
Sex
Female
Male
Age group
Child
Young adult
Assay
Multiome
scATAC-seq
Hospital
Pamplona
Barcelona
LISI
unintegrated
integrated
I
CITE-seq
scATAC-seq
Prediction Score(PS)
1
0.5
0
UMAP2
UMAP1

Figure S2

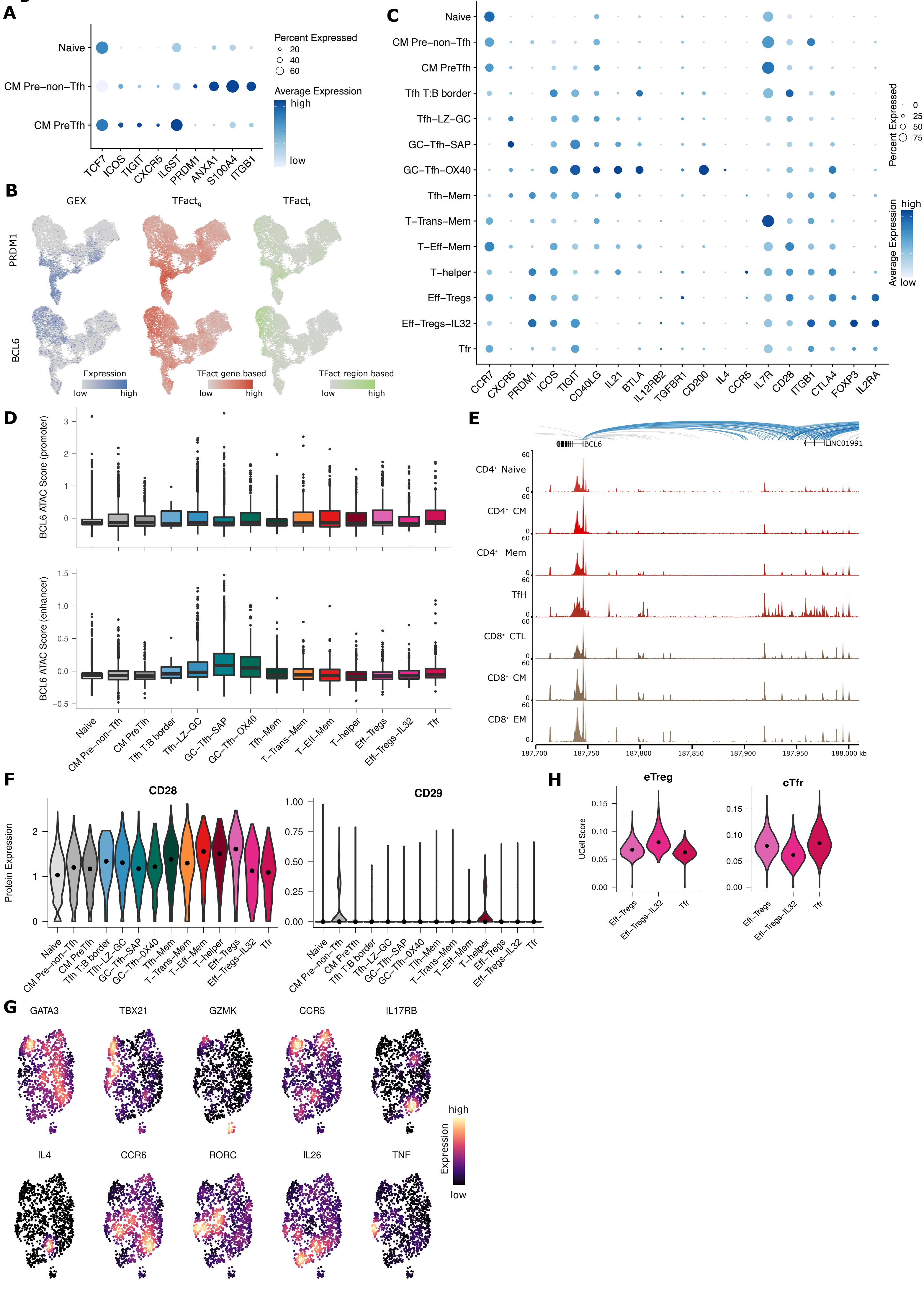
A
Naive
CM Pre-non-Tfh
CM PreTfh
TCF7
ICOS
TIGIT
CXCR5
IL6ST
PRDM1
ANXA1
S100A4
ITGB1
Percent Expressed
Average Expression
high
low
B
GEX
PRDM1
BCL6
Expression
TFact gene based
TFact region based
C
Naive
CM Pre-non-Tfh
CM PreTfh
Tfh T:B border
Tfh-LZ-GC
GC-Tfh-SAP
GC-Tfh-OX40
Tfh-Mem
T-Trans-Mem
T-Eff-Mem
T-helper
Eff-Tregs
Eff-Tregs-IL32
Tfr
CCR7
CXCR5
PRDM1
ICOS
TIGIT
CD40LG
IL21
BTLA
IL12RB2
TGFBR1
CD200
IL4
CCR5
IL7R
CD28
ITGB1
CTLA4
FOXP3
IL2RA
D
BCL6 ATAC Score (promoter)
BCL6 ATAC Score (enhancer)
E
BCL6
LINC01991
CD4+ Naive
CD4+ CM
CD4+ Mem
TfH
CD8+ CTL
CD8+ CM
CD8+ EM
187,700
187,750
187,800
187,850
187,900
187,950
188,000 kb
F
CD28
CD29
Protein Expression
G
GATA3
TBX21
GZMK
CCR5
IL17RB
IL4
CCR6
RORC
IL26
TNF
Expression
H
eTreg
cTfr
UCell Score
Eff-Tregs
Eff-Tregs-IL32
Tfr

Figure S3

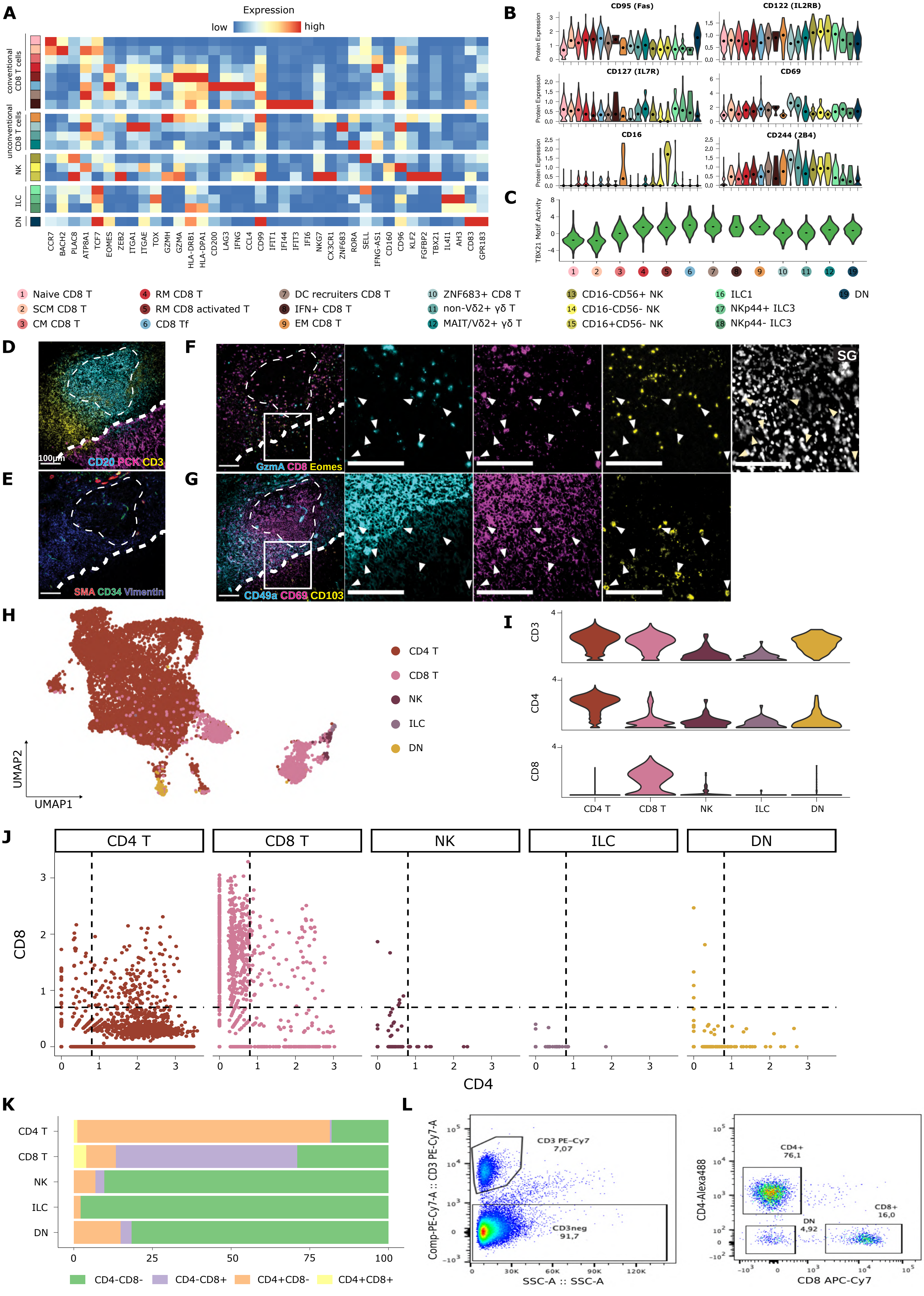

A
Expression
low
high
conventional CD8 T cells
unconventional CD8 T cells
NK
ILC
DN
CCR7 BACH2 PLAC8 ATP8A1 TCF7 EOMES ZEB2 ITGA1 ITGAE TOX GZMH GZMA HLA-DRB1 HLA-DPA1 CD200 LAG3 IFNG CCL4 CD99 IFIT1 IFI44 IFIT3 IFI6 NKG7 CX3CR1 ZNF683 RORA SELL IFNG-AS1 CD160 CD96 KLF2 FGFBP2 TBX21 IL411 AH3 CD83 GPR183
B
CD95 (Fas)
CD122 (IL2RB)
CD127 (IL7R)
CD69
CD16
CD244 (2B4)
Protein Expression
C
TBX21 Motif Activity
1 Naive CD8 T
2 SCM CD8 T
3 CM CD8 T
4 RM CD8 T
5 RM CD8 activated T
6 CD8 Tf
7 DC recruiters CD8 T
8 IFN+ CD8 T
9 EM CD8 T
10 ZNF683+ CD8 T
11 non-Vδ2+ γδ T
12 MAIT/Vδ2+ γδ T
13 CD16-CD56+ NK
14 CD16-CD56- NK
15 CD16+CD56- NK
16 ILC1
17 NKp44+ ILC3
18 NKp44- ILC3
19 DN
D
100µm
CD20 PCK CD3
E
SMA CD34 Vimentin
F
GzmA CD8 Eomes
SG
G
CD49a CD69 CD103
H
UMAP2
UMAP1
CD4 T
CD8 T
NK
ILC
DN
I
CD3
CD4
CD8
J
CD8
CD4
K
CD4-CD8-
CD4-CD8+
CD4+CD8-
CD4+CD8+
L
Comp-PE-Cy7-A :: CD3 PE-Cy7-A
SSC-A :: SSC-A
CD3 PE-Cy7
7,07
CD3neg
91,7
CD4-Alexa488
CD8 APC-Cy7
CD4+
76,1
CD8+
16,0
DN
4,92

Figure S4

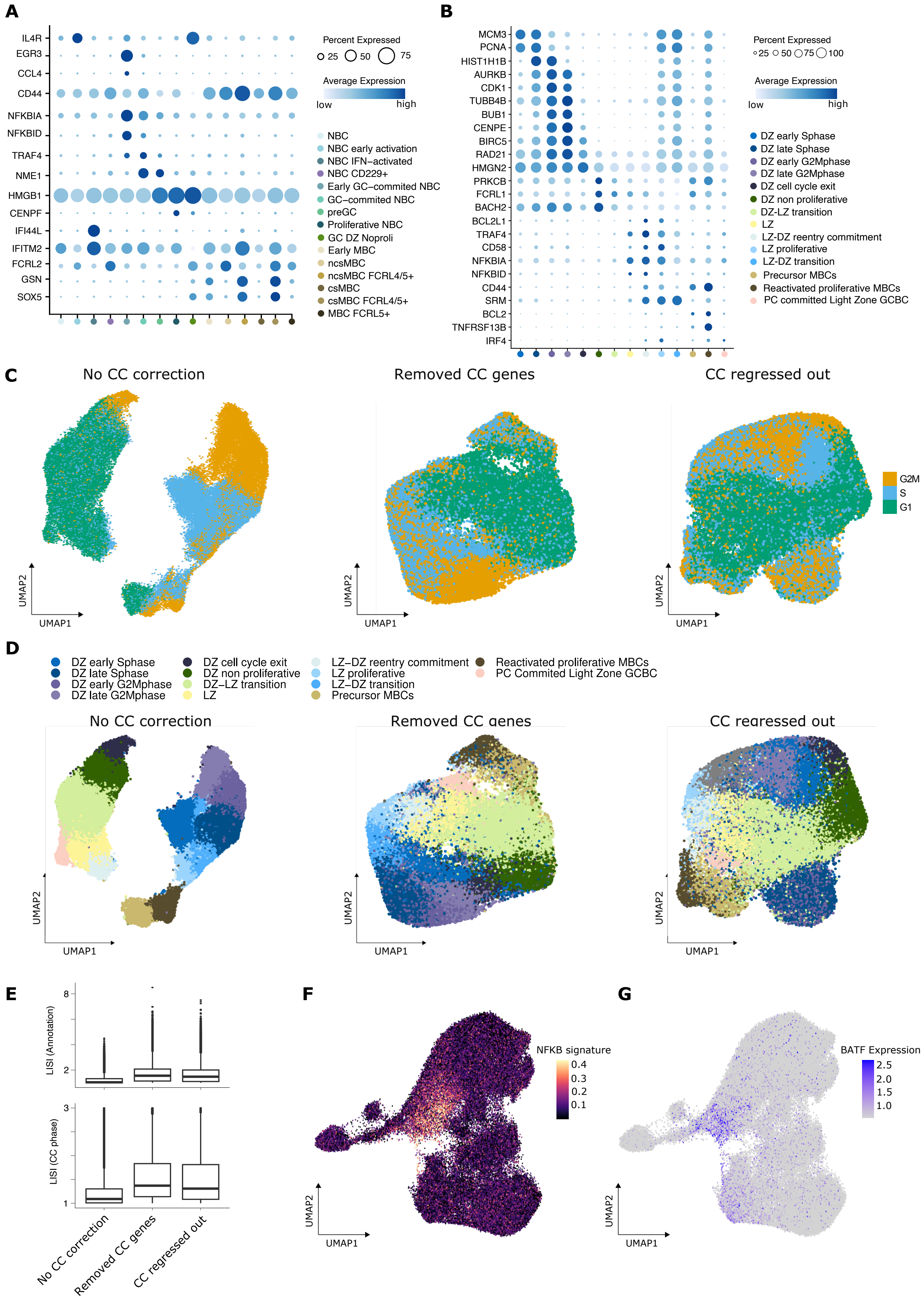
A
IL4R
EGR3
CCL4
CD44
NFKBIA
NFKBID
TRAF4
NME1
HMGB1
CENPF
IFI44L
IFITM2
FCRL2
GSN
SOX5
Percent Expressed
25 50 75
Average Expression
low high
NBC
NBC early activation
NBC IFN–activated
NBC CD229+
Early GC–commited NBC
GC–commited NBC
preGC
Proliferative NBC
GC DZ Noproli
Early MBC
ncsMBC
ncsMBC FCRL4/5+
csMBC
csMBC FCRL4/5+
MBC FCRL5+
B
MCM3
PCNA
HIST1H1B
AURKB
CDK1
TUBB4B
BUB1
CENPE
BIRC5
RAD21
HMGN2
PRKCB
FCRL1
BACH2
BCL2L1
TRAF4
CD58
NFKBIA
NFKBID
CD44
SRM
BCL2
TNFRSF13B
IRF4
Percent Expressed
25 50 75 100
Average Expression
low high
DZ early Sphase
DZ late Sphase
DZ early G2Mphase
DZ late G2Mphase
DZ cell cycle exit
DZ non proliferative
DZ-LZ transition
LZ
LZ-DZ reentry commitment
LZ proliferative
LZ-DZ transition
Precursor MBCs
Reactivated proliferative MBCs
PC committed Light Zone GCBC
C
No CC correction
Removed CC genes
CC regressed out
G2M
S
G1
UMAP2
UMAP1
D
DZ early Sphase
DZ late Sphase
DZ early G2Mphase
DZ late G2Mphase
DZ cell cycle exit
DZ non proliferative
DZ–LZ transition
LZ
LZ–DZ reentry commitment
LZ proliferative
LZ–DZ transition
Precursor MBCs
Reactivated proliferative MBCs
PC Commited Light Zone GCBC
No CC correction
Removed CC genes
CC regressed out
UMAP2
UMAP1
E
LISI (Annotation)
8
2
LISI (CC phase)
3
1
No CC correction
Removed CC genes
CC regressed out
F
NFKB signature
0.4
0.3
0.2
0.1
UMAP2
UMAP1
G
BATF Expression
2.5
2.0
1.5
1.0
UMAP2
UMAP1

Figure S5

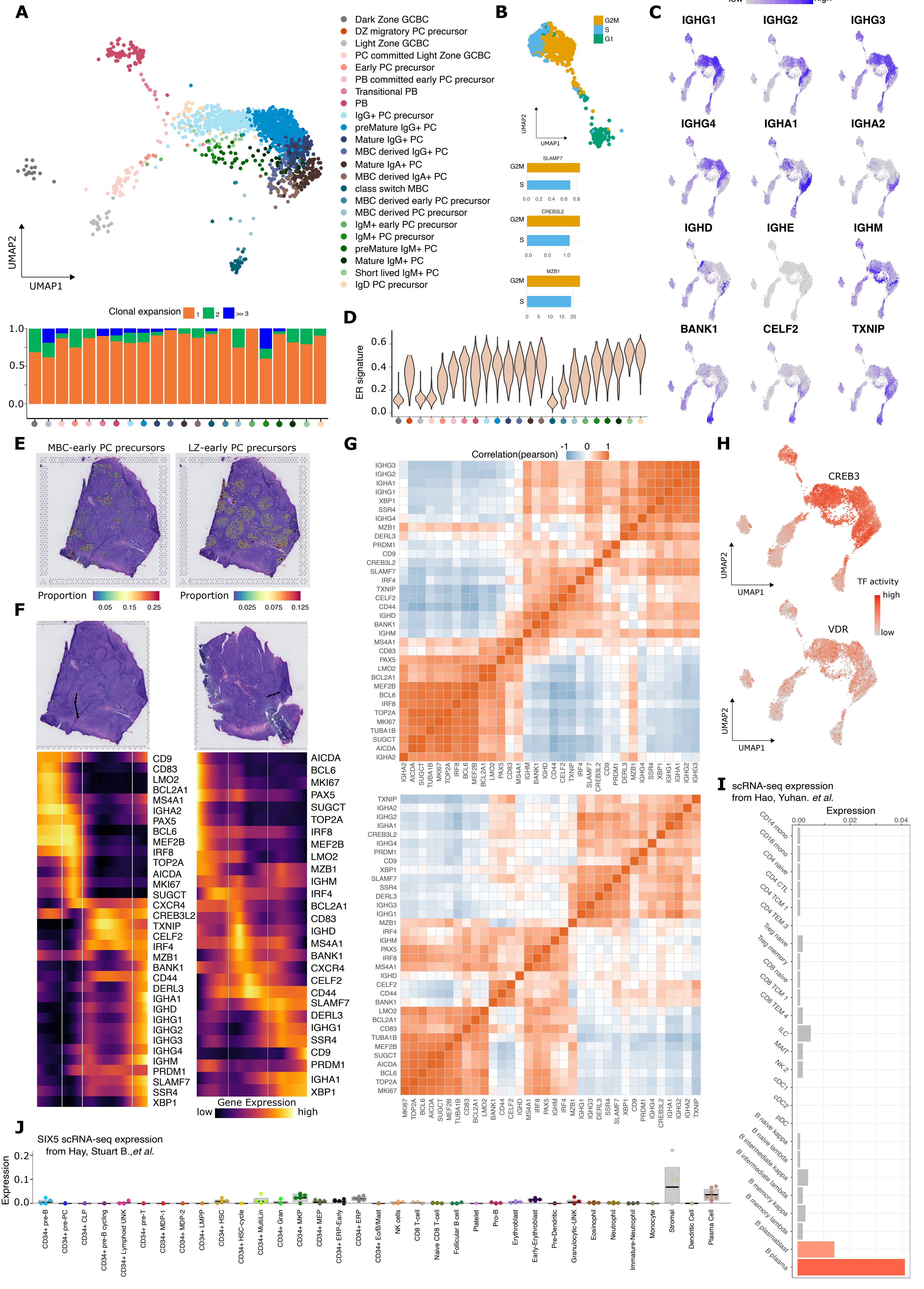
A
Dark Zone GCBC
DZ migratory PC precursor
Light Zone GCBC
PC committed Light Zone GCBC
Early PC precursor
PB committed early PC precursor
Transitional PB
PB
IgG+ PC precursor
preMature IgG+ PC
Mature IgG+ PC
MBC derived IgG+ PC
Mature IgA+ PC
MBC derived IgA+ PC
class switch MBC
MBC derived early PC precursor
MBC derived PC precursor
IgM+ early PC precursor
IgM+ PC precursor
preMature IgM+ PC
Mature IgM+ PC
Short lived IgM+ PC
IgD PC precursor
UMAP1
UMAP2
Clonal expansion 1 2 >=3
B
G2M
S
G1
SLAMF7
CREB3L2
MZB1
C
Expression
low high
IGHG1 IGHG2 IGHG3
IGHG4 IGHA1 IGHA2
IGHD IGHE IGHM
BANK1 CELF2 TXNIP
D
ER signature
E
MBC-early PC precursors
LZ-early PC precursors
Proportion
F
Gene Expression
low high
G
Correlation(pearson)
H
CREB3
VDR
TF activity
high
low
I
scRNA-seq expression
from Hao, Yuhan. et al.
Expression
J
SIX5 scRNA-seq expression
from Hay, Stuart B.,et al.
Expression

Figure S6

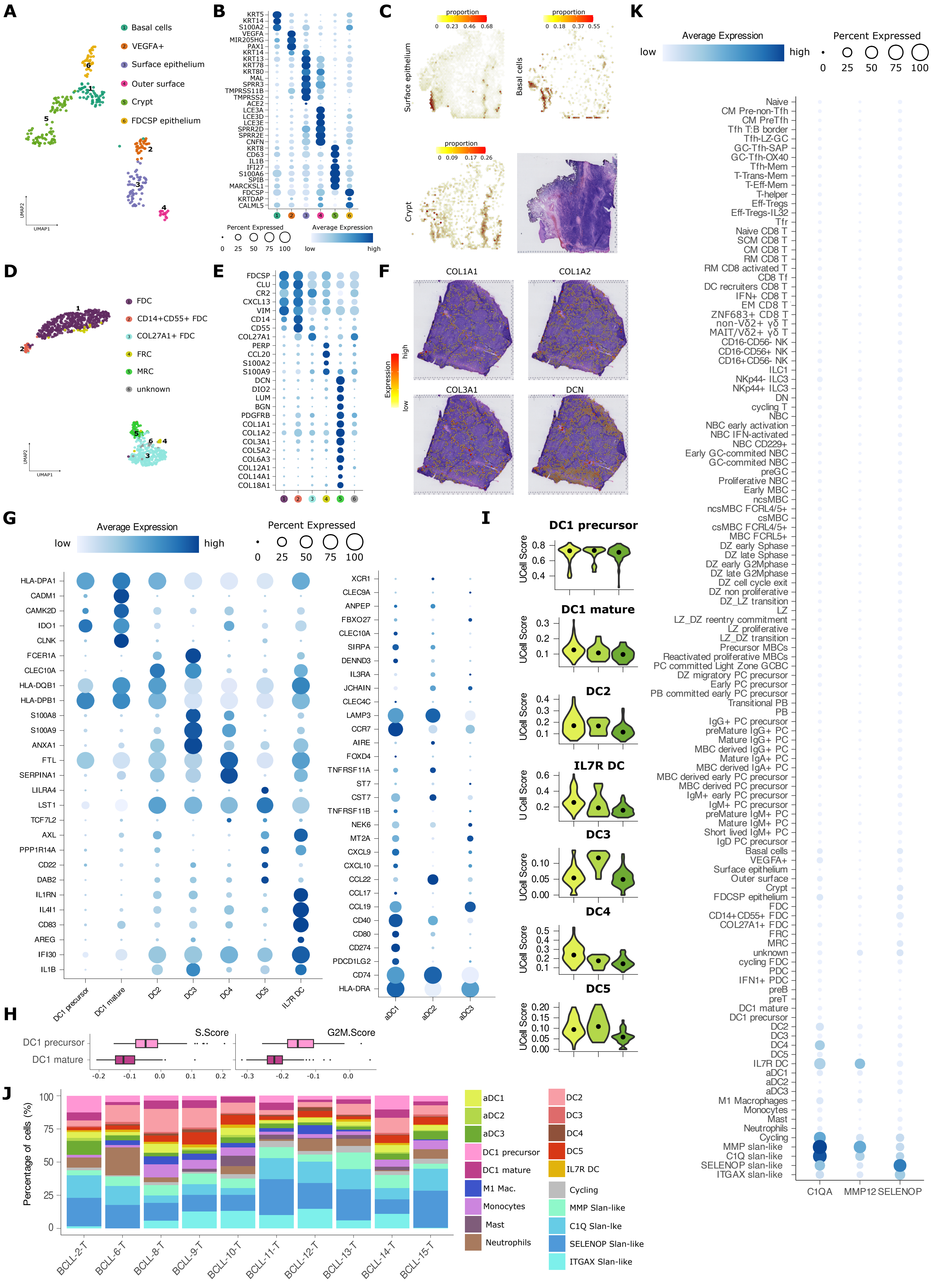

A
Basal cells
VEGFA+
Surface epithelium
Outer surface
Crypt
FDCSP epithelium
B
C
proportion
Surface epithelium
Basal cells
Crypt
K
Average Expression
low
high
Percent Expressed
0 25 50 75 100
D
FDC
CD14+CD55+ FDC
COL27A1+ FDC
FRC
MRC
unknown
E
F
COL1A1
COL1A2
COL3A1
DCN
Expression
high
low
G
Average Expression
Percent Expressed
I
DC1 precursor
DC1 mature
DC2
IL7R DC
DC3
DC4
DC5
UCell Score
H
S.Score
G2M.Score
J
Percentage of cells (%)
aDC1
aDC2
aDC3
DC1 precursor
DC1 mature
M1 Mac.
Monocytes
Mast
Neutrophils
DC2
DC3
DC4
DC5
IL7R DC
Cycling
MMP Slan-like
C1Q Slan-lke
SELENOP Slan-like
ITGAX Slan-like
C1QA
MMP12
SELENOP

Figure S7

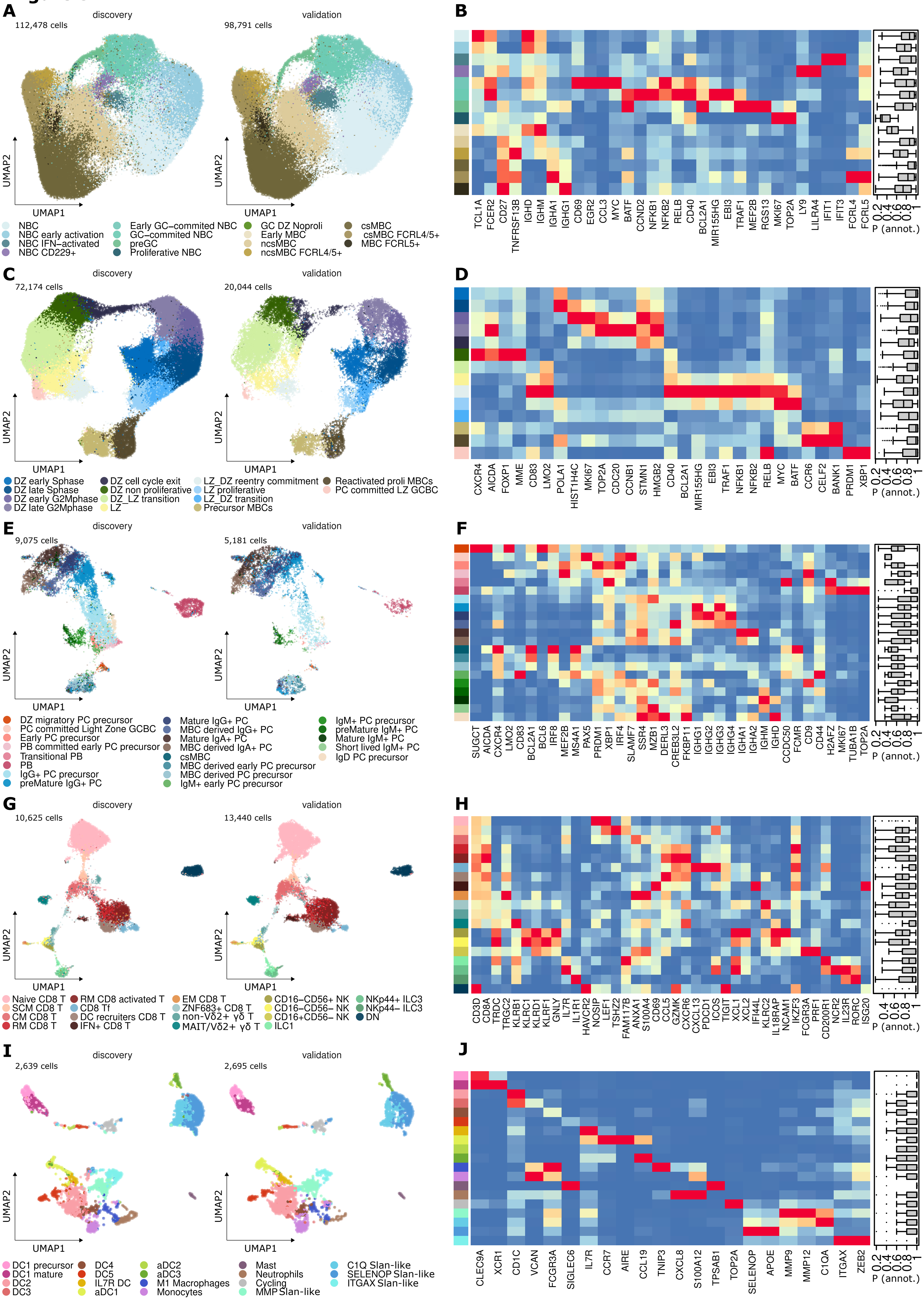
A
discovery
112,478 cells
validation
98,791 cells
UMAP2
UMAP1
NBC
NBC early activation
NBC IFN−activated
NBC CD229+
Early GC−commited NBC
GC−commited NBC
preGC
Proliferative NBC
GC DZ Noproli
Early MBC
ncsMBC
ncsMBC FCRL4/5+
csMBC
csMBC FCRL4/5+
MBC FCRL5+
B
TCL1A
FCER2
CD27
TNFRSF13B
IGHD
IGHM
IGHA1
IGHG1
CD69
EGR2
CCL3
MYC
BATF
CCND2
NFKB1
NFKB2
RELB
CD40
BCL2A1
MIR155HG
EBI3
TRAF1
MEF2B
RGS13
MKI67
TOP2A
LY9
LILRA4
IFIT1
IFIT3
FCRL4
FCRL5
0.2 0.4 0.6 0.8 1
P (annot.)
C
discovery
72,174 cells
validation
20,044 cells
UMAP2
UMAP1
DZ early Sphase
DZ late Sphase
DZ early G2Mphase
DZ late G2Mphase
DZ cell cycle exit
DZ non proliferative
DZ_LZ transition
LZ
LZ_DZ reentry commitment
LZ proliferative
LZ_DZ transition
Precursor MBCs
Reactivated proli MBCs
PC committed LZ GCBC
D
CXCR4
AICDA
FOXP1
MME
CD83
LMO2
POLA1
HIST1H4C
MKI67
TOP2A
CDC20
CCNB1
STMN1
HMGB2
CD40
BCL2A1
MIR155HG
EBI3
TRAF1
NFKB1
NFKB2
RELB
MYC
BATF
CCR6
CELF2
BANK1
PRDM1
XBP1
0.2 0.4 0.6 0.8 1
P (annot.)
E
discovery
9,075 cells
validation
5,181 cells
UMAP2
UMAP1
DZ migratory PC precursor
PC committed Light Zone GCBC
Early PC precursor
PB committed early PC precursor
Transitional PB
PB
IgG+ PC precursor
preMature IgG+ PC
Mature IgG+ PC
MBC derived IgG+ PC
Mature IgA+ PC
MBC derived IgA+ PC
csMBC
MBC derived early PC precursor
MBC derived PC precursor
IgM+ early PC precursor
IgM+ PC precursor
preMature IgM+ PC
Mature IgM+ PC
Short lived IgM+ PC
IgD PC precursor
F
SUGCT
AICDA
CXCR4
LMO2
CD83
BCL2A1
BCL6
IRF8
MEF2B
MS4A1
PAX5
PRDM1
XBP1
IRF4
SLAMF7
SSR4
MZB1
DERL3
CREB3L2
FKBP11
IGHG1
IGHG2
IGHG3
IGHG4
IGHA1
IGHA2
IGHM
IGHD
CCDC50
FCMR
CD9
CD44
H2AFZ
MKI67
TUBA1B
TOP2A
0.2 0.4 0.6 0.8 1
P (annot.)
G
discovery
10,625 cells
validation
13,440 cells
UMAP2
UMAP1
Naive CD8 T
SCM CD8 T
CM CD8 T
RM CD8 T
RM CD8 activated T
CD8 Tf
DC recruiters CD8 T
IFN+ CD8 T
EM CD8 T
ZNF683+ CD8 T
non-Vδ2+ γδ T
MAIT/Vδ2+ γδ T
CD16−CD56+ NK
CD16−CD56− NK
CD16+CD56− NK
ILC1
NKp44+ ILC3
NKp44− ILC3
DN
H
CD3D
CD8A
TRDC
TRGC2
KLRB1
KLRC1
KLRD1
KLRF1
GNLY
IL7R
IL1R1
HAVCR2
NOSIP
LEF1
TSHZ2
FAM117B
ANXA1
S100A4
CD69
CCL5
GZMK
CXCR6
CXCL13
PDCD1
ICOS
TIGIT
XCL1
XCL2
IFI44L
KLRC2
IL18RAP
NCAM1
IKZF3
FCGR3A
PRF1
CD200R1
NCR2
IL23R
RORC
ISG20
0.4 0.6 0.8
P (annot.)
I
discovery
2,639 cells
validation
2,695 cells
UMAP2
UMAP1
DC1 precursor
DC1 mature
DC2
DC3
DC4
DC5
IL7R DC
aDC1
aDC2
aDC3
M1 Macrophages
Monocytes
Mast
Neutrophils
Cycling
MMP Slan-like
C1Q Slan-like
SELENOP Slan-like
ITGAX Slan-like
J
CLEC9A
XCR1
CD1C
VCAN
FCGR3A
SIGLEC6
IL7R
CCR7
AIRE
CCL19
TNIP3
CXCL8
S100A12
TPSAB1
TOP2A
SELENOP
APOE
MMP9
MMP12
C1QA
ITGAX
ZEB2
0.2 0.4 0.6 0.8 1
P (annot.)

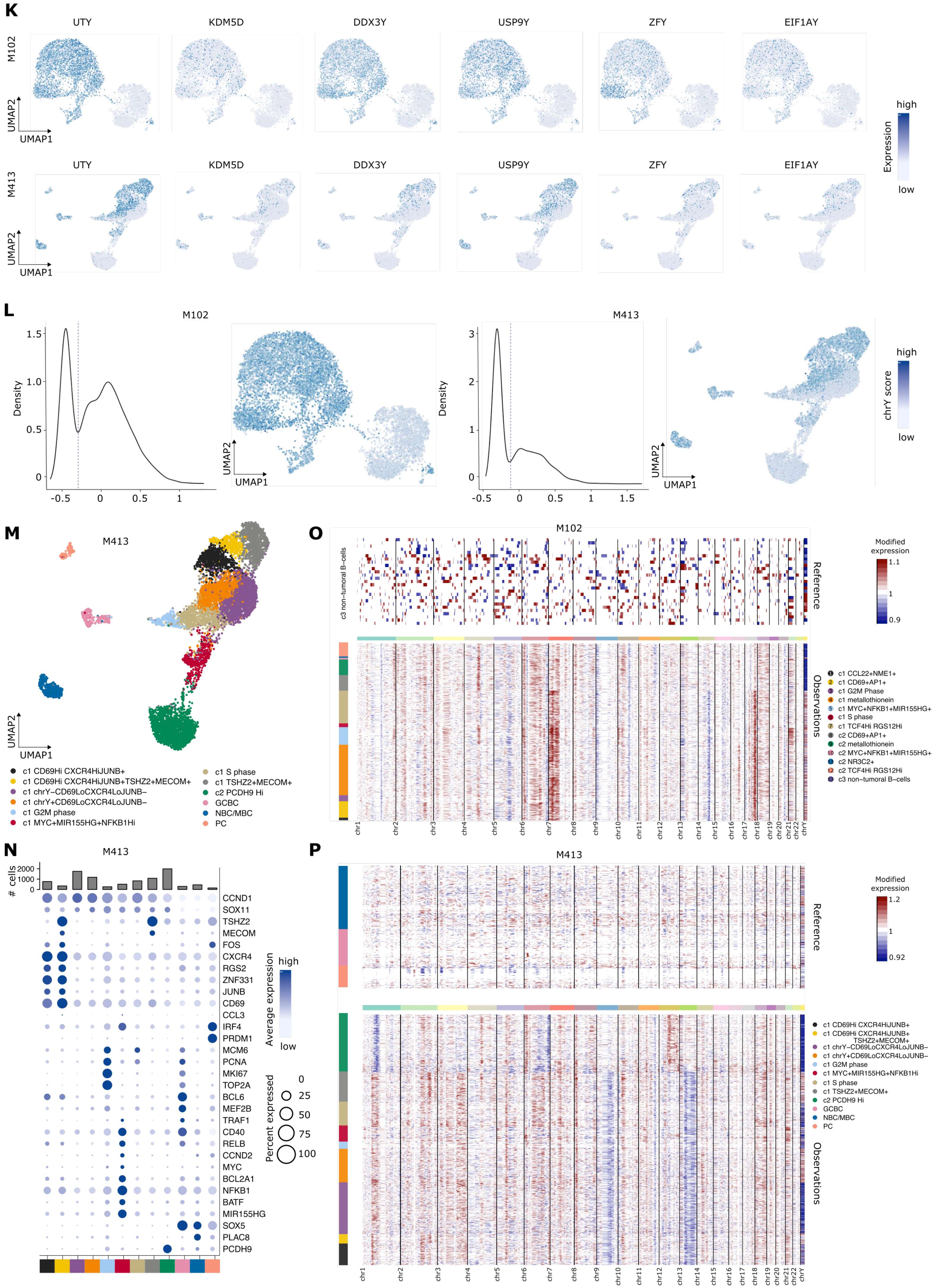
K
UTY
KDM5D
DDX3Y
USP9Y
ZFY
EIF1AY
M102
M413
UMAP1
UMAP2
high
Expression
low
L
M102
M413
Density
chrY score
M
M413
c1 CD69Hi CXCR4HiJUNB+
c1 CD69Hi CXCR4HiJUNB+TSHZ2+MECOM+
c1 chrY−CD69LoCXCR4LoJUNB−
c1 chrY+CD69LoCXCR4LoJUNB−
c1 G2M phase
c1 MYC+MIR155HG+NFKB1Hi
c1 S phase
c1 TSHZ2+MECOM+
c2 PCDH9 Hi
GCBC
NBC/MBC
PC
N
cells
CCND1
SOX11
TSHZ2
MECOM
FOS
CXCR4
RGS2
ZNF331
JUNB
CD69
CCL3
IRF4
PRDM1
MCM6
PCNA
MKI67
TOP2A
BCL6
MEF2B
TRAF1
CD40
RELB
CCND2
MYC
BCL2A1
NFKB1
BATF
MIR155HG
SOX5
PLAC8
PCDH9
Average expression
Percent expressed
O
M102
c3 non−tumoral B−cells
Reference
Observations
Modified expression
c1 CCL22+NME1+
c1 CD69+AP1+
c1 G2M Phase
c1 metallothionein
c1 MYC+NFKB1+MIR155HG+
c1 S phase
c1 TCF4Hi RGS12Hi
c2 CD69+AP1+
c2 metallothionein
c2 MYC+NFKB1+MIR155HG+
c2 NR3C2+
c2 TCF4Hi RGS12Hi
c3 non−tumoral B−cells
P
M413
Modified expression
c1 CD69Hi CXCR4HiJUNB+
c1 CD69Hi CXCR4HiJUNB+ TSHZ2+MECOM+
c1 chrY−CD69LoCXCR4LoJUNB−
c1 chrY+CD69LoCXCR4LoJUNB−
c1 G2M phase
c1 MYC+MIR155HG+NFKB1Hi
c1 S phase
c1 TSHZ2+MECOM+
c2 PCDH9 Hi
GCBC
NBC/MBC
PC
