# Supplementary material for: An atlas of cells in the human tonsil
Source: Immunity. 2024 Feb 13;57(2):379–399.e18. doi: 10.1016/j.immuni.2024.01.006 (PMC10869140; doi:10.1016/j.immuni.2024.01.006)
Supplement: Data S1. Glossary of 121 cell types and states of the tonsil atlas, related to STAR Methods [file mmc2.pdf]

DATA S1: GLOSSARY OF TONSILLAR CELL SUBPOPULATIONS AND STATES

The goal of this glossary is to help readers understand the rationale behind the designation of each cell subpopulation. It is not a detailed description of the multimodal features of each subpopulation but just the information we have used to name each cell type or state. It will be divided into ten sections representing the major cell populations identified at the UMAP level 1, and the references used to annotate them.

1. Plasmacytoid Dendritic Cells and Precursors T and B cells ……………………………….... page 2
2. CD4+ T cells ……………………..……………………....………..………..………..………..… page 3-4
3. CD8+ T cells/innate lymphoid cells ……………………....………..………..………..………. page 5-6
4. Naive/Memory B cells ……………………....………..………..………..…………………….. page 7-9
5. Germinal center B cells ……………………....………..………..………..……………………. page 10-12
6. Plasma cells ……………………....………..………..………..……………………………….. page 13-16
7. Myeloid cells ……………………....………..………..………..……………………...……………. page 17-18
8. Epithelial cells ……………………....………..………..………..…………………...…………. page 19
9. Mesenchymal cells/follicular dendritic cells ……………………....……………..…...…………. page 20
10. References …………………………………………………………....……………..…...…………. page 21-24

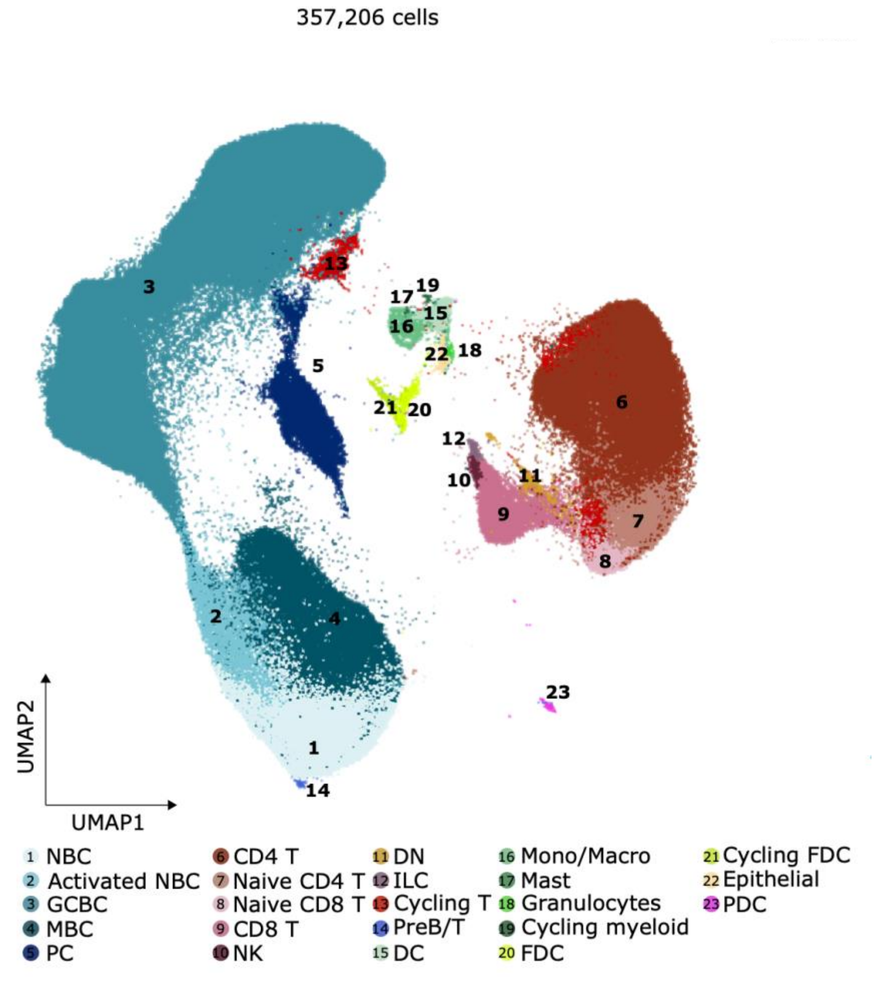

Plasmacytoid Dendritic Cells and Precursors T and B cells

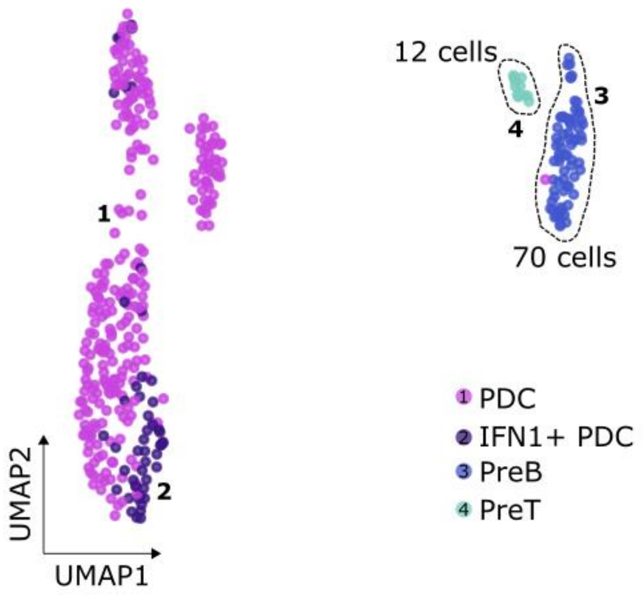

PDC: pDCs were previously identified by the standard protein marker CD123 (IL3RA). This cluster had higher expression of *IRF8, IL3RA* and *LILRA4* (Villani et al., 2017).

IFN1+ PDC: The second pDC subpopulation was identified by standard protein marker CD123 and as IFN-producing cells. This cluster had higher expression of *IRF8, IL3RA, LILRA4* and *IFI44, IFI6* (Park et al., 2020; Villani et al., 2017).

PreB: Precursor B cells were identified by the expression of *CD79B, CD19, PAX5, RAG1/2.* (Strauchen and Miller, 2003).

PreT: Precursor T cells in tonsils expressed: *CD3G, CD8A, RAG1, DNTT, CD1A, CD1B, CD1C, CD1E* (McClory et al., 2012).

CD4+ T CELLS

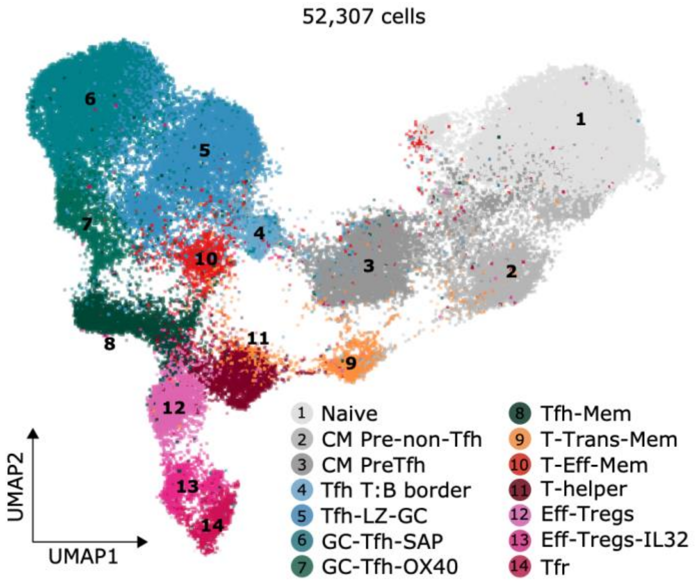

Naive CD4 T cells: Naïve cell is a CD4 T cell that recently migrates from the thymus towards lymph nodes. Naïve CD4 T cells expressed lymph node homing genes such as *CCR7* and naïve-associated genes: *SELL*, *LEF1, BACH2* and *NOSIP*. Also, they were negative for *PRDM1* and *BCL6* and were CD62L+ and CD45RA+ (Cano-Gamez et al., 2020).

Central Memory Pre-non-Tfh (CM Pre-non-Tfh): After antigen encounter T cells acquire memory and effector functions maintaining the lymph node homing. CM showed an upregulation of *IL7R* compared to naïve T cells, and higher expression of PRDM1*, PASK, ANXA1* and *S100A4*. They had an intermediate expression of CCR7 and higher expression of CD28 confirming their migratory capacity to secondary lymph nodes and their memory profile based on their effector function. This cluster was CD45RO+, CD28+ and CD29+(Cano-Gamez et al., 2020; Nicolet et al., 2021).

CM Pre- Tfh: Early Tfh cell differentiation is regulated by IL-6 and ICOS. This cluster had a similar CM profile, but differential expression of follicular precursors: *TCF7, IL6ST* and *CXCR5*, and up-regulation of follicular genes such as *ICOS, PDCD1* and *TIGIT* (Qi, 2016; Sallusto et al., 2004).

Tfh T:B border: After antigen recognition, CD4 T cells migrate toward the border of the follicle activating B cells. This cluster showed a downregulation on the expression of *CCR7* and up-regulation of *BCL6*, *TOX*, *CXCR5 and BTLA*. The expression of *ICOS* and *CD40LG* confirmed the interaction with B cells at the border of the follicle (Crotty, 2019).

Tfh LZ GC: Tfh cells provide IL-21 and CD40 signals required for B cell proliferation and differentiation toward both GC and extrafollicular fates. This cluster expressed *IL21* and *CD40LG*. Also, the initial up-regulation of follicular markers as *PDCD1*. ATAC-seq analysis confirms the activity of *BCL6* in this cluster as a follicular T cell subpopulation (Crotty, 2014).

GC Tfh SAP: Terminally differentiated Tfh cells in GC acquired functions depending on the expression of certain markers. This cluster expressed *SH2D1A* (SAP, top marker) and specific genes and transcription factors associated with GC function: *CXCL13, PDCD1* and *POU2AF1*. SAP was demonstrated to be essential in CD4 T cells for GC responses and the generation of memory B cells and long-lived plasma cells (Booth et al., 2011; Crotty, 2011)

GC Tfh OX40: OX40 expression (*TNFRSF4*) on Tfh cells play a role in the maturation and Ig secretion of B cells and plasma cells during GC reaction (Fu et al., 2021). This cluster expressed higher levels of *TNFRSF4, CD200, IL21* and *IL4 canonical molecules of GC Tfh.* IL-21, and IL-4 are major ‘‘help’’ molecules produced by GC Tfh cells to keep GC B cells alive and induce their proliferation (Crotty, 2011). *CXCL13, IL-21* and *IL- 4* are also potent inducers of IgG1 class switch recombination for human B cells (Choi and Crotty, 2021).

Tfh Mem: Upon leaving a GC, the Tfh cell acquires a less activated, less polarized Tfh phenotype and can upregulate *KLRB1* and develop into resting memory Tfh cells. This cluster had the expression of follicular markers *PDCD1, MAF,* and higher expression of memory markers such as *KLRB1, ITGB1,* and *CD28* (Crotty, 2014).

T Trans-Mem: Transitional memory T cells appear to be more differentiated than TCM cells but not as fully differentiated as TEM cells in terms of phenotype. This cluster had higher levels of *IL7R, CD28, CCR6, RORA* and lower *KLRB1*. This cluster was CD62L-, CD45RO+ and CD28+ (Mahnke et al., 2013).

T-Eff-Mem: Following antigen clearance, effector cells die while a small pool of T cells ultimately develops into long-lived effector memory T cells. This cluster was positive for *KLRB1, HECW2*, and *EGLN3.* The phenotype of this cluster included CD45R0+, CD161+ and CD62L- (Cano-Gamez et al., 2020; Mahnke et al., 2013).

T-helper: T helper cells play a critical role in defending infections by coordinating immune responses. CD4 T cells execute their functions mainly in peripheral tissues and various lymphoid organs. CD4 T cells receive signals from the environment and differentiate into specific subsets of T helper cells to efficiently recognize a broad range of antigens (Zhu and Zhu, 2020).

Th1: Th1 cells induce responses against intracellular pathogens by activating macrophages. This cluster expressed *CXCR3, TBX21, GZMK* and *CCR5,* main markers of Th1 cells (Krueger et al., 2021).

Th2: Th2 cells are involved in type 2 responses to fight infections by parasites such as helminths, as well as the recruitment of eosinophils, basophils, and mast cells to the sites of infections. This cluster had higher expression of *CCR4, GATA3, IL17RB* and *IL4* (Cano-Gamez et al., 2020; Stark et al., 2019).

Th17: Th17 cells mediate responses for the clearance of extracellular bacteria and fungi by orchestrating sustained neutrophil recruitment. This cluster had higher expression of *CCR6, RORC, IL17A* and *IL17F* (Ma et al., 2016).

Th17/Th1: Th17 cells can convert to Th1 cells based on the cytokine environment. This cluster expressed *IFNG*, *IL26* and *IL17A* (Damsker et al., 2010).

Th22: Th22 cells are players in adaptive immune responses and are identified by the production of IL-22. This cluster had higher levels of *IL22, IL10* and *TNF* (Jia and Wu, 2014).

Eff-Tregs: Effector T regulatory cells play an important role in maintaining the homeostasis in tissues and control immune responses. This cluster is the classical Treg subpopulation that expresses IL2RA, CTLA4, IL1R1, IL1R2 and IL10 and specific transcription factors FOXP3, MAF, IKZF1 and IKZF3 and it is CD25+ and CD28+ (Du et al., 2021).

Eff-Tregs-IL32: This cluster had a similar profile of Eff-Tregs, it showed a distinct transcriptional signature, with overexpression of several suppressive effectors, but also proinflammatory molecules like IL32. This cluster had as top genes: *IL32, FOXP3, CTLA4, IL2RA* and *IKZF2* (Galván-Peña et al., 2021).

Tfr: T-follicular regulatory cells in the tonsils are CD25-. These cells down-regulate effector Treg markers (*IL2RA*, *FOXP3*, *CTLA4*). This cluster expressed high levels of *FCRL3*, *CLNK*, *LEF1*, *TCF7*, *RBMS3*, *SESN3*, and *PDE3B*. The top marker *FCRL3* can bind secretory IgA to suppress the Tfr inhibitory function. TCF7 and LEF1 are essential for Tfr development in mice (Wing et al., 2017). (Agarwal et al., 2020; Wing et al., 2017; Yang et al., 2019)

CD8+ T CELLS AND INNATE LYMPHOID CELLS

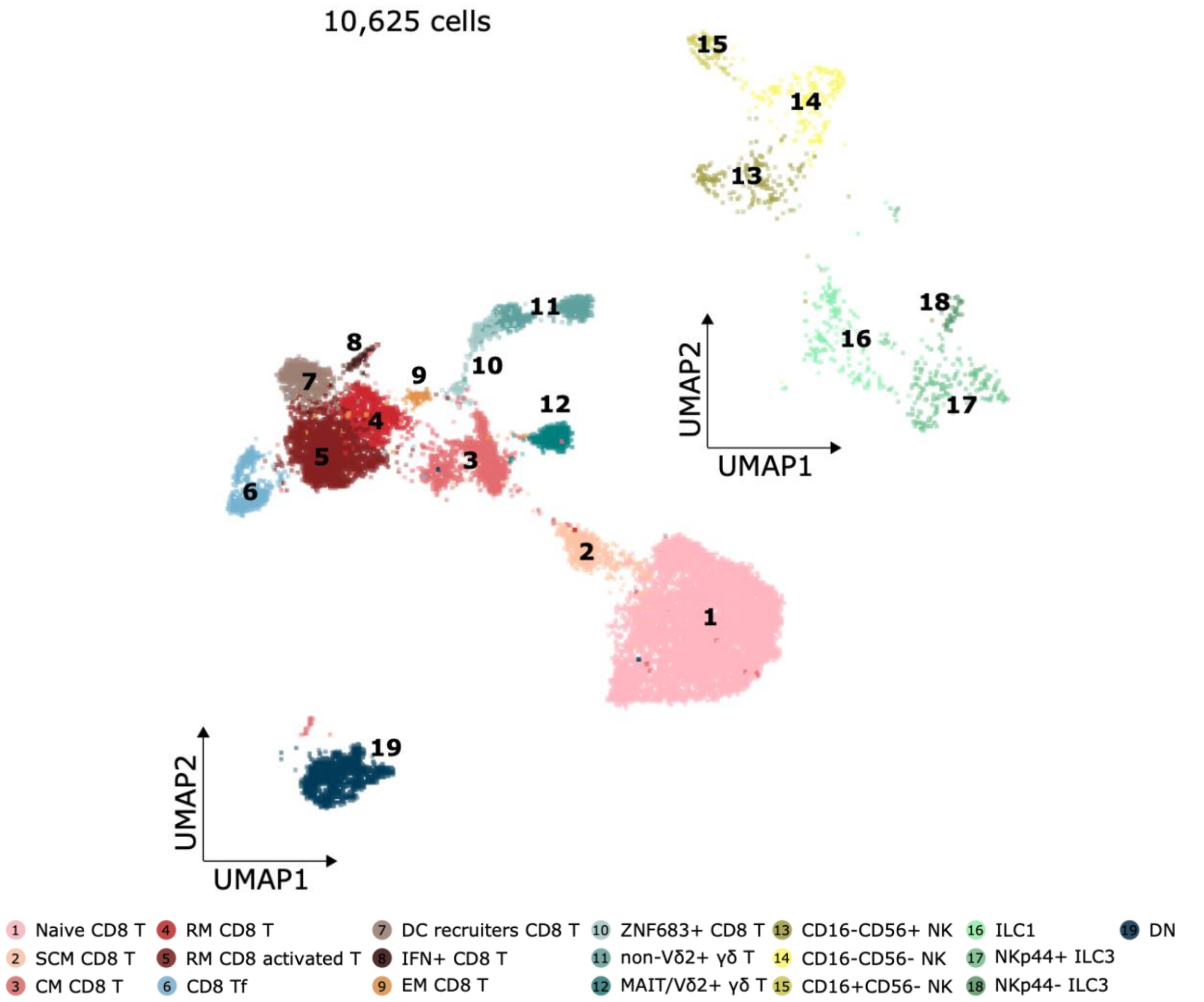

Naïve CD8: Naïve cell is a CD8 T cell that recently migrates from the thymus towards lymph nodes. This cluster expressed *BACH2, LEF1, NOSIP* and *CCR7*. Also, this cluster was CD45RA+ (Mahnke et al., 2013).

SCM CD8 T: Stem-cell memory T cells self-renew and generate long-lived central memory T cells and short-lived effector memory T cells. Differential expression analysis reveals these genes as the main markers: *FAM117B, TCF7, TSHZ2* and *PLAC8*. This population was CD95+ and CXCR3+ (Galletti et al., 2020, Gattinoni et al., 2011)

CM CD8 T: CM CD8 T cells acquire memory (marked by IL7R+) while maintaining lymph node homing capacity (CCR7+). CM CD8 T cells expressed higher levels of *IL7R* compared to naïve T cells, and higher expression of *ANXA1* and *S100A4*. They had an intermediate expression of CCR7 and a higher expression of *CD28*. This cluster was CD45RO+, and CD28+ (Mahnke et al., 2013).

RM CD8 T: Resident memory T cells (TRM) are essential for immune defense against pathogens and malignancies. The unique identity of TRM cells in tissues is defined by a combination of decreased expression of proteins that facilitate their exit from tissues and increased expression of proteins that enhance their ability to stay in tissues (e.g. CD103 and CD49a). A pool of TRM-poised cells generate RM

CD8 T cells within the tonsil compartment. The main gen markers of this cluster are: *CD69, EOMES, ZEB2, ITGA1* and *TOX,* and CD69+ (Kok et al., 2022).

RM CD8 Activated T: This cluster had a similar profile of RM T cells but it had higher expression of *GZMH, CCL5, GZMA, GZMK, CXCR6, HLA-DRB1, HLA-DPA1* and *CD38.* These markers showed an activated profile of these RM CD8 T cells. Also, It was positive for CD103 and CD54. Intriguingly, this cluster had a lower expression of CD69 compared with the other RM subset. (Kok et al., 2022; Walsh et al., 2019)

CD8 Tf: CD8+ T cells located within the lymphoid follicle are follicular CD8+ T cells that play an important role in viral and tumor control, as well as to modulate humoral and T follicular helper cell responses. This cluster expressed high levels of CD200, CXCL13, ICOS, PDCD1 and IFNG, and it was CXCR5+, PD-1+ and ICOS+ (Perdomo-Celis et al., 2017).

DC recruiters CD8 T: CD8+ T cells can orchestrate the local recruitment of tonsil-resident XCR1+ DCs via secretion of the XCL1 chemokine. Functionally, this CD8+ T cell-mediated reorganization of the local DC network allows for the interaction and cooperation of pDCs and XCR1+ DCs. This cluster had as top markers *CD160, CCL5, XCL2, XCL1* and *CCL4*. These markers confirmed their role in the recruitment of DCs (Brewitz et al., 2017).

IFN+ CD8 T: This cluster had an expression of genes related with IFNg responses. The up-regulated genes were *HLA-DRB1, IFIT3, IFIT5* and *IFITM2. (Li et al., 2023; Szabo et al., 2019)*

EM CD8 T: This cluster is CCR7- and expresses high levels of CX3CR1, TBX21 (Gerlach et al., 2016), and multiple cytotoxicity genes, including GZMB, GZMH, GZMA, PRF1, and GNLY. TBX21 and EOMES motifs are highly active in this cluster (scATAC-seq data).

non-Vδ2+ γδ T: γδ T cells are a distinct subgroup of T cells containing T cell receptors (TCR) γ and δ chains. In this cluster we identified a clear signature of gd T cells: *KLRD1, KLRC2, TRGC1, TRGC2* and *TRDC* (Pizzolato et al., 2019).

ZNF683+ CD8 T: This cluster is CD8+, expresses *ZNF683* (HOBIT), *NCAM1* (CD56), *GNLY*, *GZMB*, tissue-resident memory T cell markers (*ITGAE*, *ITGA1*), and multiple NK cell receptors, including *KLRC2* (NKG2C), *KLRC3* (NKG2E), *KLRD1* (CD94), and *KIR2DL4*. A ZNF683+ CD8 T cluster was also reported in the thymus atlas. (Park et al., 2020)

MAIT/Vδ2+ γδ T: This cluster is a mix of MAIT cells (*KLRB1*, *SLC4A10*, *ZBTB16*, *IL23R*, *IL18R1*, *CEPBD*, *CCR6*, *DPP4*) and Vδ2Vγ9+ γδ T cells (*TRDV2*, *TRDC*, *TRGC1*, *TRGC2*, *ZBTB16*, *KLRB1*, *IL18R1*, DPP4) which share a similar phenotype and transcriptional profile *(*Provine et al., 2018; Wragg et al., 2020*).*

CD16-CD56+ NK: These cells have an immunoregulatory function, possess a strong ability to react to cytokine stimulation, are mainly found in secondary lymphoid organs, and have limited cytotoxic capacity. They are the precursors of CD16+CD56- NK cells. This cluster expressed *KLRF1*, *NCAM1*, *SELL, TCF7, GZMK and XCL1*. (Boldt et al., 2014; Pfefferle et al., 2019; Poli et al., 2009)

CD16-CD56dim NK: An intermediate NK cell subtype between precursor (CD16-CD56+) and differentiated NK cells (CD16+CD56-). This cluster expressed *KLRF1*, *XCL1*, and *XCL2*. (Boldt et al., 2014; Pfefferle et al., 2019; Poli et al., 2009)

CD16+CD56- NK: Fully differentiated NK cells with high cytotoxic capacity. This cluster had a high expression of *KLRF1*, *FCGR3A, CX3CR1, TBX21, and PRF1.* (Boldt et al., 2014; Pfefferle et al., 2019; Poli et al., 2009)

ILC1: ILC1 are characterized by their capacity to produce IFNγ and they depend on the transcription factor T-bet. Contrary to NK cells, ILC1 express *IL7R* (CD127) and CD200R; and do not express *KLRF1* (NKp80). Accordingly, this cluster expressed high levels of *IL7R*, *CD200R1, CD69* and *ANXA1*; and did not express *KLRF1* (Boldt et al., 2014; Vivier et al., 2018)

NKp44+ ILC3 and NKp44- ILC3: ILC3s depend on RORC for their development and function, and comprise subsets that can be distinguished on the basis of expression of the natural cytotoxicity receptors NKp46 and NKp44. The main cytokine produced by ILC3s is IL-22 and a proportion of ILC3s also produce

IL-17. We identified two subtypes of ILC3s that expressed *RORC* and *IL23R*; and were NKp44 (*NCR2*) positive and negative (Bal et al., 2020; Vivier et al., 2018)

DN: Double negative T cells express the αβ T cell receptor but do not express CD4, CD8, or natural killer cell markers. This cluster had a profile of inflammatory and activated DN T cells expressing *HLA-DRB1, HLA-DPA1, CD38* and *GPR183* (Wu et al., 2022b).

Cycling T: This cluster is a T cell cluster (CD3+) that had a higher score of cell cycle and had higher expression of cell cycle genes: *STMN1, HIST1H4C, TUBA1B, HMGB2, MKI67, TOP2A* and *TUBB*. As we observed that the proliferative signature was a major driver of variance in scRNA-seq data, which caused (1) cells from different lineages to cluster together and (2) hindered biological heterogeneity in the other cluster. Thus we show this population in Figure 1B, but exclude it from downstream analysis. A cluster of cycling T was also observed in (King et al., 2021).

NAIVE AND MEMORY CELLS

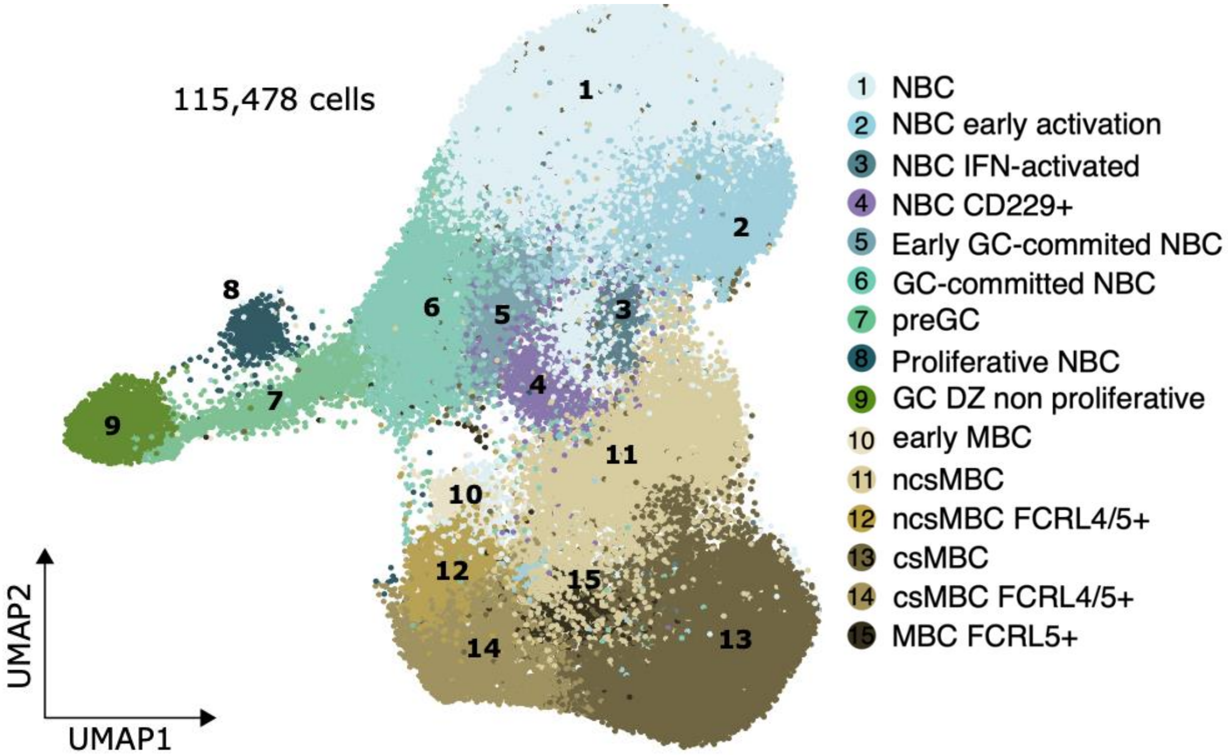

* For the sake of mapping cell transitions, in this analysis we included *DZ non proliferative GCBCs* from the GCBC level 1 cluster. This subpopulation is not described in this section of the glossary but in the GCBC section.

NBC: This is the main cluster of NBCs, with a clear and homogenous expression of FCER2 (CD23), TCLA1, IGHM and IGHD. This cluster seems to represent standard NBC lacking the expression of genes related to cell activation or commitment towards GC reaction. We would like to clarify that we performed a targeted search for two known NBC subpopulations that are expected to be present in tonsillar tissue, i.e. CD5 Mature NBCs and transitional B cells. However, CD5, a key marker to define these subpopulations, could not be detected by scRNA-seq. We also used alternative gene signatures defined by Seifert et al. (Seifert et al., 2012) but we could not detect any particular subcluster. Therefore, although we cannot rule out that CD5 Mature NBCs and transitional B cells are present in our dataset -probably within the standard NBC cluster-, we could not identify them.

NBC early activation: Cells in this cluster show expression of typical NBC markers such as FCER2 (CD23), TCLA1, IGHM and IGHD. This cluster is transcriptionally very similar to standard NBCs, with the exception of a few genes related to B cell activation, such as CD69 and IL4R (Cibrián and Sánchez-Madrid, 2017; Ferrer et al., 2014). Therefore, these cells seem to represent the first step of NBC activation.

NBC INF-activated: Cells in this cluster expressed typical NBC markers such as FCER2 (CD23), TCLA1, IGHM and IGHD. Several genes expressed exclusively by the cells in these clusters are associated with interferon induction, as for example ISG15, the IFIT gene family (i.e. or IFIT1, IFIT2 or IFIT3) family, IFI44, IFI44L and MX1. Thus, this cluster represents NBC cells that have been activated in response to interferon stimulation.

NBC CD229+: Cells in this cluster express typical NBC markers such as FCER2 (CD23), TCLA1, IGHM and IGHD. As compared to the standard NBC cluster, only few genes were differentially expressed. We identified two main markers, related to immune system and B cell biology that were specifically expressed within this cluster, i.e. LY9 (CD229), which has been shown to be highly expressed in marginal zone B cells (Cuenca et al., 2016) and LILRA4 (Cao and Bover, 2010), which actually is more relevant for plasmacytoid dendritic cells. Unfortunately, the lack of literature related to this particular combination of markers precluded us from assigning a biological function to this cluster.

Early GC-committed NBC: Cells in this cluster express typical NBC markers such as FCER2 (CD23), TCLA1, IGHM and IGHD. This cluster is characterized by the expression of very specific genes that seem to correspond to early GC commitment due to the transient MYC upregulation (Dominguez-Sola et al., 2012). For instance, we observed high expression of EGR family (i.e. EGR1, EGR2, EGR3), transcription factors that are rapidly induced upon stimulation of the BCR genes (Gururajan et al., 2008) related to NF-kB pathway (i.e. NFKB1, NFKB2, NFKBIA, NFKBID, RELB) (Burger and Chiorazzi, 2013); or proinflammatory chemokines i.e. CCL3 and CCL4 (Takahashi et al., 2015) . Furthermore, in this cluster we identified high levels of bone-fide activation marker, i.e. CD69 (Cibrián and Sánchez-Madrid, 2017). As this marker was also present at lower levels in the NBC early activation cluster, we hypothesize that these two clusters represent sequential steps of the NBC activation process towards the GC reaction.

GC-committed NBC: Cells in this cluster express typical NBC markers such as FCER2 (CD23), TCLA1, IGHM and IGHD. This cluster seems to follow the Early GC-committed NBCs in the differentiation path between NBC and GCBCs. For instance, one of the main markers of this cluster is CCND2, which is a bona fide downstream target of MYC (a marker for early GC commitment, (Dominguez-Sola et al., 2012). It also maintains the expression of NF-kB pathway related genes. Furthermore, the cells in this cluster express genes associated with signaling pathways related to response to antigens, such as MIR155HG, TRAF1/4, EBI3, and BCL2A1. Finally, we observed upregulation of BATF, a gene known to be involved in the regulation of AID (AICDA) expression and thus antibody diversification in DZ-GCBCs (Ise et al., 2011). Although this cluster lacks any GC-related marker, it seems to represent a cell stage that is already committed to enter the GC reaction.

preGC: Cells in this cluster express typical NBC markers such as FCER2 (CD23), TCLA1, IGHM and IGHD. As compared to the preceding clusters related to NBC activation, within this cluster we could detect the expression of GC-specific genes, such as RGS13 or MEF2B. (King et al., 2021) However, these cells still partially maintain the NBC transcriptome and transcriptional programs started in the previous steps of NBC activation process (i.e. GC-committed NBC) such as the BATF expression. Additionally, this cluster still lacks other GC phenotypic genes, such as AICDA or BCL6. Therefore, we conclude that this subcluster may represent the last step of NBC activation before entering into the DZ and starting a proper GC reaction.

Proliferative NBC: Cells in this cluster express typical NBC markers such as FCER2 (CD23), TCLA1, IGHM and IGHD. This cluster is characterized by a proliferation signature, marked by high expression of MKI67, TOP2A, histone genes and genes related with chromosome segregation. Intriguingly, this cluster does not contain any marker related to GC, i.e. lack of expression of RGS13, MEF2B (the two expressed in preGC cluster), AICDA and BCL6. We therefore discarded that these cells were related to the GC-dependent maturation pathway of NBCs. Instead, we hypothesize that this population may correspond to primary focal reaction upon antigen stimulation (Jacob and Kelsoe, 1992), which leads to rapid generation of antibody-producing cells in a GC-independent manner.

Early MBC: This cluster of the NBC/MBC level 1 cluster is CD27 positive, and therefore, we annotate it as MBC. However, this cluster does not have many specific markers, with the exception of e.g. low-medium levels of MEF2B, a GC marker (King et al., 2021). This might suggest that this cluster represents MBCs freshly generated from the GC reaction, and still contains traces of GC markers such as MEF2B.

ncsMBC: This is one of the main clusters of MBCs, with clear expression of known markers such as CD27 and CD267. It is also characterized by high expression of IGHD and IGHM, and therefore, this cluster is classified as non-class switch MBC (ncsMBCs) (Akkaya et al., 2020).

csMBC: This is one of the main clusters of MBCs, with clear expression of known markers such as CD27 and CD267. It is also characterized by high expression of IGHA1 and IGHG1, and therefore it is classified as class switch MBC (csMBCs) (Akkaya et al., 2020).

ncsMBC FCRL4/5+: This is a subcluster of ncsMBC (positive for CD27, CD267, IGHD and IGHM), characterized by the expression of FCRL4 and FCRL5. This subpopulation is typically found in tonsils but not in peripheral blood or bone marrow. In concordance with the available literature related to FCRL4+ MBCs, this cluster expresses high levels of GSN, SOX5, FGR or HCK, among others (Li et al., 2020).

csMBC FCRL4/5+: This is a subcluster of csMBC (positive for CD27, CD267, IGHA and IGHG), characterized by the expression of FCRL4 and FCRL5. This subpopulation is typically found in tonsils but not in peripheral blood or bone marrow. In concordance with the available literature related to FCRL4+ MBCs, this cluster expresses high levels of GSN, SOX5, FGR or HCK, among others (Li et al., 2020).

MBC FCRL5+: This is a low abundant cluster of MBC cells (positive for CD27 and CD267) that present and intermediate transcriptomic signature between csMBC and csMBC FCRL4+/FCRL5+. These cells express only FCRL5 with the absence of FCRL4. This subpopulation has been described as MBC FCRL5-only, which indeed was shown to express many genes at intermediate level between FCRL4/FCRL5 MBCs and MBC (Li et al., 2020).

GERMINAL CENTER B CELLS

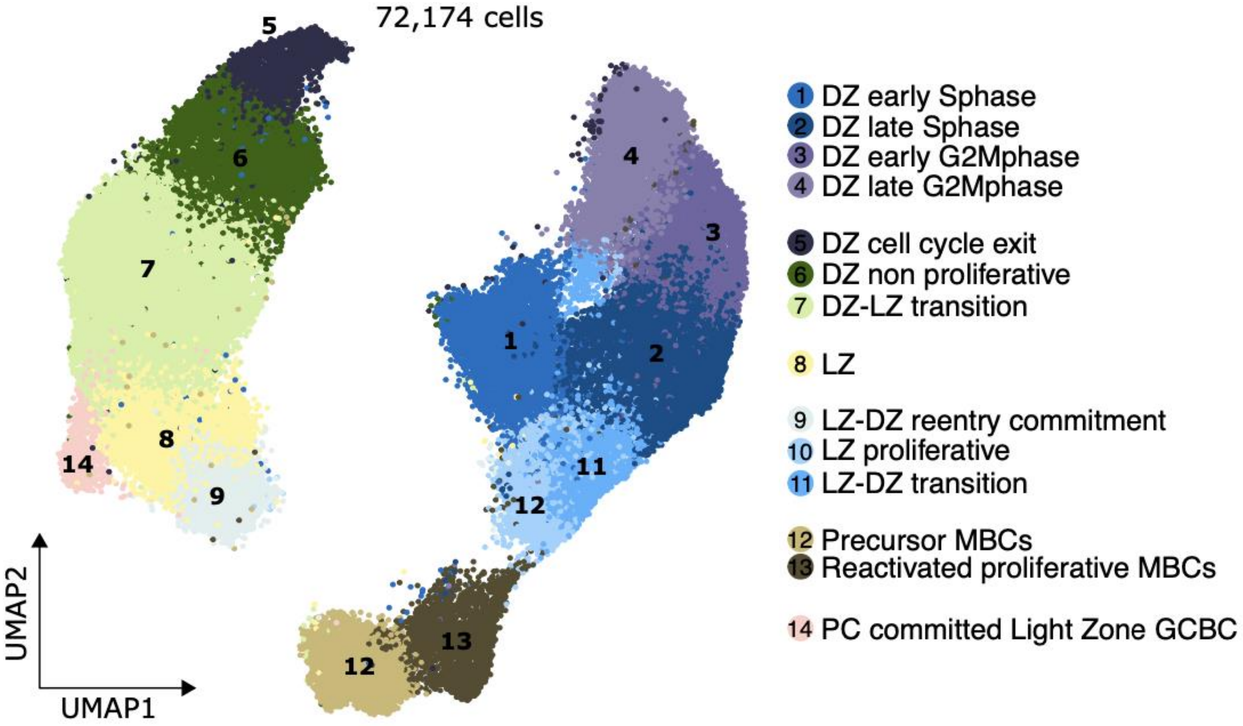

*The *PC committed light zone GCBC* subpopulation will be described in the plasma cell section.

DZ-early S phase: Cluster with high expression of CXCR4 (a marker defining DZ GCB cells) and additional expression of DZ markers, such as FOXP1 and MME, and moderate levels of AICDA. This cluster displays a clear proliferation signature and high S-phase score. Cells express typical cell cycle genes, such as MCM family, POLA1 or PCNA, which are associated with DNA replication and strand elongation as well as G1/S transition GO terms. This cluster lacks expression of histone genes (i.e. HIST1H4C, HIST1H1B, HIST2H2AH, HIST1H3D), which are related to a more advanced S phase. Therefore, the present cluster most likely represents the early steps of the S phase of the cell cycle. (Bar-Joseph et al., 2008; Holmes et al., 2020; King et al., 2021; Milpied et al., 2018).

DZ-late S phase: Cluster with high expression of CXCR4 (a marker defining DZ GCB cells) and additional expression of DZ markers, such as FOXP1 and MME, and moderate levels of AICDA. This cluster displays a clear proliferation signature and high S-phase score. Cells express typical cell cycle genes, such as MCM family, POLA1 or PCNA, which are associated with DNA replication and strand elongation as well as G1/S transition GO terms. This cluster shows upregulation of histone genes (i.e. HIST1H4C, HIST1H1B, HIST2H2AH, HIST1H3D), suggesting that it contains cells associated with the later steps of the S phase. Moreover, the cells within this cluster start expressing the first traces of genes related to more advanced phases of the cell cycle, such as AURKB, TOP2A or MKI67. (Bar-Joseph et al., 2008; Holmes et al., 2020; King et al., 2021; Milpied et al., 2018).

DZ-early G2M phase: Cluster with high expression of CXCR4 (a marker defining DZ GCB cells) and additional expression of DZ markers, such as FOXP1 and MME, and intermediate levels of AICDA. This cluster has a clear G2M phase score. It is characterized by high expression of genes related to spindle organization, chromosome segregation, mitotic cell cycle and division, i.e. BUB1, TOP2A, centromere

proteins family, tubulin genes, AURKA, CDK1 and MKI67. However, cells from this cluster still have high expression of histone genes and do not present the upregulation of cyclin B and CDC20, suggesting that they are in early G2M phase (Bar-Joseph et al., 2008; Holmes et al., 2020; King et al., 2021; Milpied et al., 2018).

DZ-late G2M phase: Cluster with high expression of CXCR4 (a marker defining DZ GCB cells) and additional expression of DZ markers, such as FOXP1 and MME, as well as high expression of AICDA. This cluster has a clear G2M phase score. It is characterized by high expression of genes related to spindle organization, chromosome segregation, mitotic cell cycle and division, i.e. BUB1, TOP2A, centromere proteins family, tubulin genes, AURKA, CDK1 and MKI67. Cells from this cluster start to downregulate the expression of histone genes concomitantly with the upregulation of cyclin B genes and CDC20, indicating full entry into mitosis. (Bar-Joseph et al., 2008; Holmes et al., 2020; King et al., 2021; Milpied et al., 2018).

DZ-cell cycle exit: Cluster with high expression of CXCR4 (a marker defining DZ GCB cells) and additional expression of DZ markers, such as FOXP1 and MME, as well as moderate expression of AICDA. Cells from this cluster start to downregulate the expression levels of cell cycle program genes. For instance, they lack CDK1 but still contain lower levels of CCNB1, CDC20, TOP2A and MKI67 as compared to the cells from preceding DZ-G2M phase clusters. The cells still maintain clear expression of other proliferation-related genes such as HMG family genes, STMN1 or BIRC5. This cluster was not associated with any significant upregulation as compared to the preceding cluster (DZ-late G2M phase). Therefore, we defined this cluster as dark zone cells that have started to exit the cell cycle. (Bar-Joseph et al., 2008; Holmes et al., 2020; King et al., 2021; Milpied et al., 2018)

DZ-non-proliferative: Cluster with high expression of CXCR4 (a marker defining DZ GCB cells) and additional expression of DZ markers, such as FOXP1 and MME, as well as moderate expression of AICDA. Cells from these clusters are clearly non-proliferative, lacking the detectable expression of any cell cycle gene. Therefore, we hypothesize that cells in this cluster, although they maintain the expression of dark zone-related genes, have already finished the cell cycle program and are progressing towards the next maturation stage (Bar-Joseph et al., 2008; Holmes et al., 2020; King et al., 2021; Milpied et al., 2018).

DZ-LZ transition: This relatively large non-proliferative cluster is characterized by rather intermediate or low expression of DZ markers, such as CXCR4. The cells start to express some LZ markers such as CD83 or LMO2, but still are missing other LZ-genes such as BCL2A1 or DUSP2. Furthermore, we could not define clear markers specific and unique for this cluster. From the chromatin perspective, this cluster contained intermediate levels of regions differentially accessible between the DZ and the LZ. Therefore, we believe that these cells represent a transitional subpopulation moving from the DZ to the LZ. (Bar-Joseph et al., 2008; Holmes et al., 2020; King et al., 2021; Milpied et al., 2018)

LZ: Cells in this non-proliferative cluster show expression of well-known light zone markers, i.e. CD83, LMO2, and downregulation of DZ genes such as CXCR4 or AICDA. Within this cluster, genes related to CD40 signaling and response to BCR stimulation are starting to be expressed (e.g. BCL2A1, BCL2L1, TRAF1/4 or EBI3). However, the expression levels of these genes were not homogeneous, suggesting the presence of different levels of activation of LZ cells.

LZ-DZ re-entry commitment: Cluster with high expression of CD83 and low expression of CXCR4, as well as positive for LMO2, which indicate that this cluster contains LZ-GCBC cells. Within this cluster, apart from the high expression of genes associated with the previously described LZ cluster, the cells start to express very specific genes such as MYC, MIR155HG or genes related to NF-kB pathway (i.e. NFKB1, NFKB2, NFKBIA, NFKBID, RELB). This transient expression of MYC has been described in LZ cells re-entering into the DZ, to continue with additional rounds of affinity maturation (Dominguez-Sola et al., 2012). Additionally, as compared to the LZ cluster, this cluster shows higher expression of BATF, a gene known to be involved in regulating AIDCA (a DZ marker) (Ise et al., 2011). From the scATAC-seq perspective, this LZ-DZ re-entry cluster is associated with epigenetic programming, with transiently increased chromatin accessibility associated with TFs of the NF-kB family as well as BATF. Altogether, these observations suggest that this cluster contains LZ cells that are already committed and preparing to return to the DZ.

LZ-proliferative: This cluster has a proliferative signature, marked for example by upregulation of MCM gene family, PCNA, POLA1, while still maintaining the expression of standard light zone markers, like CD83 and LMO2. Furthermore, these cells maintain the expression of specific genes related to the LZ-DZ re-entry commitment cluster, such as BCL2A1, MIR155HG, NF-kB pathway genes or BATF. Therefore, this cluster may represent LZ cells that are starting to proliferate.

LZ-DZ transition: This proliferative cluster presents intermediate expression of both LZ and DZ markers, i.e. containing traces of both CD83 and CXCR4 expression. It also downregulates previously mentioned signature related to LZ-DZ re-entry commitment, but cell cycle genes are highly expressed. Therefore, these cells may represent the intermediate transitional step from proliferative LZ to proliferative DZ cells. It also suggests that the phenotypic switch from LZ to DZ occurs while cells proliferate.

Precursor MBC: Within the level 1 cluster containing GCBCs, we also identified a subset of cells that were characterized by MBC-related markers while maintaining a non-proliferative GC signature. Specifically, this cluster was characterized by high expression of CCR6 or CELF2, genes that are commonly used in literature to define memory B cell precursors (Holmes et al., 2020). Although this cluster likely represents early MBCs derived from the LZ, we cannot exclude some technical issues or even MBC-GC doublet cells.

Reactivated proliferative MBC: This is a second cluster within the GC compartment that contains MBC markers. In this case, cells show high expression of BANK1 and TNFRSF13B (CD267). However, in the contrary to precursor MBC (that supposedly are generated from non-proliferative LZ cells), this cluster is characterized by high proliferation signature (i.e. PCNA, MKI67 or TOP2A expression). Therefore, we hypothesize that these cells may be derived from secondary immune responses, in which MBC are reactivated, start to rapidly proliferate and may eventually differentiate into plasma cells. However, the overlap between some GC and MBC markers does not allow us to unequivocally exclude doublets.

PLASMA CELLS

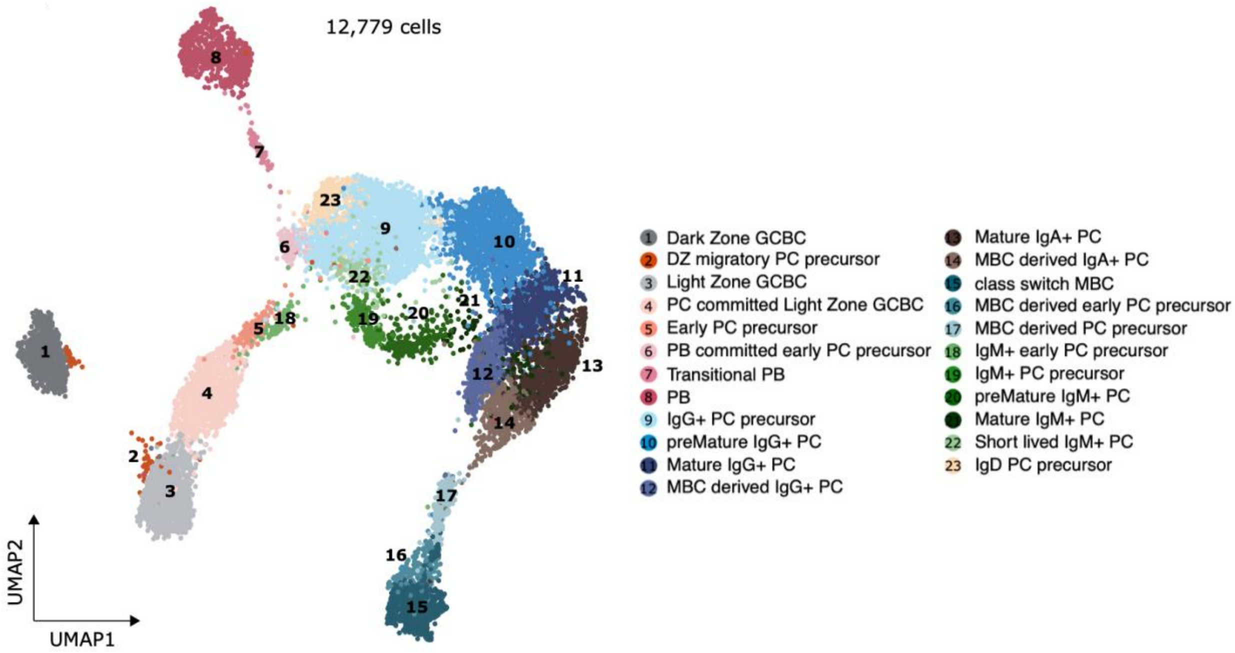

*For the sake of mapping cell transitions, in this analysis we included *Dark-Zone* and *Light-Zone GCBCs* as well as *class-switched MBCs* from the GCBC and NBC/MBC level 1 clusters, respectively. These subpopulations will not be described in this section of the glossary but in their respective clusters.

DZ migratory PC precursor: This cluster is composed by a relatively small number of cells that belong to the PC cluster at level 1, but in the PC-specific UMAP unexpectedly cluster close to both non-proliferative DZ-GCBCs and LZ-GCBCs (which were borrowed from the GCBC cluster). This intriguing cluster shows expression of GC markers such as BCL6, IRF8 and MEF2B, as well as intermediate levels of DZ (e.g.CXCR4 and AICDA) and LZ markers (e.g. CD83 and LMO2). Additionally, expression of B cell markers such as MS4A1 and PAX5 can be observed. These observations may be consistent with published evidence in mice suggesting that there are some maturing PCs migrating from the LZ to the DZ before leaving the GC (Kräutler et al., 2017). Although this hypothesis is attractive, we cannot formally rule out that these cells may represent doublets from PCs and GCBCs.

PC committed light zone GCBC: This subpopulation still belongs to the GCBCs level 1 cluster (it is described within this plasma cell section of the glossary). It maintains high levels of B cell markers such as MS4A1 and PAX5, as well as high levels of GC markers such as BCL6, IRF8 and MEF2B, and LZ markers (MME (CD10), and CD83). However, it shows the first seeds of commitment towards PC differentiation, which are associated mostly with the expression, still at low levels, of the three key PC-related transcription factors PRDM1, XBP1 and IRF4. (Nutt et al., 2015)

Early PC precursor: This cluster belongs to the PC level 1 cluster, and is located next to the PC-committed LZ-GCBCs. Although it still maintains the expression of B cell and GC markers, their levels are reduced as compared to the PC committed cells (e.g. MS4A1, BCL6, IRF8, CD83). On the contrary, the expression of the 3 PC transcription factors (PRDM1, XBP1 and IRF4) (Nutt et al., 2015) are increased as compared to PC committed cells, and the first traces of PC phenotypic markers can be detected, e.g. MZB1, SLAMF7, SSR4, CREB3L2 and FKBP11.

PB committed early PC precursor: Similar expression profile to the Early PC precursors, with low levels of B cell and GC markers (e.g. MS4A1, BCL6, IRF8, CD83), high expression expression levels of the 3 PC transcription factors (PRDM1, XBP1 and IRF4) (Nutt et al., 2015) and traces of PC phenotypic markers, e.g. MZB1, SLAMF7, SSR4, CREB3L2 and FKBP11. CD9, a marker for precursor PCs (Yoon et al., 2013), can be detected. However, in this cluster, low levels of some cell division-related genes such as H2AFZ and TUBA1B. (Bar-Joseph et al., 2008). Therefore, we interpret that these early PC precursors are already committed to become proliferative plasmablasts.

Transitional PB: Cells in this cluster express the 3 PC transcription factors (PRDM1, XBP1 and IRF4) (Nutt et al., 2015) and PC phenotypic markers such as MZB1, SLAMF7, SSR4, CREB3L2 and FKBP11. CD9, a marker for precursor PCs (Yoon et al., 2013), can be detected. This is a peculiar cluster with intermediate levels of genes characteristic for proliferative plasmablasts such as H2AFZ, TUBA1B, MKI67 and TOP2A (Bar-Joseph et al., 2008). An analysis of cell cycle phases indicates that this cluster has cells both in S phase and G2M phase, which may suggest that it contains cells transitioning from PB committed early PC precursors to proliferative PBs and vice versa.

PB: Cells within this cluster are clearly proliferative plasmablasts with high expression levels of proliferation-related genes (H2AFZ, TUBA1B, MKI67 and TOP2A, among others). Otherwise have an expression pattern of PCs (the 3 transcription factors PRDM1, IRF4 and XBP1, as well as phenotypic markers MZB1, SLAMF7, SSR4, CREB3L2 and FKBP11) (Nutt et al., 2015). CD9, a marker for precursor PCs (Yoon et al., 2013), can be detected. As compared to transitional PBs, the expression levels of PC phenotypic markers are slightly increased. Traces of B cell markers can still be detected (e.g. MS4A1 and PAX5) and even some GC-related markers such as MEF2B.

IgG+ PC precursor: Cells within this cluster have a typical PC markers (the 3 transcription factors PRDM1, IRF4 and XBP1, as well as phenotypic markers MZB1, SLAMF7, SSR4, CREB3L2 and FKBP11) (Nutt et al., 2015). CD9, a marker for precursor PCs (Yoon et al., 2013), can be detected.. Although the typical B cell-related genes (MS4A1 and PAX5, among others) are downregulated, some traces of MEF2B, a GC marker, can still be observed. Finally, as compared to early precursors, higher expression levels of IGHG can be detected in this cluster.

PreMature IgG+ PC: Cells within this cluster have a typical PC markers (the 3 transcription factors PRDM1, IRF4 and XBP1, as well as phenotypic markers MZB1, SLAMF7, SSR4, CREB3L2 and FKBP11). (Nutt et al., 2015). CD9, a marker for precursor PCs (Yoon et al., 2013) can still be detected. Although the typical B cell-related genes (MS4A1 and PAX5, among others) are downregulated, some traces of MEF2B, a GC marker, can still be observed. Finally, as compared to IgG+ PC precursors, higher expression levels of IGHG can be detected in this cluster.

Mature IgG+ PC: Cells within this cluster have a typical PC markers (the 3 transcription factors PRDM1, IRF4 and XBP1, as well as phenotypic markers MZB1, SLAMF7, SSR4, CREB3L2 and FKBP11) (Nutt et al., 2015). CD9, a marker for precursor PCs (Yoon et al., 2013) is downregulated, and CD44, a marker for mature PCs (Khodadadi et al., 2019), is expressed. Traces of MEF2B, a GC marker, or typical B-cell related genes (e.g. MS4A1) cannot be detected anymore. Finally, this subpopulation shows high expression levels of IGHG.

MBC-derived IgG+ PC: Cells within this cluster have a typical PC markers (the 3 transcription factors PRDM1, IRF4 and XBP1, as well as phenotypic markers MZB1, SLAMF7, SSR4, CREB3L2 and FKBP11) (Nutt et al., 2015). CD9, a marker for precursor PCs (Yoon et al., 2013) is downregulated, and CD44, a marker for mature PCs (Khodadadi et al., 2019), is expressed. This subpopulation shows high expression levels of IGHG. Remarkably, cells in this cluster show expression of some markers expressed in MBCs, and in MBC-derived precursor PCs, such as FCMR and CCDC50. Therefore, the mature IgG+ PC phenotype together with traces of MBC-related genes made us think that this cluster represents mature IgG+ PCs derived from the reactivation of MBCs. Nevertheless, we cannot discard that these cells are doublets from MBC and PC.

Mature IgA+ PC: Cells within this cluster have a typical PC markers (the 3 transcription factors PRDM1, IRF4 and XBP1, as well as phenotypic markers MZB1, SLAMF7, SSR4, CREB3L2 and FKBP11) (Nutt et al., 2015). CD9, a marker for precursor PCs (Yoon et al., 2013) is downregulated, and CD44, a marker for mature PCs (Khodadadi et al., 2019), is expressed. Traces of MEF2B, a GC marker, or typical B-cell related genes (e.g. MS4A1) cannot be detected anymore. Finally, this subpopulation shows high expression levels of IGHA.

MBC-derived IgA+ PC: Cells within this cluster have a typical PC markers (the 3 transcription factors PRDM1, IRF4 and XBP1, as well as phenotypic markers MZB1, SLAMF7, SSR4, CREB3L2 and FKBP11) (Nutt et al., 2015). CD9, a marker for precursor PCs is downregulated (Yoon et al., 2013), and CD44, a marker for mature PCs, is expressed (Khodadadi et al., 2019). This subpopulation shows high expression levels of IGHA. Remarkably, cells in this cluster show expression of some markers expressed in MBCs, and in MBC-derived precursor PCs, such as FCMR and CCDC50. Therefore, the mature IgA+ PC phenotype together with traces of MBC-related genes made us think that this cluster represents mature IgA+ PCs derived from the reactivation of MBCs. Nevertheless, we cannot discard that these cells are doublets from MBC and PC.

MBC-derived early PC precursor: This subpopulation clusters next to the csMBCs that we borrowed from the MBC cluster at level 1. As compared to csMBCs, these cells show low levels of PC related genes (PRDM1, IRF4, XBP1, MZB1, SLAMF7, SSR4, CREB3L2 and FKBP11) (Nutt et al., 2015), and increased levels of IG genes expression (although still low). Traces of B cell related genes are expressed such as MS4A1 and PAX5. Additionally, MBC-related genes are also expressed (i.e. FCMR and CCDC50). Therefore, we interpret that this subpopulation represents the early PC precursors derived from the reactivation of MBCs.

MBC-derived PC precursor: This subpopulation clusters between MBC-derived early PC precursor and mature PCs. As compared to MBC-derived early PC precursor, these cells show higher levels of PC related genes (PRDM1, IRF4, XBP1, MZB1, SLAMF7, SSR4, CREB3L2 and FKBP11) (Nutt et al., 2015), and increased levels of IG genes expression. Traces of B cell related genes are expressed such as MS4A1 and PAX5. Additionally, MBC-related genes are also expressed (i.e. FCMR and CCDC50). Therefore, due to the increased expression of PC and IG genes as compared to the MBC-derived early PC precursor, we interpret that this cluster represents a further step towards the phenotypic transformation of a MBC into a PC. Nevertheless, we cannot discard that these cells are doublets from MBC and PC.

IgM+ early PC precursor: This cluster maps close to the early PC precursor cells but consistently show low expression levels of IGHM. The global gene expression pattern is similar to early PC precursor cells. It maintains low expression levels of B cell and GC markers, (e.g. MS4A1, BCL6, IRF8, CD83), and shows expression of the 3 PC transcription factors (PRDM1, XBP1 and IRF4) and the first traces of PC phenotypic markers can be detected, e.g. MZB1, SLAMF7, SSR4, CREB3L2 and FKBP11(Nutt et al., 2015).

IgM+ PC precursor: Cells within this cluster express IGHM and have a typical PC markers (the 3 transcription factors PRDM1, IRF4 and XBP1, as well as phenotypic markers MZB1, SLAMF7, SSR4, CREB3L2 and FKBP11) (Nutt et al., 2015). CD9, a marker for precursor PCs (Yoon et al., 2013), can be detected. Although the typical B cell-related genes (MS4A1 and PAX5, among others) are downregulated, some traces of MEF2B, a GC marker, can still be observed. Finally, as compared to IgM+ early PC precursor, higher expression levels of IGHM can be detected.

Premature IgM+ PC: This IGHM-positive cluster show the expression of typical PC markers (the 3 transcription factors PRDM1, IRF4 and XBP1, as well as phenotypic markers MZB1, SLAMF7, SSR4, CREB3L2 and FKBP11) (Nutt et al., 2015). CD9, a marker for precursor PCs (Yoon et al., 2013) is downregulated, and the expression of CD44, a marker for more mature PCs (Khodadadi et al., 2019), is increased. Finally, as compared to IgM+ PC precursor, higher expression levels of IGHM can be detected.

Mature IgM+ PC: Cells within this cluster express IGHM and have a typical PC markers (the 3 transcription factors PRDM1, IRF4 and XBP1, as well as phenotypic markers MZB1, SLAMF7, SSR4, CREB3L2 and

FKBP11) (Nutt et al., 2015). CD9, a marker for precursor PCs (Yoon et al., 2013) is downregulated. Traces typical B-cell related genes (e.g. MS4A1) cannot be detected. Finally, among the IgM+ clusters, this subpopulation shows the highest expression levels of IGHM.

Short lived IgM+ PCs: Cells within this cluster express IGHM and have a typical PC markers (the 3 transcription factors PRDM1, IRF4 and XBP1, as well as phenotypic markers MZB1, SLAMF7, SSR4, CREB3L2 and FKBP11) (Nutt et al., 2015). CD9, a marker for precursor PCs (Yoon et al., 2013), can also be detected. Although relatively similar to IgM+ PC precursor, this cluster shows a global gene downregulation with the exception of few upregulated genes strongly enriched in the endoplasmic reticulum cellular component concomitantly to high IgM expression. Therefore, we conclude that this may represent a subpopulation of short lived IgM PC cells that shut down most of the cellular functions with the exception of high production of IgM which is processed through the endoplasmic reticulum.

IgD+ PC precursors: Cells within this cluster express IGHD and have a typical PC markers (the 3 transcription factors PRDM1, IRF4 and XBP1, as well as phenotypic markers MZB1, SLAMF7, SSR4, CREB3L2 and FKBP11)(Nutt et al., 2015). CD9, a marker for precursor PCs (Yoon et al., 2013), can be detected. Although the typical B cell-related genes (MS4A1 and PAX5, among others) are downregulated, some traces of MEF2B, a GC marker, can still be observed.

MYELOID CELLS

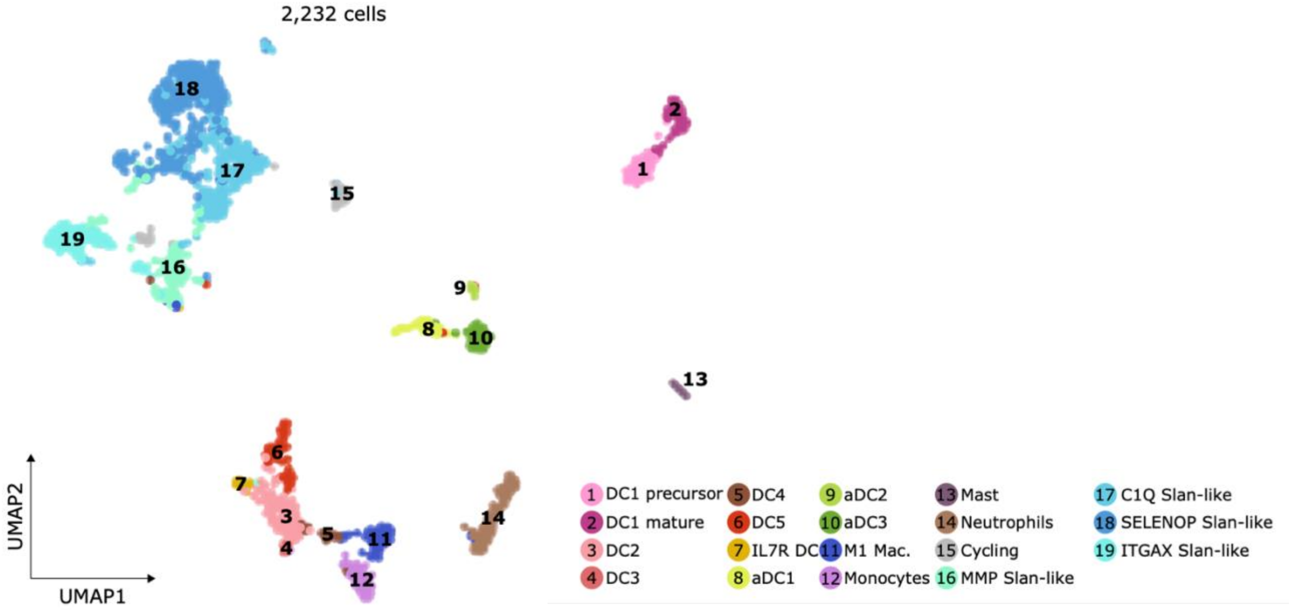

DC1 precursor: DC1 corresponds to the cross-presenting conventional DC1 (cDC1, CD141+ DC, BDCA-3), which was reclassified by (Villani et al., 2017) as DC1 using scRNA-seq. This cluster is best marked by *CLEC9A,* and the top gene markers are*: CLEC9A, IDO1, CADM1, CST3, SNX3, C1orf54, HLA-DPB1, IRF8, CCND1* and *HLA-DPA1.* The low levels of XCR1 together with an increased proliferation signature with respect to DC1 mature cells identify this population as a DC1 precursor (Balan et al., 2018; Villani et al., 2017).

DC1 mature: This cluster had a similar profile to DC1 precursor, expressing higher levels of *CLEC9A* and *XCR1*, and acquiring a mature phenotype. DC1 mature cells had fewer cells in S or G2M phase and a decreased proliferation signature as compared to DC1 precursors (Balan et al., 2018; Villani et al., 2017).

DC2: Conventional DC2 (cDC2, CD1C+ DC, BDCA-1) were reclassified by (Villani et al., 2017) as DC2 (expressing MHC class II genes) and DC3 (monocyte-like and inflammatory genes) using scRNA-seq. CD1C was the main marker and unique that identified both DC2 and DC3 clusters. This subpopulation expressed high levels of *CD1C*, *CLEC10A, FCER1A, HLA-DQB1, HLA-DRB1, HLA-DQA1 and HLA-DPB1.* This higher expression of MHC class II genes differentiates this population from DC3 (Villani et al., 2017).

DC3: Conventional DC2 (cDC2, CD1C+ DC, BDCA-1) were reclassified by (Villani et al., 2017) as DC2 (expressing MHC class II genes) and DC3 (monocyte-like and inflammatory genes) using scRNA-seq. The higher expression of CD1C identified these populations as the second cluster of CD1C DC cells. This cluster expressed CD1C, *CLEC10A*, *FCER1A*, *S100A8, S100A9, VCAN, LYZ* and *ANXA1*. This inflammatory signature classified this subpopulation as a different one compared with DC2 that had more antigen presentation features (Villani et al., 2017).

DC4: DC4 corresponds to CD1C–CD141–CD11C+ DC, which is best marked by CD16 (encoded by *FCGR3A*) and shares signatures with monocytes. DC4 might differentiate from non-classical monocytes (CD16+). This cluster expressed *FCGR3A, FTL, SIGLEC10, SERPINA1, LST1* and *AIF1* and it was CD1C- (Villani et al., 2017).

DC5: This cluster represents “AS DCs” (AXL+ SIGLEC6+ DCs). DC5 share marker genes with cDC and with plasmacytoid DC. This subpopulation had as top marker genes *SIGLEC6, AXL, CDH1, CD22* and *LILRA4* (Villani et al., 2017).

IL7R DC: This cluster was identified for its higher expression of *IL7R* and the expression of: *NR4A3, CLDN1, NRARP, CLEC10A, AXL*, and *IL4I1*. This cluster might represent pDC or DC5 precursors, but additional studies are needed to characterize this better (Rodrigues et al., 2018).

aDC1, aDC2 and aDC3: These three subtypes of activated DC cells were recently described by Park et al. These three subtypes were characterized by the expression of *LAMP3* and *CCR7*. aDC1 has as top genes: *SLCO5A1, CCR7, DUSP5, LAD1, TREML1* and *LAMP3*. aDC2 expresses *PRDM16, CCNA1, SLC16A2, S100A2, AIRE* and *CCL22.* aDC3 expressed *CCL19, MARCKSL1, FSCN1, ACTG1, SYNPO* and *TMSB10* (Park et al., 2020). Contrary to the thymus atlas, we could not assign aDC subsets to their DC counterpart. aDC2 was composed of very few cells, and thus we could not validate it in the validation cohort. Thus, future studies with more power are needed to confirm the presence of this cluster.

M1 Macrophages: This population had a higher expression of *CD68* and the top gene markers are an inflammatory signature of M1 profile. This cluster has top gene markers: *CCL4, IL1B, CCL3, TNF, IL6, IL1A* and *PTX3* (Barros et al., 2013).

Monocytes: This cluster was identified by its expression of *CD14* and the expression of canonical genes well described for monocytes. Top markers of this cluster: *VCAN, S100A9, S100A8, FCN1, S100A4* and *S100A12* (Villani et al., 2017).

Mast: This cluster has expressions of classical markers for Mast cells *KIT*, *TPSAB1* and *TPSB2*. Top markers of this cluster were: *TPSAB1, TPSB2, CPA3, MS4A2, LTC4S, HPGD, HPGDS* and *LMO4* (Wu et al., 2022a).

Neutrophils: This cluster expressed specific markers previously described for neutrophils. Top gene markers: *CXCL8, G0S2, PI3, CCL3L1, FCGR3B, PTGS2,* and *IFITM2* (Travaglini et al., 2020; Williams et al., 2021).

Cycling: This cluster is characterized by a high proliferation signature expressing *CCNB2, PCNA, MKi67* and *TOP2A* (Bar-Joseph et al., 2008).

MMP Slan-like: This cluster had a profile of tonsil slan+ cells expressing higher levels of *MMP9, MMP12, MMP14, HLA-DRA, HLA-DRB, CCR1, FCGR2A, ADAMDEC1* and *C1QC,* and intermediate levels of *NECTIN2* and *FUCA1* (Bianchetto-Aguilera et al., 2020). Based on GO terms, these MMP slancytes could play a role in antigen presentation, activation and matrix remodeling (Hofer et al., 2015).

C1Q Slan-like: This cluster had higher levels of tonsil slan+ cells such as *C1QC, APOE,* and *MMP9,* and intermediate levels of *FUCA1, APOC1* and *ADAMDEC1*. This cluster had a profile to respond to classical complement activation with higher expression of C1Q family genes: *C1QC, C1QB* and *C1QA*. Also, This cluster had a higher expression of MHC class II genes *HLA-DQA1, HLA-DRA, HLA-DRB1, HLA-DPA1* and *HLA-DPB1*, demonstrating their potential role in antigen presentation (Bianchetto-Aguilera et al., 2020) .

SELENOP Slan-like: These SELENOP slan-like expressed a tonsil slan+ cell signature with high levels of *SELENOP*, *FOLR2*, *PTGDS*, *FUCA1, APOE*, and *APOC1*. This cluster expressed higher levels of *CCL18* suggesting it can be involved in the recruitment of MMP slan-like via CCL18-CCR1 axis. This cluster had an OXPHOS related signature pointing out their possible metabolic role in tonsils (Bianchetto-Aguilera et al., 2020).

ITGAX Slan-like: This cluster expressed tonsil slan+ cell markers: *FUCA1, NR1H3*, and *APOE.* This cluster has an inflammatory signature expressing *ITGAX, ZEB2, IL6R* and *IL18.* It also expressed *MAF*, *MAFB*, and *MAFG* (Bianchetto-Aguilera et al., 2020).

EPITHELIAL CELLS

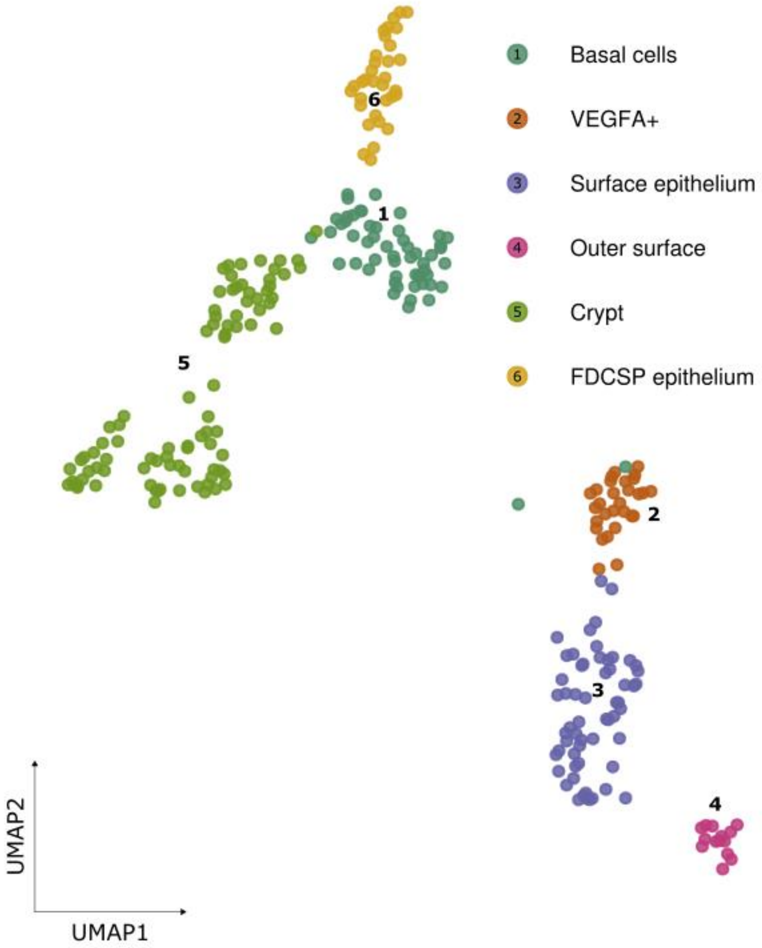

Basal cells: This cluster represents the basal layer of the squamous epithelium, characterized by the expression of the basal cytokeratins *KRT5* and *KRT14* (Williams et al., 2021).

Surface keratinocytes: Cells in this cluster represent the main bulk of the surface epithelium with expression of several cytokeratins (*KRT4, KRT13, KRT78, KRT80*), *SPRR3, TMPRSS11B* and *TMPRSS2* (Williams et al., 2021) .

VEGFA+: Cells in this cluster express *VEGFA*, which has been shown to be expressed by subsets of epithelial cells in other organs (Ellis et al., 2020).

Outer surface: These cells express several genes associated with cornification/keratinization like *CNFN* and *SPRR2* genes and represent the outermost layer of the surface epithelium (Williams et al., 2021).

FDCSP epithelium: This cluster is characterized by the high expression of KRTDAP, CALML5, and FDCSP. FDCSP was discovered in 2002 as a marker highly specific to follicular dendritic cells (Marshall et al., 2002). In the same article, the authors reported a FDCSP-expressing population of leukocyte-infiltrated tonsil crypts. This cluster might reflect this cell type. Of note, the oral mucosa cell atlas also reported a population of FDCSP-expressing epithelial cells (Williams et al., 2021).

Crypt: Cells in this cluster express *KRT8* and genes involved in inflammatory responses (*IL1B*, *IFI27*) and represent the epithelial crypts of the tonsils. Also in these clusters are cells which express *SPIB* and *MARCKSL1*, two genes associated with microfold cells (M cells) (Kobayashi et al., 2019)

MESENCHYMAL CELLS/FOLLICULAR DENDRITIC CELLS

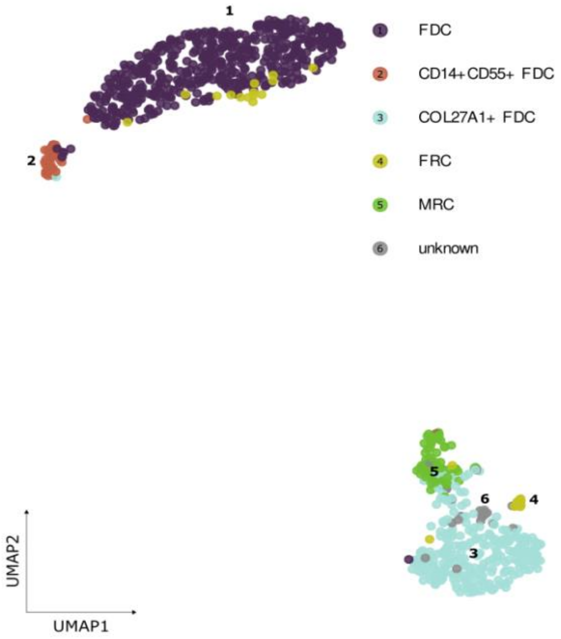

FDC: This is the main cluster of follicular dendritic cells, with high expression of the classical FDC markers *FDCSP*, *CLU* and *CXCL13* (Heesters et al., 2021).

CD14+CD55+ FDC: This cluster, besides high expression levels of *FDCSP*, *CLU*, *CR2*, and *CXCL13*, additionally expresses *CD14* and *CD55*. CD14 expression in FDC is described in the literature as an independent predictor of transformation in follicular lymphoma. (Smeltzer et al., 2014).

COL27A1+ FDC: Cells in this cluster show lower expression levels of *FDCSP*, *CLU*, *CR2*, and *CXCL13* compared to the main FDC cluster and high expression of *COL27A1* (Rodda et al., 2018).

FRC: This cluster expresses *CCL20*, *PERP*, *S100A2* and *S100A9* markers associated with fibroblastic reticular cells (Rodda et al., 2018; Heesters et al., 2021).

MRC: Cells in this cluster express several collagen genes (*COL1A1*, *COL1A2*, *COL3A1*, *COL5A2*, *COL6A3*, *COL12A1*), as well as *DCN* and *PDGFRB*. This cluster was annotated as marginal reticular cells according to recently published literature. Based on *PDGFRB* expression at least part of these cells might represent perivascular precursors, as described in the literature in murine models (Jiang et al., 2021; Heesters et al., 2021).

Unknown: Cells in this cluster had as markers several genes from the KLF family (KLF2/4/6) as well as other markers such as EGR1, DUSP1, ZFP36, NFKBIZ, SELE, VMP1, ADAMTSL4-AS1, AC020916.1, AC007952.4. However, this cluster also showed as markers distinct stress signature responses such as FOS, JUN or NEAT1 and hence we did not know whether to exclude this cluster as it may be composed of poor-quality cells. Additional studies are needed to make sense of the identity of this cluster.

Cycling FDC: This a cluster of follicular dendritic cells, with high expression of the classical FDC markers and a specific gene signature of cycling. The top expressed genes are: *HIST1H4C, HMGB2, STMN1, H2AFZ, HMGB1, HMGN2, NUSAP1, TUBA1B* and *PTTG1*. As we observed that the proliferative signature was a major driver of variance in scRNA-seq data, which caused (1) cells from different lineages to cluster together and (2) hindered biological heterogeneity in the other cluster, we show this population in Figure 1B, but exclude it from downstream analysis. (Bar-Joseph et al., 2008)

GLOSSARY REFERENCES

Agarwal, S., Kraus, Z., Dement-Brown, J., Alabi, O., Starost, K., Tolnay, M., 2020. Human Fc Receptor-like 3 Inhibits Regulatory T Cell Function and Binds Secretory IgA. Cell Reports 30, 1292-1299.e3. https://doi.org/10.1016/j.celrep.2019.12.099

Akkaya, M., Kwak, K., Pierce, S.K., 2020. B cell memory: building two walls of protection against pathogens. Nat Rev Immunol 20, 229–238. https://doi.org/10.1038/s41577-019-0244-2

Bal, S.M., Golebski, K., and Spits, H. (2020). Plasticity of innate lymphoid cell subsets. Nat. Rev. Immunol. *20*, 552–565. https://doi.org/10.1038/s41577-020-0282-9.

Balan, S., Arnold-Schrauf, C., Abbas, A., Couespel, N., Savoret, J., Imperatore, F., Villani, A.-C., Vu Manh, T.-P., Bhardwaj, N., and Dalod, M. (2018). Large-Scale Human Dendritic Cell Differentiation Revealing Notch-Dependent Lineage Bifurcation and Heterogeneity. Cell Rep. *24*, 1902-1915.e6. https://doi.org/10.1016/j.celrep.2018.07.033.

Bar-Joseph, Z., Siegfried, Z., Brandeis, M., Brors, B., Lu, Y., Eils, R., Dynlacht, B.D., Simon, I., 2008. Genome-wide transcriptional analysis of the human cell cycle identifies genes differentially regulated in normal and cancer cells. Proc Natl Acad Sci U S A 105, 955–960. https://doi.org/10.1073/pnas.0704723105

Barros, M.H.M., Hauck, F., Dreyer, J.H., Kempkes, B., Niedobitek, G., 2013. Macrophage Polarisation: an Immunohistochemical Approach for Identifying M1 and M2 Macrophages. PLOS ONE 8, e80908. https://doi.org/10.1371/journal.pone.0080908

Bianchetto-Aguilera, F., Tamassia, N., Gasperini, S., Calzetti, F., Finotti, G., Gardiman, E., Montioli, R., Bresciani, D., Vermi, W., and Cassatella, M.A. (2020). Deciphering the fate of slan $^{+}$ -monocytes in human tonsils by gene expression profiling. FASEB J. *34*, 9269–9284. https://doi.org/10.1096/fj.202000181R.

Boldt, A., Borte, S., Fricke, S., Kentouche, K., Emmrich, F., Borte, M., Kahlenberg, F., and Sack, U. (2014). Eight-color immunophenotyping of T-, B-, and NK-cell subpopulations for characterization of chronic immunodeficiencies. Cytometry B Clin. Cytom. *86*, 191–206. https://doi.org/10.1002/cyto.b.21162.

Booth, C., Gilmour, K.C., Veys, P., Gennery, A.R., Slatter, M.A., Chapel, H., Heath, P.T., Steward, C.G., Smith, O., O'Meara, A., Kerrigan, H., Mahlaoui, N., Cavazzana-Calvo, M., Fischer, A., Moshous, D., Blanche, S., Pachlopnik Schmid, J., Latour, S., de Saint-Basile, G., Albert, M., Notheis, G., Rieber, N., Strahm, B., Ritterbusch, H., Lankester, A., Hartwig, N.G., Meyts, I., Plebani, A., Soresina, A., Finocchi, A., Pignata, C., Cirillo, E., Bonanomi, S., Peters, C., Kalwak, K., Pasic, S., Sedlacek, P., Jazbec, J., Kanegane, H., Nichols, K.E., Hanson, I.C., Kapoor, N., Haddad, E., Cowan, M., Choo, S., Smart, J., Arkwright, P.D., Gaspar, H.B., 2011. X-linked lymphoproliferative disease due to SAP/SH2D1A deficiency: a multicenter study on the manifestations, management and outcome of the disease. Blood 117, 53–62. https://doi.org/10.1182/blood-2010-06-284935

Brewitz, A., Eickhoff, S., Dähling, S., Quast, T., Bedoui, S., Kroczek, R.A., Kurts, C., Garbi, N., Barchet, W., Iannacone, M., et al. (2017). CD8+ T Cells Orchestrate pDC-XCR1 + Dendritic Cell Spatial and Functional Cooperativity to Optimize Priming. Immunity *46*, 205–219. https://doi.org/10.1016/j.immuni.2017.01.003.

Burger, J.A., Chiorazzi, N., 2013. B cell receptor signaling in chronic lymphocytic leukemia. Trends Immunol 34, 592–601. https://doi.org/10.1016/j.it.2013.07.002

Cano-Gamez, E., Soskic, B., Roumeliotis, T.I., So, E., Smyth, D.J., Baldrighi, M., Willé, D., Nakic, N., Esparza-Gordillo, J., Larminie, C.G.C., et al. (2020). Single-cell transcriptomics identifies an effectorness gradient shaping the response of CD4+ T cells to cytokines. Nat. Commun. *11*, 1801. https://doi.org/10.1038/s41467-020-15543-y.

Cao, W., and Bover, L. (2010). Signaling and ligand interaction of ILT7: receptor-mediated regulatory mechanisms for plasmacytoid dendritic cells. Immunol. Rev. *234*, 163–176. https://doi.org/10.1111/j.0105-2896.2009.00867.x.

Choi, J., and Crotty, S. (2021). Bcl6-Mediated Transcriptional Regulation of Follicular Helper T cells (TFH). Trends Immunol. *42*, 336–349. https://doi.org/10.1016/j.it.2021.02.002.

Cibrián, D., and Sánchez-Madrid, F. (2017). CD69: from activation marker to metabolic gatekeeper. Eur. J. Immunol. *47*, 946–953. https://doi.org/10.1002/eji.201646837.

Crotty, S. (2011). Follicular Helper CD4 T Cells (T $_{FH}$). Annu. Rev. Immunol. *29*, 621–663. https://doi.org/10.1146/annurev-immunol-031210-101400.

Crotty, S. (2014). T Follicular Helper Cell Differentiation, Function, and Roles in Disease. Immunity *41*, 529–542. https://doi.org/10.1016/j.immuni.2014.10.004.

Crotty, S. (2019). T Follicular Helper Cell Biology: A Decade of Discovery and Diseases. Immunity *50*, 1132–1148. https://doi.org/10.1016/j.immuni.2019.04.011.

Cuenca, M., Romero, X., Sintes, J., Terhorst, C., and Engel, P. (2016). Targeting of Ly9 (CD229) Disrupts Marginal Zone and B1 B Cell Homeostasis and Antibody Responses. J. Immunol. Baltim. Md 1950 *196*, 726–737. https://doi.org/10.4049/jimmunol.1501266.

Damsker, J.M., Hansen, A.M., and Caspi, R.R. (2010). Th1 and Th17 cells: Adversaries and collaborators. Ann. N. Y. Acad. Sci. *1183*, 211–221. https://doi.org/10.1111/j.1749-6632.2009.05133.x.

Dominguez-Sola, D., Victora, G.D., Ying, C.Y., Phan, R.T., Saito, M., Nussenzweig, M.C., and Dalla-Favera, R. (2012). The proto-oncogene MYC is required for selection in the germinal center and cyclic reentry. Nat. Immunol. *13*, 1083–1091. https://doi.org/10.1038/ni.2428.

Du, Y., Fang, Q., and Zheng, S.-G. (2021). Regulatory T Cells: Concept, Classification, Phenotype, and Biological Characteristics. In T Regulatory Cells in Human Health and Diseases, S.-G. Zheng, ed. (Singapore: Springer Singapore), pp. 1–31.

Ellis, L.V., Cain, M.P., Hutchison, V., Flodby, P., Crandall, E.D., Borok, Z., Zhou, B., Ostrin, E.J., Wythe, J.D., Chen, J., 2020. Epithelial Vegfa Specifies a Distinct Endothelial Population in the Mouse Lung. Developmental Cell 52, 617-630.e6. https://doi.org/10.1016/j.devcel.2020.01.009

Ferrer, G., Bosch, R., Hodgson, K., Tejero, R., Roué, G., Colomer, D., Montserrat, E., and Moreno, C. (2014). B cell activation through CD40 and IL4R ligation modulates the response of chronic lymphocytic leukaemia cells to BAFF and APRIL. Br. J. Haematol. *164*, 570–578. https://doi.org/10.1111/bjh.12645.

Fu, N., Xie, F., Sun, Z., Wang, Q., 2021. The OX40/OX40L Axis Regulates T Follicular Helper Cell Differentiation: Implications for Autoimmune Diseases. Frontiers in Immunology 12.

Galletti, G., De Simone, G., Mazza, E.M.C., Puccio, S., Mezzanotte, C., Bi, T.M., Davydov, A.N., Metsger, M., Scamardella, E., Alvisi, G., et al. (2020). Two subsets of stem-like CD8+ memory T cell progenitors with distinct fate commitments in humans. Nat. Immunol. *21*, 1552–1562. https://doi.org/10.1038/s41590-020-0791-5.

Galván-Peña, S., Leon, J., Chowdhary, K., Michelson, D.A., Vijaykumar, B., Yang, L., Magnuson, A.M., Chen, F., Manickas-Hill, Z., Piechocka-Trocha, A., et al. (2021). Profound Treg perturbations correlate with COVID-19 severity. Proc. Natl. Acad. Sci. *118*, e2111315118. https://doi.org/10.1073/pnas.2111315118.

Gattinoni, L., Lugli, E., Ji, Y., Pos, Z., Paulos, C.M., Quigley, M.F., Almeida, J.R., Gostick, E., Yu, Z., Carpenito, C., Wang, E., Douek, D.C., Price, D.A., June, C.H., Marincola, F.M., Roederer, M., Restifo, N.P., 2011. A human memory T cell subset with stem cell–like properties. Nat Med 17, 1290–1297. https://doi.org/10.1038/nm.2446

Gerlach, C., Moseman, E.A., Loughhead, S.M., Alvarez, D., Zwijnenburg, A.J., Waanders, L., Garg, R., Torre, J.C. de la, Andrian, U.H. von. (2016). The Chemokine Receptor CX3CR1 Defines Three Antigen-Experienced CD8 T Cell Subsets with Distinct Roles in Immune Surveillance and Homeostasis. Immunity 45, 1270–1284. https://doi.org/10.1016/j.immuni.2016.10.018

Gururajan, M., Simmons, A., Dasu, T., Spear, B.T., Calulot, C., Robertson, D.A., Wiest, D.L., Monroe, J.G., Bondada, S., 2008. Early growth response genes regulate B cell development, proliferation and immune response. J Immunol 181, 4590–4602

Heesters, B.A., van Megesen, K., Tomris, I., de Vries, R.P., Magri, G., and Spits, H. (2021). Characterization of human FDCs reveals regulation of T cells and antigen presentation to B cells. J. Exp. Med. *218*, e20210790. https://doi.org/10.1084/jem.20210790.

Hofer, T.P., Zawada, A.M., Frankenberger, M., Skokann, K., Satzl, A.A., Gesierich, W., Schuberth, M., Levin, J., Danek, A., Rotter, B., et al. (2015). slan-defined subsets of CD16-positive monocytes: impact of granulomatous inflammation and M-CSF receptor mutation. Blood *126*, 2601–2610. https://doi.org/10.1182/blood-2015-06-651331.

Holmes, A.B., Corinaldesi, C., Shen, Q., Kumar, R., Compagno, N., Wang, Z., Nitzan, M., Grunstein, E., Pasqualucci, L., Dalla-Favera, R., et al. (2020). Single-cell analysis of germinal-center B cells informs on lymphoma cell of origin and outcome. J. Exp. Med. *217*. https://doi.org/10.1084/JEM.20200483.

Ise, W., Kohyama, M., Schraml, B.U., Zhang, T., Schwer, B., Basu, U., Alt, F.W., Tang, J., Oltz, E.M., Murphy, T.L., et al. (2011). The transcription factor BATF controls the global regulators of class-switch recombination in both B cells and T cells. Nat. Immunol. *12*, 536–543. https://doi.org/10.1038/ni.2037.

Jacob, J., and Kelsoe, G. (1992). In situ studies of the primary immune response to (4-hydroxy-3-nitrophenyl)acetyl. II. A common clonal origin for periarteriolar lymphoid sheath-associated foci and germinal centers. J. Exp. Med. *176*, 679–687. https://doi.org/10.1084/jem.176.3.679.

Jia, L., and Wu, C. (2014). The Biology and Functions of Th22 Cells. In T Helper Cell Differentiation and Their Function, B. Sun, ed. (Dordrecht: Springer Netherlands), pp. 209–230.

Jiang, L., Yilmaz, M., Uehara, M., Cavazzoni, C.B., Kasinath, V., Zhao, J., Naini, S.M., Li, X., Banouni, N., Fiorina, P., et al. (2021). Characterization of Leptin Receptor+ Stromal Cells in Lymph Node. Front. Immunol. *12*, 730438. https://doi.org/10.3389/fimmu.2021.730438.

Khodadadi, L., Cheng, Q., Radbruch, A., and Hiepe, F. (2019). The Maintenance of Memory Plasma Cells. Front. Immunol. *10*, 721. https://doi.org/10.3389/fimmu.2019.00721.

King, H.W., Orban, N., Riches, J.C., Clear, A.J., Warnes, G., Teichmann, S.A., and James, L.K. (2021). Single-cell analysis of human B cell maturation predicts how antibody class switching shapes selection dynamics. Sci. Immunol. *6*. https://doi.org/10.1126/sciimmunol.abe6291.

Kobayashi, N., Takahashi, D., Takano, S., Kimura, S., Hase, K., 2019. The Roles of Peyer's Patches and Microfold Cells in the Gut Immune System: Relevance to Autoimmune Diseases. Frontiers in Immunology 10.

Kok, L., Masopust, D., and Schumacher, T.N. (2022). The precursors of CD8+ tissue resident memory T cells: from lymphoid organs to infected tissues. Nat. Rev. Immunol. *22*, 283–293. https://doi.org/10.1038/s41577-021-00590-3.

Kräutler, N.J., Suan, D., Butt, D., Bourne, K., Hermes, J.R., Chan, T.D., Sundling, C., Kaplan, W., Schofield, P., Jackson, J., et al. (2017). Differentiation of germinal center B cells into plasma cells is initiated by high-affinity antigen and completed by Tfh cells. J. Exp. Med. *214*, 1259–1267. https://doi.org/10.1084/jem.20161533.

Krueger, P.D., Goldberg, M.F., Hong, S.-W., Osum, K.C., Langlois, R.A., Kotov, D.I., Dileepan, T., and Jenkins, M.K. (2021). Two sequential activation modules control the differentiation of protective T helper-1 (Th1) cells. Immunity *54*, 687-701.e4. https://doi.org/10.1016/j.immuni.2021.03.006.

Li, H., Dement-Brown, J., Liao, P.-J., Mazo, I., Mills, F., Kraus, Z., Fitzsimmons, S., and Tolnay, M. (2020). Fc receptor-like 4 and 5 define human atypical memory B cells. Int. Immunol. *32*, 755–770. https://doi.org/10.1093/intimm/dxaa053.

Li, S., Li, K., Tian, F., Li, H., Xia, Q., Li, T., Dong, B., Li, D., Yu, J., Zhang, J., Wang, L., Zhang, C., Xu, S., Zhao, Y., Liu, Y., 2023. A high interferon gamma signature of CD8+ T cells predicts response to neoadjuvant immunotherapy plus chemotherapy in gastric cancer. Frontiers in Immunology 13.

Ma, C.S., Wong, N., Rao, G., Nguyen, A., Avery, D.T., Payne, K., Torpy, J., O'Young, P., Deenick, E., Bustamante, J., et al. (2016). Unique and shared signaling pathways cooperate to regulate the differentiation of human CD4+ T cells into distinct effector subsets. J. Exp. Med. *213*, 1589–1608. https://doi.org/10.1084/jem.20151467.

Mahnke, Y.D., Brodie, T.M., Sallusto, F., Roederer, M., and Lugli, E. (2013). The who's who of T-cell differentiation: Human memory T-cell subsets: HIGHLIGHTS. Eur. J. Immunol. *43*, 2797–2809. https://doi.org/10.1002/eji.201343751.

Mathewson, N.D., Ashenberg, O., Tirosh, I., Gritsch, S., Perez, E.M., Marx, S., Jerby-Arnon, L., Chanoch-Myers, R., Hara, T., Richman, A.R., et al. (2021). Inhibitory CD161 receptor identified in glioma-infiltrating T cells by single-cell analysis. Cell *184*, 1281-1298.e26. https://doi.org/10.1016/j.cell.2021.01.022.

McClory, S., Hughes, T., Freud, A.G., Briercheck, E.L., Martin, C., Trimboli, A.J., Yu, J., Zhang, X., Leone, G., Nuovo, G., Caligiuri, M.A., 2012. Evidence for a stepwise program of extrathymic T cell development within the human tonsil. J Clin Invest 122, 1403–1415. https://doi.org/10.1172/JCI46125

Milpied, P., Cervera-Marzal, I., Mollichella, M.-L., Tesson, B., Brisou, G., Traverse-Glehen, A., Salles, G., Spinelli, L., Nadel, B., 2018. Human germinal center transcriptional programs are de-synchronized in B cell lymphoma. Nat Immunol 19, 1013–1024. https://doi.org/10.1038/s41590-018-0181-4

Nicolet, B.P., Guislain, A., and Wolkers, M.C. (2021). CD29 Enriches for Cytotoxic Human CD4 $^{+}$ T Cells. J. Immunol. *207*, 2966–2975. https://doi.org/10.4049/jimmunol.2100138.

Nörenberg, J., Jaksó, P., and Barakonyi, A. (2021). Gamma/Delta T Cells in the Course of Healthy Human Pregnancy: Cytotoxic Potential and the Tendency of CD8 Expression Make CD56+ γδT Cells a Unique Lymphocyte Subset. Front. Immunol. *11*, 596489. https://doi.org/10.3389/fimmu.2020.596489.

Nutt, S.L., Hodgkin, P.D., Tarlinton, D.M., Corcoran, L.M., 2015. The generation of antibody-secreting plasma cells. Nat Rev Immunol 15, 160–171. https://doi.org/10.1038/nri3795

Park, J.E., Botting, R.A., Conde, C.D., Popescu, D.M., Lavaert, M., Kunz, D.J., Goh, I., Stephenson, E., Ragazzini, R., Tuck, E., et al. (2020). A cell atlas of human thymic development defines T cell repertoire formation. Science *367*. https://doi.org/10.1126/science.aay3224.

Pellicci, D.G., Koay, H.-F., and Berzins, S.P. (2020). Thymic development of unconventional T cells: how NKT cells, MAIT cells and γδ T cells emerge. Nat. Rev. Immunol. *20*, 756–770. https://doi.org/10.1038/s41577-020-0345-y.

Perdomo-Celis, F., Taborda, N.A., and Rugeles, M.T. (2017). Follicular CD8+ T Cells: Origin, Function and Importance during HIV Infection. Front. Immunol. *8*, 1241. https://doi.org/10.3389/fimmu.2017.01241.

Pfefferle, A., Netskar, H., Ask, E.H., Lorenz, S., Goodridge, J.P., Sohlberg, E., Clancy, T., Malmberg, K.-J., 2019. A Temporal Transcriptional Map of Human Natural Killer Cell Differentiation. https://doi.org/10.1101/630657

Pizzolato, G., Kaminski, H., Tosolini, M., Franchini, D.-M., Pont, F., Martins, F., Valle, C., Labourdette, D., Cadot, S., Quillet-Mary, A., et al. (2019). Single-cell RNA sequencing unveils the shared and the distinct cytotoxic hallmarks of human TCRVδ1 and TCRVδ2 γδ T lymphocytes. Proc. Natl. Acad. Sci. *116*, 11906–11915. https://doi.org/10.1073/pnas.1818488116.

Poli, A., Michel, T., Thérésine, M., Andrès, E., Hentges, F., Zimmer, J., 2009. CD56bright natural killer (NK) cells: an important NK cell subset. Immunology 126, 458–465. https://doi.org/10.1111/j.1365-2567.2008.03027.x

Provine, N.M., Binder, B., FitzPatrick, M.E.B., Schuch, A., Garner, L.C., Williamson, K.D., van Wilgenburg, B., Thimme, R., Klenerman, P., Hofmann, M. (2018). Unique and Common Features of Innate-Like Human Vδ2+ γδT Cells and Mucosal-Associated Invariant T Cells. Frontiers in Immunology 9. https://doi.org/10.3389/fimmu.2018.00756

Qi, H. (2016). T follicular helper cells in space-time. Nat. Rev. Immunol. *16*, 612–625. https://doi.org/10.1038/nri.2016.94.

Rodda, L.B., Lu, E., Bennett, M.L., Sokol, C.L., Wang, X., Luther, S.A., Barres, B.A., Luster, A.D., Ye, C.J., and Cyster, J.G. (2018). Single-Cell RNA Sequencing of Lymph Node Stromal Cells Reveals Niche-Associated Heterogeneity. Immunity *48*, 1014-1028.e6. https://doi.org/10.1016/j.immuni.2018.04.006.

Rodrigues, P.F., Alberti-Servera, L., Eremin, A., Grajales-Reyes, G.E., Ivanek, R., and Tussiwand, R. (2018). Distinct progenitor lineages contribute to the heterogeneity of plasmacytoid dendritic cells. Nat. Immunol. *19*, 711–722. https://doi.org/10.1038/s41590-018-0136-9.

Sallusto, F., Geginat, J., and Lanzavecchia, A. (2004). Central Memory and Effector Memory T Cell Subsets: Function, Generation, and Maintenance. Annu. Rev. Immunol. *22*, 745–763. https://doi.org/10.1146/annurev.immunol.22.012703.104702.

Seifert, M., Sellmann, L., Bloehdorn, J., Wein, F., Stilgenbauer, S., Dürig, J., and Küppers, R. (2012). Cellular origin and pathophysiology of chronic lymphocytic leukemia. J. Exp. Med. *209*, 2183–2198. https://doi.org/10.1084/jem.20120833.

Smeltzer, J.P., Jones, J.M., Ziesmer, S.C., Grote, D.M., Xiu, B., Ristow, K.M., Yang, Z.Z., Nowakowski, G.S., Feldman, A.L., Cerhan, J.R., et al. (2014). Pattern of CD14+ follicular dendritic cells and PD1+ T cells independently predicts time to transformation in follicular lymphoma. Clin. Cancer Res. Off. J. Am. Assoc. Cancer Res. *20*, 2862–2872. https://doi.org/10.1158/1078-0432.CCR-13-2367.

Stark, J.M., Tibbitt, C.A., and Coquet, J.M. (2019). The Metabolic Requirements of Th2 Cell Differentiation. Front. Immunol. *10*, 2318. https://doi.org/10.3389/fimmu.2019.02318.

Strauchen, J.A., Miller, L.K., 2003. Lymphoid progenitor cells in human tonsils. Int J Surg Pathol 11, 21–24. https://doi.org/10.1177/106689690301100105

Szabo, P.A., Levitin, H.M., Miron, M., Snyder, M.E., Senda, T., Yuan, J., Cheng, Y.L., Bush, E.C., Dogra, P., Thapa, P., Farber, D.L., Sims, P.A., 2019. Single-cell transcriptomics of human T cells reveals tissue and activation signatures in health and disease. Nat Commun 10, 4706. https://doi.org/10.1038/s41467-019-12464-3

Takahashi, K., Sivina, M., Hoellenriegel, J., Oki, Y., Hagemeister, F.B., Fayad, L., Romaguera, J.E., Fowler, N., Fanale, M.A., Kwak, L.W., Samaniego, F., Neelapu, S., Xiao, L., Huang, X., Kantarjian, H., Keating, M.J., Wierda, W., Fu, K., Chan, W.C., Vose, J.M., O'Brien, S., Davis, R.E., Burger, J.A., 2015. CCL3 and CCL4 are biomarkers for B cell receptor pathway activation and prognostic serum markers in diffuse large B cell lymphoma. Br J Haematol 171, 726–735. https://doi.org/10.1111/bjh.13659

Travaglini, K.J., Nabhan, A.N., Penland, L., Sinha, R., Gillich, A., Sit, R.V., Chang, S., Conley, S.D., Mori, Y., Seita, J., et al. (2020). A molecular cell atlas of the human lung from single-cell RNA sequencing. Nature *587*, 619–625. https://doi.org/10.1038/s41586-020-2922-4.

Villani, A.-C., Satija, R., Reynolds, G., Sarkizova, S., Shekhar, K., Fletcher, J., Griesbeck, M., Butler, A., Zheng, S., Lazo, S., et al. (2017). Single-cell RNA-seq reveals new types of human blood dendritic cells, monocytes, and progenitors. Science *356*, eaah4573. https://doi.org/10.1126/science.aah4573.

Vivier, E., Artis, D., Colonna, M., Diefenbach, A., Santo, J.P.D., Eberl, G., Koyasu, S., Locksley, R.M., McKenzie, A.N.J., Mebius, R.E., Powrie, F., Spits, H., 2018. Innate Lymphoid Cells: 10 Years On. Cell 174, 1054–1066. https://doi.org/10.1016/j.cell.2018.07.017

Walsh, D.A., da Silva, H.B., Beura, L.K., Peng, C., Hamilton, S.E., Masopust, D., Jameson, S.C., 2019. The functional requirement for CD69 in establishment of resident memory CD8+ T cells varies with tissue location. J Immunol 203, 946–955. https://doi.org/10.4049/jimmunol.1900052

Williams, D.W., Greenwell-Wild, T., Brenchley, L., Dutzan, N., Overmiller, A., Sawaya, A.P., Webb, S., Martin, D., NIDCD/NIDCR Genomics and Computational Biology Core, Hajishengallis, G., et al. (2021). Human oral mucosa cell atlas reveals a stromal-neutrophil axis regulating tissue immunity. Cell *184*, 4090-4104.e15. https://doi.org/10.1016/j.cell.2021.05.013.

Wing, J.B., Kitagawa, Y., Locci, M., Hume, H., Tay, C., Morita, T., Kidani, Y., Matsuda, K., Inoue, T., Kurosaki, T., et al. (2017). A distinct subpopulation of CD25 $^-$ T-follicular regulatory cells localizes in the germinal centers. Proc. Natl. Acad. Sci. *114*. https://doi.org/10.1073/pnas.1705551114.

Wragg, K.M., Tan, H.-X., Kristensen, A.B., Nguyen-Robertson, C.V., Kelleher, A.D., Parsons, M.S., Wheatley, A.K., Berzins, S.P., Pellicci, D.G., Kent, S.J., Juno, J.A. (2020). High CD26 and Low CD94 Expression Identifies an IL-23 Responsive Vδ2+ T Cell Subset with a MAIT Cell-like Transcriptional Profile. Cell Reports 31, 107773. https://doi.org/10.1016/j.celrep.2020.107773

Wu, C., Boey, D., Bril, O., Grootens, J., Vijayabaskar, M.S., Sorini, C., Ekoff, M., Wilson, N.K., Ungerstedt, J.S., Nilsson, G.P., et al. (2022a). Single-cell transcriptomics reveals the identity and regulators of human mast cell progenitors. Blood Adv. bloodadvances.2022006969. https://doi.org/10.1182/bloodadvances.2022006969.

Wu, Z., Zheng, Y., Sheng, J., Han, Y., Yang, Y., Pan, H., and Yao, J. (2022b). CD3+CD4-CD8- (Double-Negative) T Cells in Inflammation, Immune Disorders and Cancer. Front. Immunol. *13*, 816005. https://doi.org/10.3389/fimmu.2022.816005.

Yang, B.-H., Wang, K., Wan, S., Liang, Y., Yuan, X., Dong, Y., Cho, S., Xu, W., Jepsen, K., Feng, G.-S., Lu, L.-F., Xue, H.-H., Fu, W., 2019. TCF1 and LEF1 Control Treg Competitive Survival and Tfr Development to Prevent Autoimmune Diseases. Cell Rep 27, 3629-3645.e6. https://doi.org/10.1016/j.celrep.2019.05.061

Yoon, S.-O., Zhang, X., Lee, I.Y., Spencer, N., Vo, P., and Choi, Y.S. (2013). CD9 is a novel marker for plasma cell precursors in human germinal centers. Biochem. Biophys. Res. Commun. *431*, 41–46. https://doi.org/10.1016/j.bbrc.2012.12.102.

Zhu, X., and Zhu, J. (2020). CD4 T Helper Cell Subsets and Related Human Immunological Disorders. Int. J. Mol. Sci. *21*, 8011. https://doi.org/10.3390/ijms21218011.
